# Phosphite inhibits *Phytophthora cinnamomi* by downregulating oxidoreductases and disrupting energy metabolism

**DOI:** 10.3389/fmicb.2025.1632726

**Published:** 2025-08-25

**Authors:** S. Ashok Prabhu, Previn Naicker, Tuan A. Duong, Ireshyn Selvan Govender, Juanita Engelbrecht, Robert Backer, Stoyan Hristov Stoychev, Noëlani van den Berg

**Affiliations:** ^1^Hans Merensky Chair in Avocado Research, University of Pretoria, Pretoria, South Africa; ^2^Department of Biochemistry, Genetics and Microbiology, Faculty of Natural and Agricultural Sciences, Forestry and Agricultural Biotechnology Institute, University of Pretoria, Pretoria, South Africa; ^3^ReSyn Biosciences, Edenvale, South Africa; ^4^Future Production Chemicals, Council for Scientific and Industrial Research, Pretoria, South Africa; ^5^Evosep Aps, Odense, Denmark

**Keywords:** SWATH-MS, phosphite, *Phytophthora cinnamomi*, oxidoreductases, metabolism, antioxidants, mitochondrial translation, membrane proteins

## Abstract

Phytophthora root rot caused by the hemibiotrophic oomycete, *Phytophthora cinnamomi* is a major biotic hindrance in meeting the ever-increasing demand for avocados. In addition, the pathogen is a global menace to agriculture, horticulture and forestry. Phosphite trunk injections and foliar sprays remain the most effective chemical management strategy used in commercial avocado orchards against the pathogen. Phosphite is known to counter *P. cinnamomi* both directly and indirectly through fortification of host defense. However, phosphite's direct mode of action is still not understood completely. This study identified a *P. cinnamomi* isolate GKB4 sensitive to phosphite (EC_50_ of 27.9 μg/mL) and investigated the direct impact of phosphite on this isolate through label-free quantitative SWATH-MS. Proteomics data analysis of untreated vs. phosphite-treated samples revealed that the xenobiotic affects the pathogen's growth by targeting the oxidoreductases whose abundance is significantly reduced. Further, perturbations in the energy metabolism and membrane/transmembrane proteins and transporters, and oxidative stress contribute to growth inhibition. The current study also identified increased putrescine biosynthesis, a polyamine, that when present at non-optimal concentrations could be cytostatic/cytotoxic. The differential expression of enzymes involved in the biosynthesis of secondary metabolites and the intermediates/precursors involved in their biosynthesis is an interesting finding that needs further investigation to ascertain their role in phosphite-induced stress. The pathogen's attempt to counter phosphite's growth-inhibitory effects—through upregulation of alternate bioenergetics pathways (amino acid catabolism and β-oxidation of fatty acids), mitochondrial translation and translocation machinery, peroxisomal proteins, and antioxidants—appears ineffective. This research furthers our limited understanding of the direct *in vitro* effects of phosphite on *P. cinnamomi* and has identified potential candidates for molecular functional investigation.

## Highlights

- A phosphite-sensitive *P. cinnamomi* isolate (*Pc*GKB4) was identified.
- Label-free quantitative proteomics identified that phosphite inhibits *Pc*GKB4 predominantly by downregulating oxidoreductases and glycolysis.
- Phosphite increased the biosynthesis of putrescine. Depending on its intracellular levels it could promote growth or act as a cytostatic/cytotoxic agent.
- Enhanced production of proteins involved in alternative energy metabolism, mitochondrial translation, peroxisomal proteins and antioxidants is a futile countereffort by the pathogen to revive growth.

## 1 Introduction

Avocado (*Persea americana* Mill.) is rich in healthy fats, fiber, vitamins, minerals and antioxidants making it a popular superfood. Regular consumption is linked to various health benefits, including improved gut health, weight management, reduced risk of heart disease, and protection against age-related macular degeneration (Dreher and Davenport, 2013). Consequently, there is an ever-increasing demand for avocados around the world. In 2023, the global market was valued at $15.9 billion and is projected to grow to $30.22 billion by 2032 (https://www.zionmarketresearch.com/report/avocado-market).

One of the major biotic constraints to sustained avocado production is Phytophthora root rot (PRR), caused by the hemibiotrophic oomycete pathogen, *Phytophthora cinnamomi* Rands (Hardham and Blackman, 2018). The pathogen infects avocado feeder roots impairing water and nutrient transport, leading to severe wilting and tree mortality. PRR can result in 45–90% reduction in fruit production, depending on disease severity (Pérez-Jiménez, 2008). In addition, *P. cinnamomi* poses a global threat, affecting more than 5,000 plant species across agriculture, horticulture, and forestry (Hardham and Blackman, 2018). Though commercial avocado orchards typically employ integrated PRR management which includes chemical treatments, mulching, soil solarization, good nursery practices, antagonistic microbial formulations, and partially resistant rootstocks; chemical phosphite (Phi) trunk injections or foliar sprays remain the most effective intervention (Boulle et al., 2023; Wolstenholme and Sheard, 2010).

Phosphonic acid-based compounds, including ammonium, potassium, and sodium phosphonates, fosetyl-Al, and more recently, calcium chelates have demonstrated efficacy in managing oomycete disease (Khdiar et al., 2022). In addition to controlling oomycetes such as *Plasmopara, Phytophthora*, and *Pythium*, phosphonic acid formulations have also been effective against bacteria like *Pseudomonas syringae*, parasitic nematodes in wheat and oat, and fungal pathogens such as *Puccinia emaculata* and *Phakopsora pachyrhizi*, the causal agents of switchgrass rust and Asian soybean rust, respectively (Bultreys et al., 2018; Dann and McLeod, 2021; Gill et al., 2018; Oka et al., 2007). Phosphite (${\text{HPO}}_{3}^{2-}$), a dissociation byproduct of phosphonic acid-based formulations, is considered the bioactive factor responsible for pathogen control in host plants (Guest and Grant, 1991). Due to its systemic nature, long-term presence in plants, low toxicity to mammals, environmental compatibility, and complex mode of action, Phi has been widely used for oomycete control for over four decades (Dann and McLeod, 2021; Guest and Grant, 1991).

Phi treatment remains the most effective and widely adopted strategy for managing the devastating root rot pathogen *P. cinnamomi* in commercial avocado orchards worldwide. It is also a key approach in the control of *Phytophthora* responsible for tree decline in natural ecosystems (Hunter et al., 2024). However, prolonged use of Phi has led to both *in vitro* and *in planta* Phi tolerance in *P. cinnamomi* isolates from avocado orchards in Australia, New Zealand and South Africa (Hunter et al., 2023; Ma and McLeod, 2014). Additionally, other *Phytophthora* species, such as *P. capsici* and *P. citrophthora* (Hao et al., 2021), as well as other oomycetes such as *Bremia lactucae* and *Pseudoperonospora humuli* (Brown et al., 2004; Gent et al., 2020) have also developed insensitivity to Phi. This emerging Phi tolerance has significant implications for disease management in commercial settings, underscoring the need to understand both the direct and indirect modes of action of Phi on *P. cinnamomi*.

In host-oomycete interactions, Phi functions at multiple levels. At lower concentrations, it activates plant defense responses, while at higher concentrations, it exerts a direct antimicrobial and fungistatic effect. It also modulates host-pathogen interactions by stimulating the release of pathogen elicitors and reducing the production of host defense suppressors (Dunstan et al., 1990; Eshraghi et al., 2011; Guest and Grant, 1991; Jackson et al., 2000; Perez et al., 1995). More recently, Phi has been shown to protect *Rhododendron* species against PRR indirectly by altering the rhizosphere fungal community (Liu et al., 2023). Despite its effectiveness, Phi can be phytotoxic, potentially causing growth abnormalities and reducing reproductive capacity in plants (Hardy et al., 2001).

Studies across various plants, including *Eucalyptus, Arabidopsis*, cowpea, tobacco, austral grasstree, and citrus have demonstrated that Phi induces a hypersensitive response and reinforces host defense mechanisms against *Phytophthora* spp. through the accumulation of defense enzymes such as cinnamyl alcohol dehydrogenase, phenolic compounds, phytoalexins, lignin-like/lignin, hydrogen peroxide, and callose deposition (Afek and Sztejnberg, 1989; Daniel and Guest, 2006; Eshraghi et al., 2011; Jackson et al., 2000; Nemestothy and Guest, 1990; Saindrenan et al., 1988). Phi treatment in *Arabidopsis*, European beech and soybean has been shown to upregulate defense hormone pathways, such as salicylic acid, jasmonic acid (JA), and ethylene which are key regulators of plant immunity (Dalio et al., 2014; Eshraghi et al., 2011; Guo et al., 2021). In lupine and *Eucalyptus*, Phi-induced damage to root elongation zones and root tips has surprisingly been observed to benefit the plant by restricting pathogen infection and colonization (Groves et al., 2014; Jackson et al., 2000). In a recent report, Phi treatment of *Arabidopsis* suspension cells was found to primarily alter protein phosphorylation involved in core processes such as translation, RNA splicing and kinase signaling, suggesting a role in post-translational regulatory mechanisms (Mehta et al., 2021).

Compared to the extensive research on the effects of Phi in plants, fewer studies have focused on its direct mode of action in pathogenic fungi and oomycetes. Previous research has revealed several mechanisms through which Phi affects these pathogens, including targeting the adenylate pool, altering enzyme phosphorylation, repressing phosphate (Pi)-repressible acid phosphatase, increasing pyrophosphate levels, and disrupting mycelial lipid metabolism and the pentose phosphate pathway (Guest and Grant, 1991). In *P. cinnamomi*, Phi treatment induces cell wall lysis, hyphal distortions, and reduced zoospore production (King et al., 2010; Wilkinson et al., 2001). The first omics-level study on the effect of Phi on *P. cinnamomi* utilized microarray analysis, identifying genes involved in cell wall synthesis, or cytoskeleton functioning (King et al., 2010). In contrast, RNA sequencing of rust pathogens *P. emaculata* and *P. pachyrhizi* did not reveal Phi-mediated disruption of cell wall biosynthesis. Instead, downregulated genes in one or both rust fungi belonged to functional categories such as ribosomal protein, actin, RNA-dependent RNA polymerase, and aldehyde dehydrogenase (Gill et al., 2018). A recent proteomics study investigating the *in vitro* effects of Phi on *P. cinnamomi* isolates demonstrated that the inhibitory effect of Phi is accomplished by interfering with metabolism, signaling and gene expression (Andronis et al., 2024). To date this is the only proteomics-level study examining the direct effect of Phi on *P. cinnamomi*. Hence, this necessitates investigations from more research groups involving different *P. cinnamomi* isolates to achieve a comprehensive understanding of Phi's direct mode of action. Building on these findings, the present study aims to deepen our understanding of Phi's direct effects on *P. cinnamomi* by utilizing advancements in mass spectrometry-based proteomics and the availability of a well-annotated *P. cinnamomi* genome generated by our research group (Engelbrecht et al., 2021; Ludwig et al., 2018).

## 2 Materials and methods

### 2.1 *Phytophthora cinnamomi* isolate

*P. cinnamomi* isolate GKB4 (*Pc*GKB4) routinely used in avocado infection experiments in our research group was obtained from the culture collection of the Avocado Research Programme, Forestry and Agricultural Biotechnology Institute (FABI), University of Pretoria, Pretoria, South Africa.

### 2.2 Phosphite stock

A 1.25% H_3_PO_3_ stock solution (99%; Sigma-Aldrich, St. Louis, Missouri, United States) was prepared in deionized water, adjusted to pH 6.0 with 1 M KOH, and filter-sterilized through 0.22 μm syringe filters (Sigma-Aldrich) and stored at 4°C. A working solution was prepared by diluting the stock 1:20 in deionized water, followed by pH verification, filter sterilization, and storage at 4°C.

### 2.3 Preparation of *Pc*GKB4 inoculum

The *Pc*GKB4 stock culture was sub-cultured onto ½ PDA plates (19.5 g/L potato dextrose agar and 7.5 g/L agar) and incubated in the dark at 22°C for 4 days. A 5 mm × 5 mm mycelial plug was excised and inoculated into a Granny Smith apple, which was then wrapped with cling film, placed in a zip-lock bag, and incubated in the dark at 22°C for 5 days (Ribeiro et al., 1975). A 5 mm × 5 mm section of infected apple tissue was subsequently sub-cultured onto *Phytophthora*-selective NARPH agar plates (19.5 g/L PDA, amended with 50 mg/L nystatin, 200 mg/L ampicillin, 100 mg/L pentachloronitrobenzene, 10 mg/L rifampicin and 50 mg/L hymexazol) and incubated at 22°C for 2 days in the dark (Hüberli et al., 2000). Single hyphal tips were transferred onto modified Ribeiro's minimal media (RMM) agar plates supplemented with 10 mM KH_2_PO_4_ (Ribeiro et al., 1975) and incubated at 22°C for 7 days in the dark. Molecular confirmation of the single hyphal tip cultures was performed by amplifying the species-specific *LPV3* fragment following the protocol described in Engelbrecht et al. (2013). Actively growing hyphae were inoculated into 200 mL RMM broth containing 10 mM KH_2_PO_4_ and incubated with agitation at 115 rpm in the dark at 22°C for 5 days. Mycelia were harvested by vacuum filtration using an MD 4 NT VARIO vacuum pump (Vacuubrand, Sigma-Aldrich) and homogenized on ice with a hand blender (Russel Hobbs, Oldham, United Kingdom) at maximum speed for five cycles of 1 min blending with 1 min rest intervals. The optical density (A_600nm_) of the mycelial homogenate was adjusted to 0.5 with sterile RMM broth containing 10 mM KH_2_PO_4_, serving as the inoculum.

### 2.4 Effect of Phi on *Pc*GKB4 growth—dose-response curve analysis

A total of 54 sterile 100 mL conical flasks, each containing 25 mL of RMM broth supplemented with 10 mM KH_2_PO_4_ and Phi at concentrations of 0, 0.125, 0.25, 0.5, 0.75, 1, 1.25, 2.5, 5, 10, 20, 40, 60, 80, 100, 200, 500, and 1,000 μg/mL in triplicates, were inoculated with 125 μL of the above prepared inoculum. Cultures were incubated at 22°C in the dark with agitation at 150 rpm. Mycelia were harvested by vacuum filtration 6 days post inoculation (dpi), transferred to 15 mL conical polypropylene tubes, snap frozen in liquid nitrogen, and stored at −80°C until further use. Lyophilisation was performed for 24 h, and the mycelial dry mass was measured. The experiment was repeated thrice each with triplicate samples. The effective concentration of Phi required to inhibit *Pc*GKB4 growth by 50% (EC_50_) was determined using a four-parameter log-logistic model implemented in the *drc* package in R (v4.3.1) (Ritz et al., 2015).

### 2.5 *Pc*GKB4 mycelial protein extraction

Mycelia were harvested by vacuum filtration from six bulk cultures (400 mL RMM broth in 2 L conical flasks) of both untreated controls (no Phi) and Phi-treated (30 μg/mL) cultures separately, grown for 6 dpi at 22°C in the dark with shaking at 150 rpm, lyophilized and stored at −80°C until further use. The Phi concentration was chosen based on the EC_50_ values determined by dose-response curve analysis (Ritz et al., 2015). For protein extraction, 1 g of mycelia was ground in a pre-cooled pestle and mortar with liquid nitrogen to a fine powder. The powder was resuspended in 5 volumes of protein extraction buffer (50 mM Tris-HCl, pH 8.0, 2% SDS; 10 mM DTT, protease-inhibitor (Thermo Fisher Scientific, Waltham, Massachusetts, United States) and PhosSTOP (Sigma-Aldrich) added to the extraction buffer just before use in a 50 mL polypropylene conical tube (Eppendorf, Hamburg, Germany), vortexed for 30 s and incubated at 65°C for 30 min. Thereafter, the homogenate was intermittently vortexed for 30 s every 10 min, and cooled on ice prior to centrifugation at 15,000 × *g* at 4°C for 30 min. The supernatant was transferred to a fresh tube, and an equal volume of UltraPure™ Buffer-Saturated Phenol, pH 7.4 (Invitrogen, Waltham, Massachusetts, United States) was added and incubated on ice for 30 min agitated at 150 rpm. The samples were centrifuged again at 15,000 × *g* at 4°C for 30 min. The upper phase was discarded and to the lower phenolic phase an equal volume of extraction buffer was added, and the mixture was incubated on ice for 30 min with shaking at 150 rpm. The samples were centrifuged at 15,000 × *g* at 4°C, 30 min, and the lower phenolic phase was transferred to a fresh tube. Protein precipitation was performed by adding 5 volumes of ice-cold acetone and incubating at −20°C, overnight. The samples were centrifuged at 15,000 × *g* at 4°C, 30 min, and pellets were washed twice with 10 mL ice-cold acetone by resuspension, followed by centrifugation at 15,000 × *g* at 4°C for 30 min. The pellet was air-dried in a fume hood for 2 min and resuspended in a minimum volume of protein solubilisation buffer (2% SDS in 20 mM Tris-HCl buffer, pH 8.0 amended with protease-inhibitor and PhosSTOP). Protein concentration was measured using the Pierce Bicinchoninic assay (Thermo Fisher Scientific), as per the manufacturer's instructions, using BSA as the standard. Twenty micrograms of each sample (control and Phi-treated) were separated by SDS-PAGE (Laemmli, 1970) on a 12% polyacrylamide gel (1 mm thick) under reducing conditions. Protein bands were visualized with Coomassie blue R−250 staining (Sigma-Aldrich).

### 2.6 Protein sample preparation for mass spectrometry

Protein samples (10 μg/sample) were reduced with 5 mM Tris(2-carboxyethyl) phosphine and alkylated with 10 mM 2-chloroacetamide at room temperature for 20 min. Detergents and salts were removed using MagReSyn™ Hydroxyl beads (ReSyn Biosciences, Pretoria, South Africa) as previously described (Batth et al., 2019; Koenig et al., 2023). On-bead protein digestion was carried out for 16 h using sequencing-grade trypsin at a 1:100 protease:protein ratio. Ten micrograms of peptide digest was vacuum-dried separately for full proteome analysis.

### 2.7 LC-MS data acquisition

Approximately 1 μg of peptides per sample was analyzed using a Dionex Ultimate 3000 RSLC system (Thermo Fisher Scientific) coupled to a Sciex 5600 TripleTOF mass spectrometer. Peptides were initially desalted in-line using an Acclaim PepMap C18 trap column (75 μm × 2 cm) (Thermo Fisher Scientific) for 2 min at a flow rate of 5 μL min^−1^ with 2% acetonitrile (ACN) and 0.2% formic acid (FA). Trapped peptides were then gradient-eluted and separated on an Acquity CSH C18 NanoEase column (75 μm × 25 cm, 1.7 μm particle size) (Waters Corporation, Milford, Massachusetts, United States) at a flow-rate of 0.3 μL min^−1^ with a gradient of 6–40% B (A: 0.1% FA; B: 80% ACN with 0.1% FA) over 90 min (full proteome analysis). For Sequential Window Acquisition of all THeoretical (SWATH) mass spectra, precursor scans were acquired across a mass range of 400–1,100 m/z with an accumulation time of 50 ms. Fragment ions were collected from 200 to 1,800 m/z using 48 variable-width precursor windows with a 0.5 Da overlap between windows and an accumulation time of 20 ms per window. The mass spectrometry proteomics data has been deposited to the ProteomeXchange Consortium (Deutsch et al., 2022) via the PRIDE (Perez-Riverol et al., 2019) partner repository with the dataset identifier PXD063718.

### 2.8 Bioinformatics analysis

#### 2.8.1 LC-MS data analysis

SWATH data were processed using Spectronaut v18 (Biognosys AG, Schlieren, Switzerland). Data processing followed the default directDIA identification and quantification settings. Carbamidomethylation was set as a fixed modification, while N-terminal acetylation and methionine oxidation were considered as variable modifications. The proteotypic filter was configured to ‘Only protein group specific'. The search database included *P. cinnamomi* (Engelbrecht et al., 2021) proteins along with common contaminating proteins. A false discovery rate (FDR) threshold of 1% was applied at the precursor and protein levels. Quantification was conducted at the MS^2^ level, with label-free cross-run normalization implemented using a global normalization strategy. Proteins identified by single peptides (single hits) were excluded. The ‘Protein Quant' pivot report (excluding the decoys) was exported and contaminants were manually removed.

Downstream data analysis was performed using Perseus v2.0.11 (Tyanova et al., 2016). The protein quant data matrix was imported into Perseus, where control and Phi-treated label-free quantification (LFQ) datasets were grouped separately. Data were log_2_(x)-transformed, filtered for entries where LFQ values were available in at least 100% bio replicates among at least one of the two study groups (control, treated). Missing values were imputed using a normal distribution (width=0.3, down shift=1.8). A student's two-sample *t*-test was performed with Phi-treated samples as the first group and untreated control as the second group, S_0_ = 0.1, 250 randomizations) applying a permutation-based FDR cutoff of < 0.05. A differential abundance list was generated by filtering proteins with statistically significant Student's *t*-test values, and subsequently applying a log_2_ absolute fold-change (log_2_FC) threshold of > 0.58 or < −0.58. Principal component analysis (PCA) was performed using FactoMineR v2.11 and visualized using ggplot2 v3.5.2 packages in R (R for Windows v4.4.0). Heatmap was generated using the ComplexHeatmap package (Gu, 2022) in R (R for Windows v4.4.0).

#### 2.8.2 Full STRING association network and functional enrichment analysis

Full STRING association networks were constructed for differentially expressed proteins (DEPs; log_2_FC > 0.58 or < −0.58) using StringDB v12.0, applying a minimum confidence threshold of 0.7. Node size and color were scaled according to log_2_FC values to visually represent protein expression changes. Functional networks were visualized in Cytoscape v3.10.2 (Shannon et al., 2003; Szklarczyk et al., 2023). Gene Ontology (GO), KEGG (Kyoto Encyclopedia of Genes and Genomes) pathway, annotated keyword (UniProt), and reactome pathway enrichment analyses were conducted separately for significantly up- (log_2_FC > 0.58) and down-regulated (log_2_FC < −0.58) proteins. These analyses were performed against the background list of all expressed proteins using the ‘Analysis' tab in the STRING-DB v12.0, with default settings and an FDR threshold of < 0.05.

**Figure 1:**
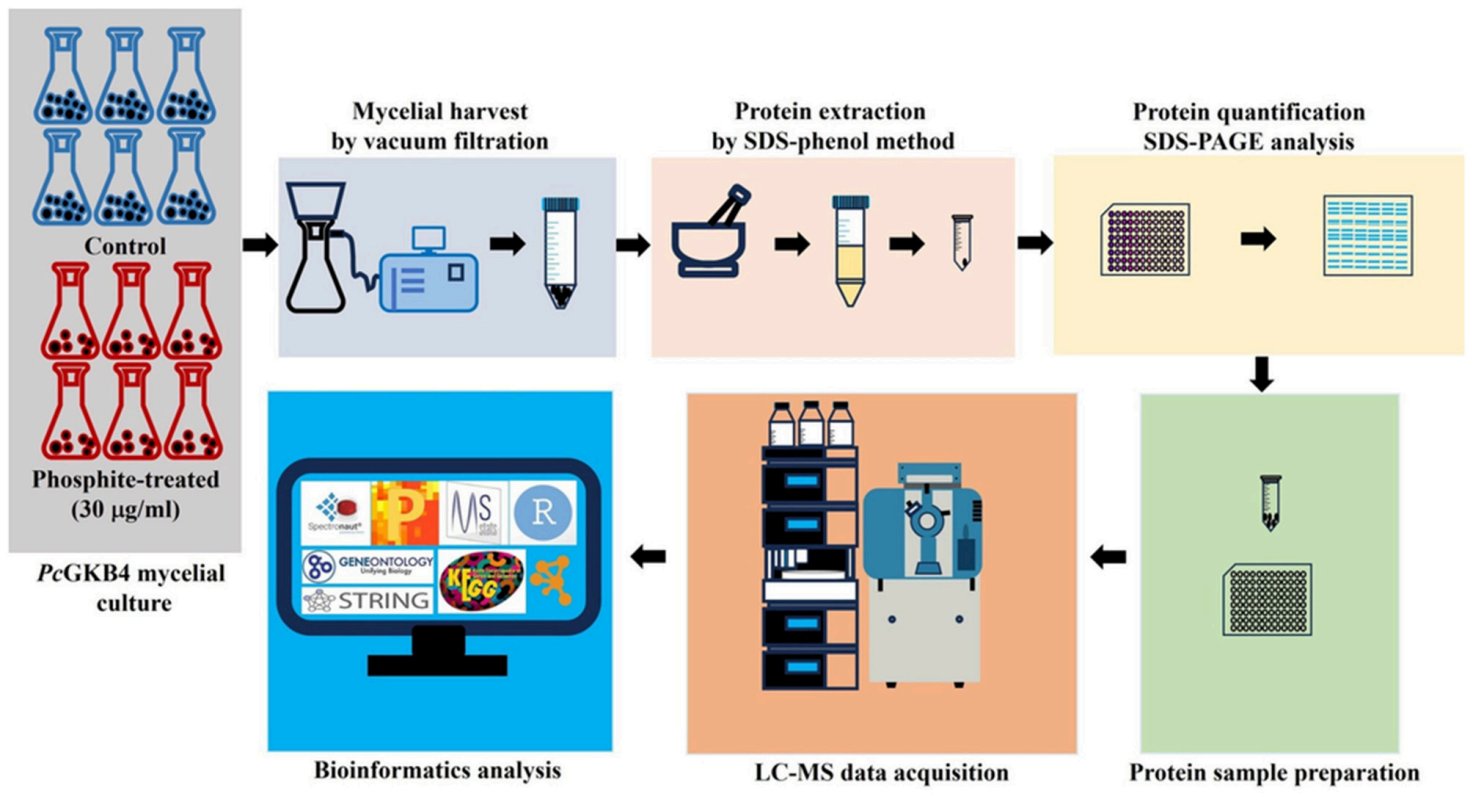
Schematic representation of experimental outline.

A schematic representation of the experimental strategy is outlined in Figure 1.

## 3 Results and discussion

The current study aimed to address Phi's direct mode of action in *P. cinnamomi*—using a label-free quantitative proteomics approach. This research is particularly significant as only a single proteomics study has been conducted on this topic to date (Andronis et al., 2024).

### 3.1 *Pc*GKB4 is a phosphite-sensitive isolate

**Figure 2:**
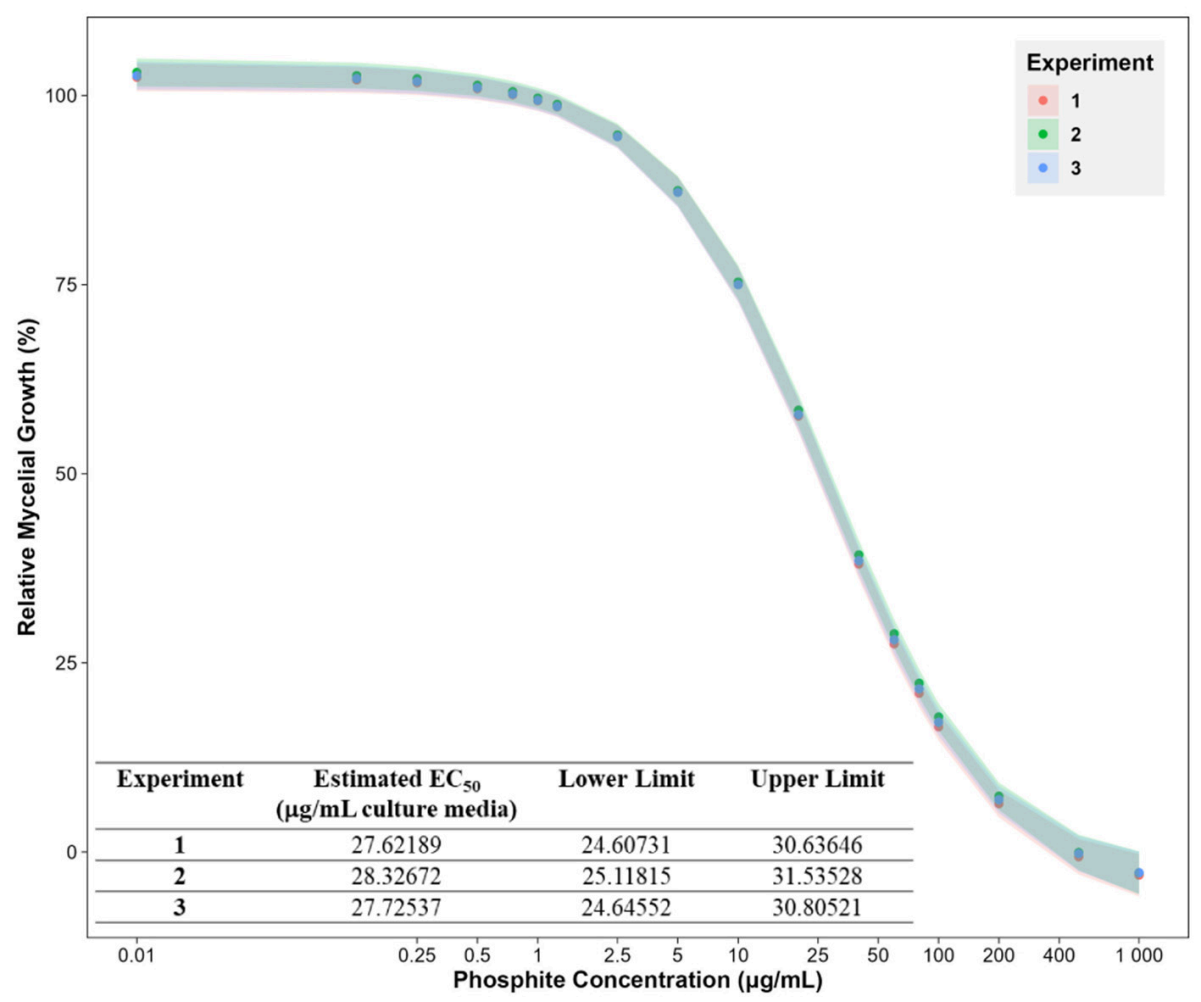
Phosphite dose response curves showing the mycelial growth of *Phytophthora cinnamomi* isolate GKB4 at different phosphite concentrations (0, 0.125, 0.25, 0.5, 0.75, 1, 1.25, 2.5, 5, 10, 20, 40, 60, 80, 100, 200, 500, and 1,000 μg/mL media) relative to untreated control. The effect of Phi on mycelial growth was tracked by measuring the mycelial dry mass 6 days post inoculation. The experiment was repeated thrice. The data points represent the mean and bars represent the 5% confidence intervals for a four-parameter log-logistic model using the R package—*drc* (Ritz et al., 2015; R for Windows v4.4.0). X-axis: log_10_() transformed. Phosphite concentration of 0 μg/mL (untreated control) is represented as 0.01 to depict on the log scale.

The Phi inhibition studies were conducted by measuring the mycelial dry mass of *Pc*GKB4 grown for 6 dpi in liquid cultures supplemented with varying Phi concentrations (0–1,000 μg/mL media). The mycelial growth decreased with increasing Phi concentrations (Figure 2). Dose-response curve analysis confirmed that *Pc*GKB4 is sensitive to Phi, consistent with previous reports, with an average EC_50_ of 27.9 μg/mL (lower limit: 24.8 μg/mL; upper limit: 31 μg/mL) (Figure 2) (Andronis et al., 2024; Hunter et al., 2018). For proteomics analysis, six biological replicates were prepared for both untreated controls (no Phi included in culture media) and Phi-treated samples, using a sub-lethal dose of 30 μg/mL. This concentration was chosen to ensure sufficient mycelial mass for total protein extraction. Changes in protein abundance were subsequently quantified using label-free SWATH mass spectrometry.

### 3.2 *In vitro* Phi treatment of *Pc*GKB4 leads to changes in protein abundance

Liquid chromatography-high resolution tandem mass spectrometry was utilized to evaluate Phi-induced proteome changes (Figure 1). Data analysis identified 1,973 protein groups and 17,165 peptides in the proteomics dataset. Differential abundance analysis using Perseus, applying a Student's *t*-test significance filter, yielded 1,075 protein groups (Supplementary Table S1). Among these, 315 protein groups (log_2_FC > 0.58 or < −0.58, *q*-value < 0.05) were classified as significantly differentially expressed proteins (DEPs) (Supplementary Figure S1). Of these, 142 protein groups were downregulated, while 173 protein groups were upregulated following Phi treatment.

**Figure 3:**
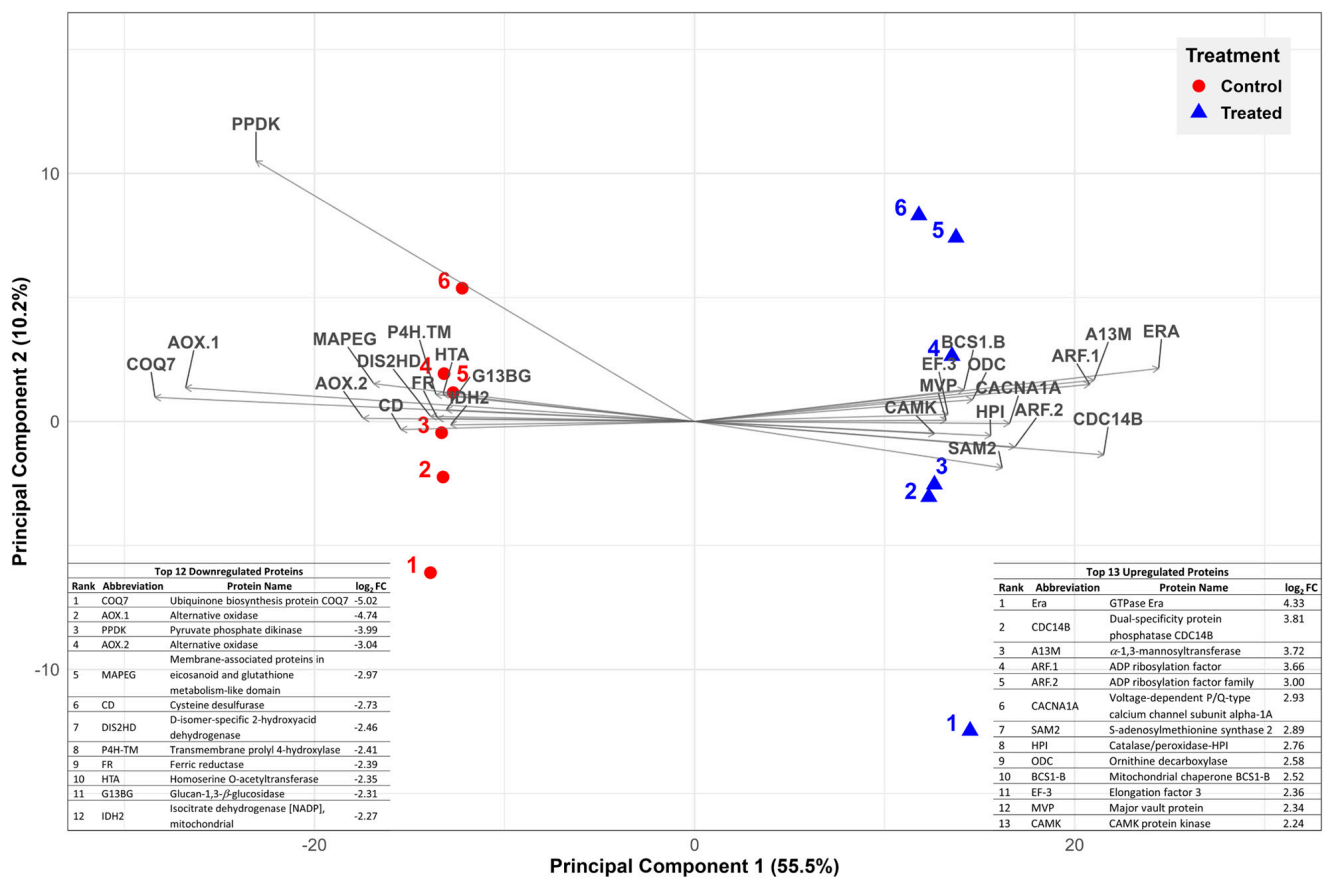
Principal component analysis (PCA) representing variation in protein abundance of untreated control (solid circles in red) vs. Phi-treated (30 μg/mL media; solid triangles in blue) quantitative protein data. The symbols (solid circles and triangles) and their associated labels represent biological replicates. The top 25 most significant variables are displayed. They correspond to top 13 upregulated (right hand side) and top 12 downregulated proteins (left hand side).

PCA of the proteomics dataset revealed distinct clustering between untreated and Phi-treated samples (Figure 3).

### 3.3 STRING-DB network and functional enrichment analysis

#### 3.3.1 Oxidoreductases downregulation vs. overrepresentation of carboxylic acid catabolic process and mitochondrial proteins

GO enrichment analysis of downregulated proteins revealed the overrepresentation of only oxidoreductases (GO:0016491, GO:0016614 and GO:0016616) belonging to the molecular functions category (Supplementary Table S2). Oxidoreductases are essential redox enzymes that mediate biological oxidation-reduction reactions, playing a key role in cell survival, growth, virulence, and reproduction. They are involved in numerous cellular processes such as energy generation through glycolysis and the tricarboxylic acid (TCA) cycle, fatty acid and amino acid metabolism, mitochondrial electron transport, and oxidative stress regulation (El-Gendi et al., 2022). The Phi-induced suppression of oxidoreductases suggests a significant disruption in *P. cinnamomi* cellular metabolism and redox homeostasis, which may contribute to its significantly reduced growth.

In contrast, GO enrichment analysis of upregulated proteins showed an overrepresentation of the biological processes related to small molecule catabolic process (GO:0044282) and carboxylic acid catabolic process (GO:0046395) as well as the mitochondrial cellular component (GO:0005739) (Supplementary Table S3). The small molecule/carboxylic acid catabolic category mostly included proteins involved in fatty acid and amino acid catabolism, while the mitochondrial component comprised of proteins involved in protein translation and translocation in addition to fatty acid and amino acid catabolism.

#### 3.3.2 Changes in metabolism and mitochondrial protein homeostasis

**Figure 4:**
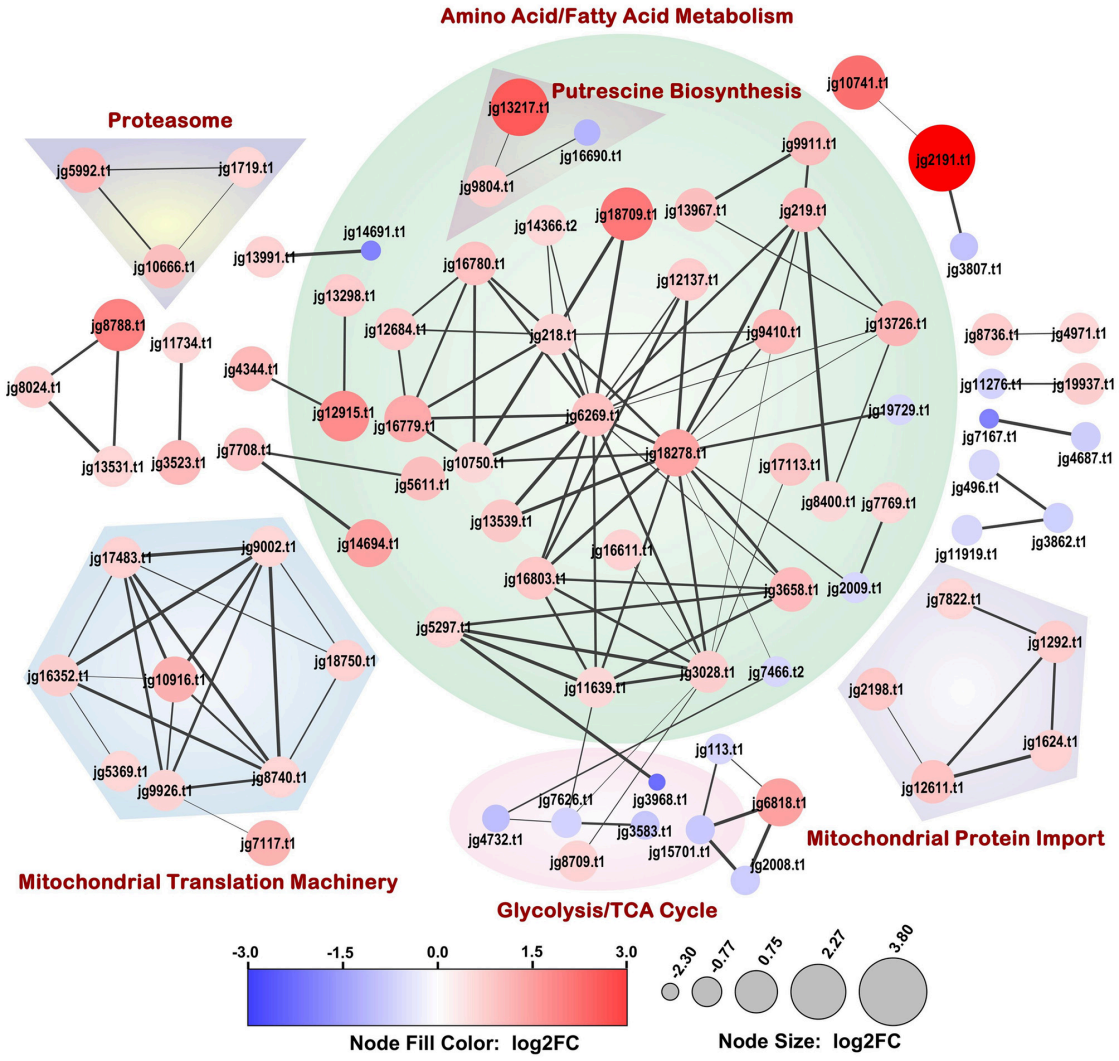
Full STRING-DB association network of proteins significantly differentially expressed upon Phi-treatment (30 μg/mL media). The size and color of the nodes were scaled with log_2_ fold change (log_2_FC) values. The association confidence between connected nodes is represented by edge thickness and a minimum confidence threshold of 0.7 was considered. Dappled areas portray the broader process relationships between associating proteins. Disconnected nodes are not depicted.

STRING-DB association network analysis of the DEPs identified 119 protein-protein interactions with a highly significant enrichment *p*-value (< 1.0E-16) (Figure 4). The major functional clusters represented in the network included amino acid and fatty acid catabolism, putrescine biosynthesis, glycolysis/TCA cycle, mitochondrial ribosome assembly and translocation, and the proteasome. Most proteins within these clusters were upregulated, except for some glycolysis cycle/TCA cycle enzymes, which were downregulated.

KEGG enrichment analysis of both the down- and up-regulated proteins highlighted the common terms—metabolism (KEGG pathway ID: map01100) and biosynthesis of secondary metabolites (KEGG pathway ID: map01110) (Supplementary Tables S2, S3). The upregulated proteins showed significant enrichment in pathways related to valine, leucine and isoleucine degradation (KEGG pathway ID: map00280) and fatty acid metabolism (KEGG pathway ID: map01212). In addition, the reactome pathways: mitochondrial protein import (Reactome pathway ID: MAP-1268020), and metabolism (Reactome pathway ID: MAP-1430728) were enriched.

Among the downregulated pathways carbon metabolism (KEGG pathway ID: map01200), glycolysis/gluconeogenesis (KEGG pathway ID: map00010), pyruvate metabolism (KEGG pathway ID: map00620), and fructose and mannose metabolism (KEGG pathway ID: map00051) were overrepresented (Supplementary Table S2). The downregulation of the enzymes involved in fructose and mannose metabolism may be attributed to the use of RMM in this experiment, where glucose is the sole carbon source. In addition, overrepresentation of the UniProt annotated keywords—membrane (Keyword ID: KW-0472), transmembrane (Keyword ID: KW-0812), and transmembrane helix (Keyword ID: KW-1133) were observed for the downregulated proteins.

##### 3.3.2.1 Perturbation in bioenergetics

Cellular energy production primarily relies on glucose catabolism via the glycolytic pathway, where pyruvate is generated and subsequently oxidized to produce the high-energy electron carriers FADH_2_ and NADH. Finally, ATP is synthesized in the mitochondria via oxidative phosphorylation in the electron transfer chain through chemiosmosis. Phi treatment resulted in the downregulation of key bioenergetic proteins such as mitochondrial isocitrate dehydrogenase (log_2_FC = −2.27), an important TCA cycle enzyme, and COQ7 (log_2_FC = −5.02) that regulates the electron transfer chain by influencing the synthesis of the key electron carrier CoQ. In addition, cytochrome c (log_2_FC = −0.49), cytochrome c oxidase (log_2_FC = −0.49) and its assembly protein COX15 (log_2_FC = −1.05), involved in the final step of ATP generation, also showed reduced abundance. Interestingly, previous studies have shown that downregulation of mitochondrial respiration components such as COQ7, cytochrome b5 and, NADH dehydrogenase was a major factor contributing to mycelial growth inhibition in *Phytophthora infestans* treated with tagatose, a rare sugar, while *P. cinnamomi* showed no such growth inhibition (Chahed et al., 2021). The observed depletion of mitochondrial respiration-related proteins in *P. cinnamomi* following Phi treatment suggests a potential mechanism by which Phi disrupts energy metabolism, ultimately impacting pathogen growth and survival.

In our study, two alternate oxidases (AOXs) were significantly downregulated (log_2_FC = −4.74 and log_2_FC = −3.04). When the cytochrome c pathway is disrupted, the mitochondria activate the AOX pathway to maintain redox equilibrium and prevent excess accumulation of reactive oxygen species (ROS), which can be detrimental to the cell. AOX, functions by bypassing mitochondrial complexes III and IV while partially maintaining ATP production through enhanced activity of complex I (Vanlerberghe, 2013). Conversely, the downregulation of AOXs may indicate an energy trade-off. As AOX activity is known to reduce ATP production. In the fungus *Blastocladiella emersonii*, mitochondrial AOX was shown to be essential for mycelial growth and sporulation (Luevano-Martinez et al., 2019). Given its critical role in fungal metabolism, targeting AOX and mitochondrial complex I could be a promising strategy for targeted control of *P. cinnamomi*. AOX*-*targeting *N*-phenylbenzamide derivatives have shown efficacy against fungal phytopathogens such as *Moniliophthora perniciosa, Sclerotinia sclerotiorum* and *Venturia pirina* (Barsottini et al., 2019). Similarly, novel peptides P-113Du and P-113Tri, which target mitochondrial complex I, have been reported to effectively control the human fungal pathogen *Candida albicans* (Xue et al., 2019). Consistent with our findings, King et al. (2010) also reported significant downregulation of AOX and pyruvate phosphate dikinase transcripts in Phi-treated *P. cinnamomi*. This further supports the idea that Phi disrupts mitochondrial energy metabolism, potentially making AOX and complex I attractive targets for disease control strategies in *P. cinnamomi*.

**Figure 5:**
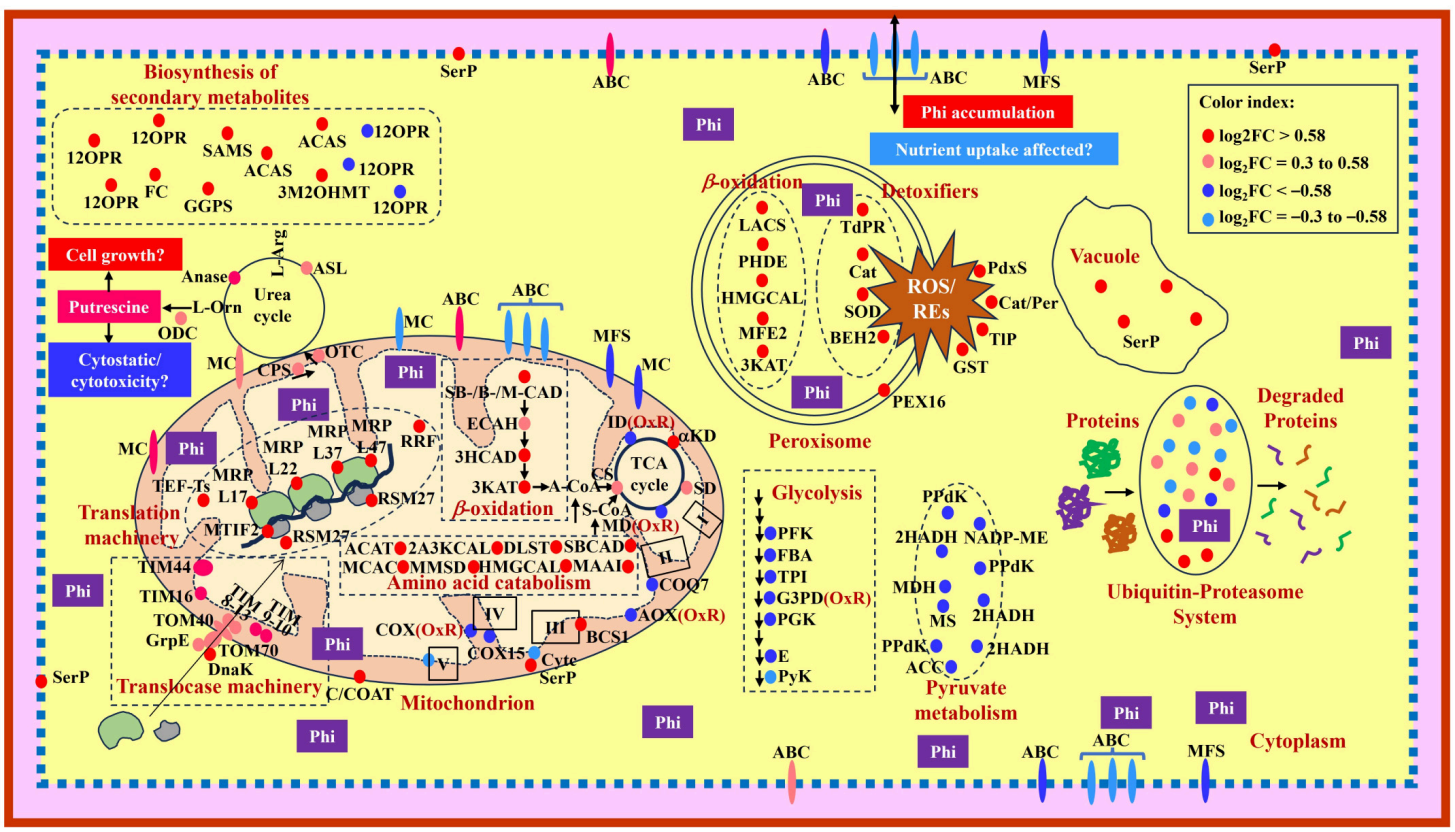
Model showing a summary of changes observed in protein abundance in *Phytophthora cinnamomi* isolate GKB4 upon Phi treatment. Glycolysis: PFK, 6-phosphofructo-2-kinase/fructose-2,6-bisphosphatase; FBA, fructose-bisphosphate aldolase; TPI, triosephosphate isomerase; G3PD, glyceraldehyde-3-phosphate dehydrogenase; PGK, phosphoglycerate kinase; E, enolase; PyK, pyruvate kinase. Transmembrane transporters: MC, mitochondrial carrier; ABC, ATP-binding cassette transporter superfamily; MFS, major facilitator superfamily. Mitochondrion—TCA cycle: αKD, α-ketoglutarate dehydrogenase subunit; SD, succinate dehydrogenase subunits; MD, malate-dehydrogenase; CS, citrate synthase; ID, isocitrate dehydrogenase; β-oxidation: SBCAD, short/branched chain-specific acyl-CoA dehydrogenase; SCAD, short chain-specific acyl-CoA dehydrogenase; MCAD, medium chain-specific acyl-CoA dehydrogenase; ECAH, enoyl-CoA-hydratase; 3HCAD, 3-hydroxyacyl-CoA dehydrogenase; 3KAT, 3-ketoacyl-CoA-thiolase; C/COAT, choline/carnitine-O-acyltransferase; Electron transport chain: I, complex I; II, complex II; III, complex III; IV, complex IV; V, complex V (ATP synthase subunits); COQ7, 5-demethoxyubiquinone hydroxylase; AOX, alternative oxidase; BCS1, mitochondrial protein chaperon responsible for protein folding, translocation and assemblage of the respiratory chain Complex III; Cytc, cytochrome c; COX, cytochrome c oxidase; COX15, cytochrome c oxidase assembly protein; Amino acid catabolism: ACAT, acetyl-CoA-acetyltransferase; 2A3KCAL, 2-amino-3-ketobutyrate CoA ligase; DLST, dihydrolipoyllysine-residue succinyltransferase; MCAC, β-chain of methylcrotonyl CoA carboxylase; MMSD, methylmalonate semialdehyde dehydrogenase; HMGCAL, hydroxymethylglutaryl CoA lyase; MAAI, maleylacetoacetate isomerase; OxR, oxidoreductase; A-CoA; acetyl CoA; S-CoA, succinyl CoA; Translocase machinery: TIM, translocases of inner membrane; TOM, translocases of outer membrane; DnaK, molecular chaperone; GrpE, nucleotide exchange factor essential for protein folding and in the regulation of DnaK; Translation machinery: MTIF2, mitochondrial translation initiation factor IF 2; TEF-Ts, translation elongation factor-Ts; MRP, mitochondrial ribosomal protein; RSM27, structural protein component of the mitochondrial ribosomal small subunit; RRF, ribosome recycling factor; Urea cycle/Putrescine biosynthesis: CPS, carbamoyl phosphate synthetase; OTC, ornithine carbamoyl transferase/ornithine transcarbamylase; ASL, argininosuccinate lyase; L-Arg, L-arginine; Anase, arginase; L-Orn, L-ornithine; ODC, ornithine decarboxylase. Biosynthesis of secondary metabolites: 12OPR, 12-oxophytodienoate reductase (peroxisome); SAMS, S-adenosylmethionine synthase 2 (cytosol); ACAS, acetyl-CoA synthetase/acetate-CoA ligase (mitochondria, cytosol/nucleus); GGPS, geranylgeranyl pyrophosphate synthetase (cytosol, vacuole); 3M2OHMT, 3-methyl-2-oxobutanoate hydroxymethyltransferase (cytosol, membrane); FC, ferrochelatase (matrix-facing side of the mitochondrial inner membrane). Peroxisome: PEX16, peroxin 16; Detoxifiers: TdPR, thioredoxin-dependent peroxide reductase; Cat, catalase; SOD, superoxide dismutase; BEH2, bifunctional epoxide hydrolase 2; ROS, reactive oxygen species; REs, reactive epoxides; β-oxidation: LACS, long chain acyl CoA synthetase 7; PHDE, peroxisomal hydratase dehydrogenase epimerase; HMGCAL, hydroxymethylglutaryl CoA lyase; MFE2, peroxisomal multifunctional enzyme type 2; 3KAT, 3-ketoacyl CoA thiolase A. Cytoplasmic detoxifiers: PdxS, pyridoxal biosynthesis lyase; Cat/Per, catalase/peroxidase-HPI; TlP, thioredoxin-like protein; GST, glutathione S-transferase. Pyruvate metabolism: PPdK, pyruvate phosphate dikinase (glycosome); 2HADH, D-isomer-specific 2-hydroxyacid dehydrogenase (mitochondria); NADP-ME, NADP-dependent malic enzyme (cytoplasm, mitochondria); MDH, malate dehydrogenase (cytoplasm, mitochondria, glycosome); MS, malate synthase (peroxisome, cell wall); ACC, acetyl CoA carboxylase (cytosol). Phi, phosphite; SerP, serine protease. The proteins under “Biosynthesis of secondary metabolites” and “Pyruvate metabolism” are shown in the cytosol due to space constraints and the cellular location of the proteins are presented within the parenthesis.

Most glycolytic pathway enzymes, including glyceraldehyde 3-phosphate dehydrogenase type I (log_2_FC = −1.59), triose phosphate isomerase (log_2_FC = −0.97), enolase (log_2_FC = −0.92), fructose bisphosphate aldolase (log_2_FC = −0.89), 6-phosphofructo-2-kinase (log_2_FC = −0.86), and phosphoglycerate kinase 1 (log_2_FC = −0.68) were significantly downregulated (Supplementary Table S1 and Figures 4, 5). The stress induced by Phi may have resulted in the depletion of glucose in an attempt to maintain cellular homeostasis compared to untreated controls. Alternatively, Phi could also have interfered with glucose uptake and phosphorylation. A comparative study analyzing glucose flux between control and Phi-treated samples will be necessary to determine the effect of Phi on glucose uptake/metabolism. In addition, the Phi-treatment also decreased gluconeogenesis under energy stress. Andronis et al. (2024) also reported downregulation of mitochondrial electron transport chain components and glycolytic pathway enzymes.

For continued survival *P. cinnamomi* generates energy through alternate mechanisms by tapping into cellular fatty and amino acids. This is supported by the upregulation of enzymes involved in β-oxidation of fatty acids resulting in acetyl-CoA and amino acid catabolism, which replenishes various TCA cycle intermediates, a process known as analplerosis. Enzymes involved in the β-oxidation of short/branched- and medium-chain fatty acids such as short-chain-specific acyl-CoA dehydrogenase (log_2_FC = 0.68), short/branched-chain-specific acyl-CoA dehydrogenase (log_2_FC = 2.07) and medium-chain-specific acyl-CoA dehydrogenase (log_2_FC = 1.15) showed increased abundance. Other upregulated β-oxidation pathway enzymes included acyl-CoA dehydrogenase (log_2_FC = 0.86), 3-hydroxyacyl-CoA dehydrogenase type-2(log_2_FC = 0.84), acyl-CoA dehydrogenase family member 9 mitochondrial precursor (log_2_FC = 0.67), two 3-ketoacyl-CoA-thiolases (log_2_FC = 1.39 and log_2_FC = 0.93, respectively), and enoyl-CoA-hydratase (log_2_FC = 0.47). In addition, choline/carnitine acyltransferase, critical for fatty acid transport into mitochondria for β-oxidation also displayed increased abundance (log_2_FC = 0.58), further supporting the metabolic shift toward lipid utilization for energy generation.

Enzymes involved in the degradation of amino acids such as lysine (dihydrolipoyllysine-residue succinyltransferase; log_2_FC = 1.04), leucine (hydroxymethylglutaryl CoA lyase; log_2_FC = 0.84, β-chain of methylcrotonyl-CoA carboxylase; log_2_FC = 0.96), isoleucine (short/branched chain-specific acyl-CoA dehydrogenase and acetyl-CoA-acetyltransferase; log_2_FC = 1.09), valine (methylmalonate-semialdehyde dehydrogenase; log_2_FC = 0.6), phenylalanine and tyrosine (maleylacetoacetate isomerase; log_2_FC = 0.81), and threonine (2-amino-3-ketobutyrate CoA ligase; log_2_FC = 0.58) were significantly upregulated (Supplementary Table S1 and Figures 4, 5). Two acetyl-CoA synthetases (log_2_FC = 1.32 and 0.94), which catalyze the conversion of acetate (a ketogenic amino acid degradation product) into acetyl Co-A (a Krebs cycle intermediate), was also highly abundant. In *Magnaporthe oryzae*, β-oxidation mediated by short-chain acyl-CoA dehydrogenases were shown to play a crucial role in vegetative growth, conidia production, pathogenesis, and oxidative stress adaptation (Aliyu et al., 2019). The diversion of fatty and amino acids toward energy production may, however, inhibit growth and development by disrupting protein homeostasis and membrane integrity.

##### 3.3.2.2 Downregulation of enzymes involved in pyruvate metabolism could affect various cellular processes

KEGG ontology analysis of downregulated proteins showed overrepresentation of enzymes associated with pyruvate metabolism such as pyruvate phosphate dikinase (three isoforms; log_2_FC = **–**3.99, **–**3.99, **–**3.99), D-isomer-specific 2-hydroxyacid dehydrogenase (three isoforms; log_2_FC = **–**2.46, **–**1.45, **–**0.62), NADP-dependent malic enzyme (log_2_FC = −0.83), malate dehydrogenase (log_2_FC = −0.99), malate synthase (log_2_FC = −0.62), and acetyl CoA carboxylase (log_2_FC = −0.59) (Supplementary Tables S1, S2). Pyruvate metabolism is a vital process that links glycolysis to TCA cycle, replenishment of the TCA cycle through glyoxylate cycle, and oxidative phosphorylation leading to energy production in the form of ATP. A downregulation of key enzymes involved in this central scheme hampers not just energy production but also affects various metabolic processes associated with fatty and amino acids, vitamin B6, and gluconeogenesis necessary for sustenance. Studies in bacteria, fungi and oomycetes have shown the involvement of carbon metabolism in growth and virulence. In the human bacterial pathogen *Staphylococcus aureus*, the central metabolite pyruvate is a key link between metabolism and virulence, as it enhances pathogenicity through the generation of virulence factors such as the pore-forming leucocidins (Harper et al., 2018). Regulation of carbon metabolism and Hog1 MAPK-mediated stress responses modulate sexual reproduction and filamentous growth in the sugarcane smut disease causing fungus, *Sporisorium scitamineum* (Yan et al., 2016). Transcriptomics studies in the highly pathogenic *P. infestans* strain DL04 showed that higher growth rate and enhanced pathogenicity linked to the virulence factor 3 expression was associated with enrichment of carbon metabolism, glycolysis/gluconeogenesis and amino acid biosynthesis (Deng et al., 2025). Therefore, in our case, it could be speculated that the downregulation of enzymes involved in carbon metabolism leading to perturbations in the cellular homeostasis, especially the energy production and this coupled with the downregulation of membrane transporters mentioned below could negatively impact the extrusion of intracellularly accumulated Phi. Further, this physiological stress could adversely impact the growth and virulence of *P. cinnamomi*.

##### 3.3.2.3 Increased abundance of putrescine biosynthetic enzymes

Arginase, an enzyme in the urea cycle responsible for L-ornithine synthesis (log_2_FC = 0.73), and ornithine decarboxylase (decarboxylation of L-ornithine; log_2_FC = 2.58) an enzyme involved in the biosynthesis of putrescine, a polyamine, were both upregulated in this study (Supplementary Table S1 and Figures 4, 5). Arginase activity has been shown to increase in *C. neoformans* lacking urease (Toplis et al., 2020) and the downregulation of urease (log_2_FC = −1.13) in this study could point to higher arginase activity, resulting in higher L-ornithine levels, increasing availability and downstream putrescine production by ornithine decarboxylase. The urea cycle is involved in the detoxification of ammonia generated by amino acid catabolism (Morris, 2002). Upregulation of several urea cycle enzymes (two argininosuccinate lyases, two carbamoyl phosphate synthase large-subunit proteins, and ornithine transcarbamylase) support the hypothesis that Phi exposure induces amino acid degradation for energy production. Putrescine (1,4-diaminobutane), a four-carbon diamine, is the first step in the synthesis of higher polyamines such as spermidine and spermine. Putrescine catabolism leads to the formation of gamma-aminobutyric acid (GABA), which can be converted into succinate through the GABA shunt. Succinate then enters the TCA cycle, linking polyamine metabolism to central energy production pathways.

Putrescine is reported to be involved in the regulation of nucleic acid and protein synthesis, and is thus essential for cell growth in both mammals and the fungus *Sclerotium rolfsii* (Shapira et al., 1989; Smith, 1990). Polyamines, including putrescine, are also implicated in the regulation of chromatin structure, membrane function, the control of transcription and translation, and oxidative stress tolerance (Solmi et al., 2023). Studies involving inhibitors of putrescine biosynthesis using α-difluoromethylornithine (DFMO), an irreversible inhibitor of ornithine decarboxylase, have shown significant inhibition of mycelial growth in several fungal phytopathogens—*Botrytis cinerea, Colletotrichum truncatum, Monilinia fructicola, Rhizoctonia solani*, and also in the oomycete *P. infestans* (Gamarnik et al., 1994; Rajam and Galston, 1985; Walters et al., 1995). This underscores the important role of putrescine and polyamine metabolism in fungal development.

Intracellular levels of putrescine and other polyamines are tightly regulated to maintain cellular homeostasis. Excessive accumulation of polyamines are known to be cytotoxic in *Neurospora crassa* due to alterations in the cytosolic K^+^ ion concentrations, and in the cyanobacterium *Anacystis nidulans* the toxicity was induced by ribosomal conjugation leading to dissociation of their subunits (Davis and Ristow, 1991; Guarino and Cohen, 1979). Due to their central role in cell proliferation and stress response, polyamine metabolism has emerged as a promising target for the development of putrescine analogs aimed at controlling commercially important fungal and oomycete phytopathogens (Garriz et al., 2003; Havis et al., 1994).

To the best of our knowledge, enzymes involved in putrescine catabolism to GABA as well as those responsible for spermidine and spermine biosynthesis, were not upregulated in this study. This absence raises questions regarding the intracellular accumulation of putrescine in the oomycete, *P. cinnamomi*. Is putrescine present at optimal levels that promote cell growth and stress adaptation, or does it accumulate to exert cytostatic or cytotoxic levels? Based on the observed growth inhibition of *Pc*GKB4 induced by Phi exposure, the latter scenario appears more likely. However, definitive conclusions require intracellular putrescine quantification in future studies.

##### 3.3.2.4 Mitochondrial translation and protein translocation machinery was scaled-up

Mitochondria are dynamic organelles which play a role not only in cellular respiration and energy production, but also in a wide range of essential cellular processes. Beyond ATP production, mitochondria are important for cell wall biogenesis and integrity due to their role in lipid homeostasis, calcium storage, iron–sulfur cluster assembly, cell signaling, and response to various stresses. They also contribute to detoxification of antifungal drugs, fungal quiescence and senescence, and fungal virulence, making them vital for cell proliferation and hyphal growth (Alves and Gourlay, 2024).

In fungi, mitochondrial genomes encode only eight genes, all of which are involved in ATP production through oxidative phosphorylation and cellular state regulation. The translation of these genes depends on a complex translation machinery that includes 78 mitoribosomal proteins, encoded in the nuclear genome. Meanwhile the mitochondrial 23S and 16S rRNAs are transcribed in the organelle itself. Mitoribosomal proteins are synthesized in the cytosol and imported into the mitochondria by translocases. Further, numerous assembly proteins, chaperones and peptidases are involved in the biogenesis and assembly of functional mitoribosomes (Bertgen et al., 2023; Itoh et al., 2020).

Phi-induced stress in *P. cinnamomi*, disrupts energy metabolism and redox homeostasis leading to oxidative stress. This was shown by significant alterations in the mitochondrial matrix and electron transport chain protein levels essential for mitochondrial function. In order to counter the stress, maintain the mitochondrial integrity and keep up with the energy requirements of the cell, significant upregulation of components of mitochondrial translation machinery involving mitoribosomes and accessory proteins (mitochondrial ribosomal protein (MRP); MRPL17, log_2_FC = 0.61; MRPL22, log_2_FC = 0.64; MRPL37, log_2_FC = 1.20; MRPL47, log_2_FC = 0.69; RSM27, log_2_FC = 0.65), ribosome recycling factor (log_2_FC = 0.74), translation initiation factor (log_2_FC = 0.68) and, mitochondrial protein translocases and chaperones (translocases of inner membrane (TIM), translocases of outer membrane (TOM); TIM9, log_2_FC = 0.62; TIM10, log_2_FC = 0.78; TIM16, log_2_FC = 0.69; TIM44 log_2_FC = 0.91; DnaK, log_2_FC = 0.85; mitochondrial BCS1-B, log_2_FC = 2.52) was necessitated (Supplementary Table S1 and Figures 4, 5). MRPL17, 22, 37 and 47 are constituents of large 39s ribosomal subunit, RSM27 is a part of the small subunit, and translation initiation factor IF2 is a GTP/GDP-binding protein that correctly positions initiator fMet-tRNA in the ribosomal P site and initiates translation, and translation elongation factor EF-Ts are critical for mitochondrial protein synthesis. Ribosome recycling factor is important for translational fidelity and disassembly of the post-termination complex. In addition to the above-mentioned import proteins multiple other translocases and chaperones—TIM8 and 13, TOM40 and 70, GrpE were upregulated (Supplementary Table S1, and Figure 5). The mitochondrial import machinery involves outer-, inter- and inner- membrane translocases, and chaperones that coordinate to transport the proteins synthesized in the cytosol. TOM40, a β-barrel protein, forms a translocation channel and is the vital component of the TOM complex and TOM70 is an outer membrane receptor facing the cytosol and with the coordinated action of GrpE-DnaK binds to preproteins for import (Fan and Young, 2011). The small TIM complexes—TIM8–TIM13 and TIM9–TIM10 present in the intermembrane space act in the transfer of protein precursors from the TOM to the inner membrane. Gene deletion studies showed TIM9–TIM10 and TOM40 to be essential for cell viability and growth (Baker et al., 2012; Wiedemann et al., 2004). Further, the upregulation of mitochondrial carrier family proteins, as discussed earlier, likely contributes to mitochondrial replication and protein synthesis (Palmieri, 2013). Our results are in contrast to Andronis et al. (2024) in which a downregulation of mitochondrial large subunit proteins was reported.

GTPase Era (log_2_FC = 4.33), the most upregulated protein, is essential for bacterial-type ribosome biogenesis such as mitochondrial ribosomes. Its deficiency impairs protein synthesis and cellular physiology, influencing growth and cell cycle regulation in both prokaryotes and eukaryotes (Gollop and March, 1991; Gruffaz and Smirnov, 2023).

Two ADP-ribosylation factor (ARF) family proteins (log_2_FC = 3.65 and log_2_FC = 3.00) were upregulated in the present study. While these small GTPases are primarily involved in vesicle assembly, cargo transport, and cytoskeletal reorganization, a prerequisite for cellular homeostasis and growth (Wang et al., 2020), Arf1 has been implicated in the modulation of mitochondrial morphology and homeostasis (Ackema et al., 2014), while *Fg*Arl1, an ADP-ribosylation factor-like small GTPase, is critical for the development and pathogenicity of the Fusarium head blight pathogen (Wang et al., 2020). Consistent with our study, King et al. (2010), also reported the upregulation of multiple *ARF* genes.

##### 3.3.2.5 Biosynthesis of secondary metabolites and intermediates/precursors involved in their biosynthesis was overrepresented in both the down- and up-regulated proteins

The biosynthesis of secondary metabolites (KEGG pathway ID: map01110) included upregulation of geranylgeranyl pyrophosphate synthetase (GGPS; log_2_FC = 1.19) (Supplementary Tables S1–S3). GGPS is an enzyme in the mevalonate/isoprenoid pathway responsible for the synthesis of an isoprenoid—geranylgeranyl pyrophosphate (GGPP). In *Phytophthora sojae* deletion of a GGPS-encoding gene affected the mycelial growth and morphology, as well as a reduction in the production of sporangia, oospores and virulence (Yang et al., 2021). In addition, GGPP is one of precursors involved in protein prenylation, a post translational modification of proteins. Protein prenylation is important for protein-protein interactions, protein trafficking and membrane localization of proteins (Sinensky, 2000). Further, the modification is critical in signal transduction (Ras superfamily proteins, G proteins), cytoskeletal organization and vesicular transport (Jung and Bachmann, 2023). In this study the upregulation of GGPS upon Phi-treatment could aid in the survival of *P. cinnamomi*. Multiple 12-oxophytodienoate reductases (log_2_FC = 1.05, 1.04, 0.86, **–**0.65, **–**0.75, **–**0.75) which catalyzes a key step in JA biosynthesis were differentially expressed. The importance of JA pathway in plant defense is well established and our previous research has also shown the involvement of this pathway in the avocado defense against *P. cinnamomi* during its transition from biotrophic to necrotrophic lifestyle (Van Den Berg et al., 2021). However, the biosynthesis and the physiological role of JA in *P. cinnamomi* or other oomycetes is not known to the best of our knowledge. Few reports are available on the physiological implications of intrinsic JA and their derivatives in bacteria and fungi. Intrinsic JA in the rice blast fungus *M. oryzae* was reported to be a morphogenetic signal that aids in the switch from the vegetative to the pathogenic phase. Deletion of the fungal 12-oxophytodienoic acid gene leading to reduction in JA levels resulted in prolonged germ tube growth, and affected the timely initiation and development of infection structures (Liu et al., 2021). In addition, *M. oryzae* was shown to employ hydroxylated-JA in host penetration and attenuation of host immunity (Patkar et al., 2015). The grapevine pathogen *Lasiodiplodia mediterranea* produces lasiojasmonate A, a JA-furanone ester, at late infection stages to facilitate fungal invasion through the induction of JA-mediated cell death response (Chini et al., 2018). In *Pseudomonas syringae* coronatine, a mimic of the plant JA conjugate (+)-7-iso-jasmonoyl-L-isoleucine, is a vital virulence factor that binds to the JA-receptor COI1 leading to suppression of the host salicylic acid defense through the activation of JA signaling, Further, coronative promotes bacterial entry into the host through the suppression of plant cell wall and stomatal defense responses (Geng et al., 2014). In the present context, down-regulation of three 12-oxophytodienoate reductases upon Phi treatment may impair the ability of *P. cinnamomi* to synthesize JA-like molecules, which are thought to play a role in modulating host defense or coordinating pathogen signaling. This disruption could weaken the pathogen's ability to suppress plant immune responses, reducing its virulence and ability to adapt to stress, and ultimately compromising survival and infection success. On the other hand, the observed upregulation of three 12-oxophytodienoate reductases, mentioned above, could be a ploy used by *P. cinnamomi* to enhance its chances at survival and infection. In future, determination of the levels of JA and/or its conjugates and functional studies will throw more light on their role in *P. cinnamomi*. Most other overrepresented proteins were found to be involved in fatty acid and amino acid metabolism, the TCA cycle, acetyl CoA metabolism, and biosynthesis of cofactors such as S-adenosylmethionine, CoA and heme, which could act as precursors/intermediates in the biosynthesis of various secondary metabolites. No prior studies have reported the induction of secondary metabolite biosynthesis in *P. cinnamomi* following *in vitro* Phi treatment. This warrants further investigation to elucidate the potential role of these metabolites under Phi-induced stress.

##### 3.3.2.6 Protein degradation machinery is differentially regulated

The ubiquitin-proteasome pathway plays a key role in the degradation of soluble, short-lived intracellular proteins involved in various regulatory cellular processes such as cell cycle control, gene expression, signal transduction, and metabolism. It also facilitates the targeted elimination of misfolded and damaged proteins (Staszczak, 2021), thereby maintaining protein quality and cellular homeostasis. This system is important under both normal as well as stress conditions, where its components are often differentially expressed (Andronis et al., 2024; Cao and Xue, 2021). *In vitro* Phi treatment of the *P. cinnamomi* significantly increased the abundance of several ubiquitin-proteasome subunit proteins (activator-subunit-1, beta-type-6, Cue, PI31; log_2_FC > 0.58; Supplementary Table S1, and Figure 4). In addition, multiple proteins/subunits/regulatory proteins involved in the ubiquitin-proteasome pathway were differentially expressed (16 upregulated and 14 downregulated; Supplementary Table S1, and Figure 5). Andronis et al. (2024), however, reported an enrichment of the proteasome complex proteins. Our findings suggest that Phi-induced stress triggers a complex regulation of the ubiquitin-proteosome system to maintain protein homeostasis and adapt to cellular stress.

### 3.4 Increased expression of serine proteases contributes to stress response

A significant increase in the abundance of five serine protease family members (log_2_FC = 2.06, 1.39, 1.29, 0.85, 0.65) was observed in *Pc*GKB4 following *in vitro* Phi treatment (Supplementary Table S1). This upregulation is crucial for the oomycete, as serine proteases are essential in protein turnover, signal peptide cleavage, protein maturation, nutrient uptake, cytochrome processing in mitochondria, and signal transduction. In addition to these fundamental functions, serine proteases have also been implicated in oxidative stress responses. In *Clonostachys rosea*, they contribute to stress tolerance by preventing protein aggregation and acting as molecular chaperones. They also facilitate autophagic recycling under nutrient-deficient conditions, a mechanism essential for survival in yeast (Muszewska et al., 2017; Zou et al., 2010). Significant increase in the abundance of serine proteases is consistent with the upregulation of proteolytic enzymes reported by Andronis et al. (2024).

### 3.5 Peroxisomal proteins involved in β-oxidation and detoxification of reactive species are upregulated

Peroxisomes are important organelles involved in lipid homeostasis, ROS detoxification, and secondary metabolite metabolism (Steinberg, 2016). In the present study, an increased abundance of enzymes involved in fatty acid catabolism was observed, including long-chain acyl-CoA synthetase (log_2_FC = 1.04), peroxisomal multifunctional enzyme type 2 (log_2_FC = 0.79), hydroxy methylglutaryl CoA-lyase (log_2_FC = 0.84), 3-ketoacyl-CoA-thiolase-A (log_2_FC = 1.39). These enzymes contribute to energy production and are accompanied by increased levels of ROS detoxification enzymes such as thioredoxin-dependent peroxide reductase (log_2_FC = 0.74), catalase (log_2_FC = 0.89), and superoxide dismutase (log_2_FC = 1.12), suggesting a coordinated response to the oxidative stress induced by the xenobiotic, Phi. Furthermore, bifunctional epoxide hydrolase 2 (log_2_FC = 2.10) involved in detoxication of highly reactive epoxides into less harmful diols, and various xenobiotics was significantly upregulated (Gautheron and Jéru, 2021). Epoxides are known to damage cellular macromolecules such as nucleic acid, lipids and proteins. This upregulation of protective enzymes indicates the intracellular induction of deleterious reactive species in response to Phi treatment. Additionally, peroxisomal membrane protein PEX16 (log_2_FC = 1.19) which is essential for peroxisome biogenesis, was also upregulated (Supplementary Tables S1, S3, and Figure 5), further highlighting an adaptive peroxisomal response.

### 3.6 Upregulation of multiple antioxidants/ROS scavengers indicates oxidative stress

The upregulation of a multitude of ROS scavengers or antioxidant proteins in response to Phi treatment indicates that *P. cinnamomi* is experiencing significant oxidative stress. Among these, catalase/peroxidase-HPI (log_2_FC = 2.76), superoxide dismutase (log_2_FC = 1.16), glutathione S-transferase (log_2_FC = 1.05) and glutathione transferase theta class (log_2_FC = 0.84), catalase (log_2_FC = 0.89), thioredoxin-dependent peroxide reductase (log_2_FC = 0.74), and thioredoxin-like protein (log_2_FC = 0.67), were all induced as part of stress-induced protective responses (Supplementary Table S1). Interestingly, some redundant antioxidant enzymes (glutathione peroxidase, log_2_FC = −0.70; thioredoxin, log_2_FC = −1.35; catalase, log_2_FC = −1.49) were downregulated—potentially to alleviate strain on the protein synthesis machinery and to maintain redox homeostasis. However, this downregulation could adversely impact *P. cinnamomi*'s oxidative stress response, aligning with the significant reduction in mycelial growth following Phi treatment. In *C. albicans*, catalase, glutathione and thioredoxin systems are activated in response to oxidative damage, with catalase acting as the first responder (Dantas Ada et al., 2015; Komalapriya et al., 2015).

Endogenous ROS such as hydrogen peroxide (H_2_O_2_), hydroxyl radical (HO·) and superoxide (${\text{O}}_{2}^{-}$) are inevitable byproducts of cellular respiration, primarily generated in mitochondria. While low levels of ROS serve as secondary messengers that support fungal growth and development, excessive accumulation leads to cytotoxicity by damaging cellular macromolecules. Superoxide dismutases (SODs) detoxify HO· and ${\text{O}}_{2}^{-}$ by converting them into H_2_O_2_, which is further neutralized into H_2_O and dioxygen by catalases and peroxidases (Yaakoub et al., 2022).

Glutathione, a tripeptide composed of Glu-Cys-Gly, functions as a crucial cofactor for antioxidant enzymes like glutathione peroxidases and glutathione S-transferases. It also directly scavenges the free radicals through redox reactions. In fungal systems deletion of the glutathione genes negatively affected growth and fitness (Wangsanut and Pongpom, 2022). Similarly, the antioxidant thioredoxin system comprised of thioredoxin, thioredoxin reductase, and peroxiredoxin plays a key role in oxidative stress management. In *Aspergillus nidulans*, deletion of these components resulted in reduced mycelial growth due to the inability to manage the oxidative stress (Thon et al., 2007). Furthermore, studies in *Cryptococcus neoformans* using thioredoxin reductase promoter replacement showed the enzyme's essential role in fungal cell viability, highlighting it as a potential antifungal target against this opportunistic pathogen (Missall and Lodge, 2005).

α-1,3-mannosyltransferase (log_2_FC = 3.72), in the rice blast fungus *M. oryzae* was shown to be important in vegetative as well as invasive growth, cell wall integrity, conidia formation, cellular localization and secretion of various proteins, suppression of ROS production and virulence (Chen et al., 2014). Its upregulation during Phi exposure in *P. cinnamomi* may be to repress oxidative stress and aid in protein localization through N-glycosylation.

Pyridoxal biosynthesis lyase, essential for vitamin B6 biosynthesis, was significantly upregulated (log_2_FC = 1.95), consistent with findings from a related study (King et al., 2010). Vitamin B6, in addition to its role in the metabolism of carbohydrates, fats and proteins, also acts as an antioxidant (Mooney et al., 2009), potentially contributing to the oxidative stress response in *P. cinnamomi*.

### 3.7 Downregulation of membrane and transmembrane proteins may impact nutrient and Phi flux

We found membrane and transmembrane proteins to be predominantly downregulated (Supplementary Table S2); including five ABC superfamily proteins (log_2_FC between −0.13 to −0.69 and one single MFS (log_2_FC = −0.81) (Supplementary Table S1). ABC and MFS transporters are vital for nutrient uptake, molecular transport, signal transduction, stress response, maintaining membrane integrity, and for dealing with xenobiotics (Drew et al., 2021; Viglas and Olejnikova, 2021). Their downregulation in response to Phi may impact both nutrient availability and Phi efflux, potentially contributing to Phi accumulation and toxicity (Figure 5). This dual role of transporters—as both entry and exit routes for Phi—underscores their contradictory effect under xenobiotic stress. Future studies quantifying intra- and extracellular Phi could clarify its transport dynamics and correlation with growth inhibition. On the contrary Phi sensitive *P. cinnamomi* isolate showed enrichment of transmembrane transport proteins post Phi treatment (Andronis et al., 2024).

## 4 Conclusion

To the best of our knowledge, this is the first study reporting on Phi-induced upregulation of mitochondrial protein translocases, putrescine biosynthesis, peroxisomal proteins, and alternative energy generation through the activation of β-oxidation and amino acid catabolism. We have also shown the downregulation of enzymes associated with pyruvate metabolism and differential expression of proteins involved in the biosynthesis of secondary metabolites and intermediates/precursors involved in their biosynthesis, not reported earlier. Common responses with the previous study (Andronis et al., 2024) included suppression of the glycolytic pathway, mitochondrial electron transport chain components, and upregulation of antioxidants, RNA processing proteins and proteases. The expression trends of mitochondrial large ribosome subunits and transmembrane proteins were found to be opposite to the previous report (Andronis et al., 2024). In contrast to Andronis et al. (2024) the current study found no changes in the abundance levels of proteins involved in inositol phosphorylation, biosynthesis, or signaling proteins, protein phosphorylation, DNA damage response, regulators of cell growth and metabolism.

Phi inhibits *P. cinnamomi* growth predominantly by reducing the abundance of key oxidoreductases involved in bioenergetics, redox homeostasis and other metabolic processes. Disruptions to mitochondrial machinery leading to changes in energy metabolism, oxidative stress, and membrane/transmembrane proteins are the major driving factors that affect the cellular functioning and growth. Additionally, increase in putrescine biosynthesis, a decline in the abundance of major membrane transporters, pyruvate metabolism, and changes in cellular homeostasis may also contribute to the observed inhibition of growth. The potential role of secondary metabolites in Phi-induced stress needs further investigation. Upregulation of mitochondrial translation and translocation machinery, peroxisomal proteins, alternate energy-generating pathways, and antioxidants could be a survival strategy employed by *P. cinnamomi*, through the partial restoration of cellular homeostasis. The present study contributes to our still limited understanding of the underlying basis of the direct molecular effects of Phi on oomycete pathogens. To build on these findings, multi-omics approaches are essential to identify additional molecular targets and validate their function. A deeper understanding into Phi's mode of action will aid in the development of improved or novel chemical intervention strategies to manage oomycete pathogens.

## 5 Future perspectives

To achieve a more comprehensive understanding of the direct inhibitory mechanism of Phi on *P. cinnamomi* the following key directions are recommended:

1. Establish standardized protocols for evaluating Phi sensitivity of *P. cinnamomi* isolates. This includes standardizing culture media composition, Pi (phosphate) concentration, incubation conditions (time and temperature), Phi source and concentration (EC_50_), and sensitivity assessment methods (preferably optical density-based growth measurements). This is to ensure reproducibility and comparability across studies conducted in different laboratories.
2. Screen a diverse set of *P. cinnamomi* isolates with varying Phi sensitivities, collected from distinct geographical locations with different histories of Phi usage. Apply a multi-omics approach (genomics, transcriptomics, proteomics, metabolite profiling) to identify key molecular players involved in Phi response.
3. Conduct temporal studies to correlate the omics data with the observed changes in vegetative and reproductive morphology.
4. Monitor the temporal dynamics of Pi:Phi concentrations: both intracellularly and in culture filtrates. This will be crucial for correlating the Pi:Phi flux with observed molecular and phenotypic changes as a result of Phi.
5. Investigate the global phosphorylation status of proteins in response to Phi to uncover potential disruptions in key signaling and regulatory pathways.
6. Functionally characterize candidate genes and proteins identified through omics analyses to elucidate their roles in Phi-mediated inhibition. This will help uncover the molecular basis of Phi's direct mode of action and may reveal potential targets for improved disease control strategies.
7. Leverage the key molecular targets/pathways identified by the multi-omics approach followed by functional studies to develop new chemicals to mitigate the chemical resistance. In cases where chemicals targeting one or more key molecular targets of interest are already available in the market (against other pathogens) they can be screened for their efficacy and adapted into the current system, if applicable.

## Data Availability

The original contributions presented in the study are publicly available. This data can be found in here: https://www.ebi.ac.uk/pride/archive/, PXD063718.

## Supplementary material

Supplementary Figure S1 Heatmap displaying the expression of significantly differentially expressed proteins in *Phytophthora cinnamomi* isolate GKB4 (untreated control vs. Phi-treated samples).
