# Supplementary figures and images for: Phosphite inhibits Phytophthora cinnamomi by downregulating oxidoreductases and disrupting energy metabolism
Source: Front Microbiol. 2025 Aug 25;16:1632726. doi: 10.3389/fmicb.2025.1632726 (PMC12414984; doi:10.3389/fmicb.2025.1632726)

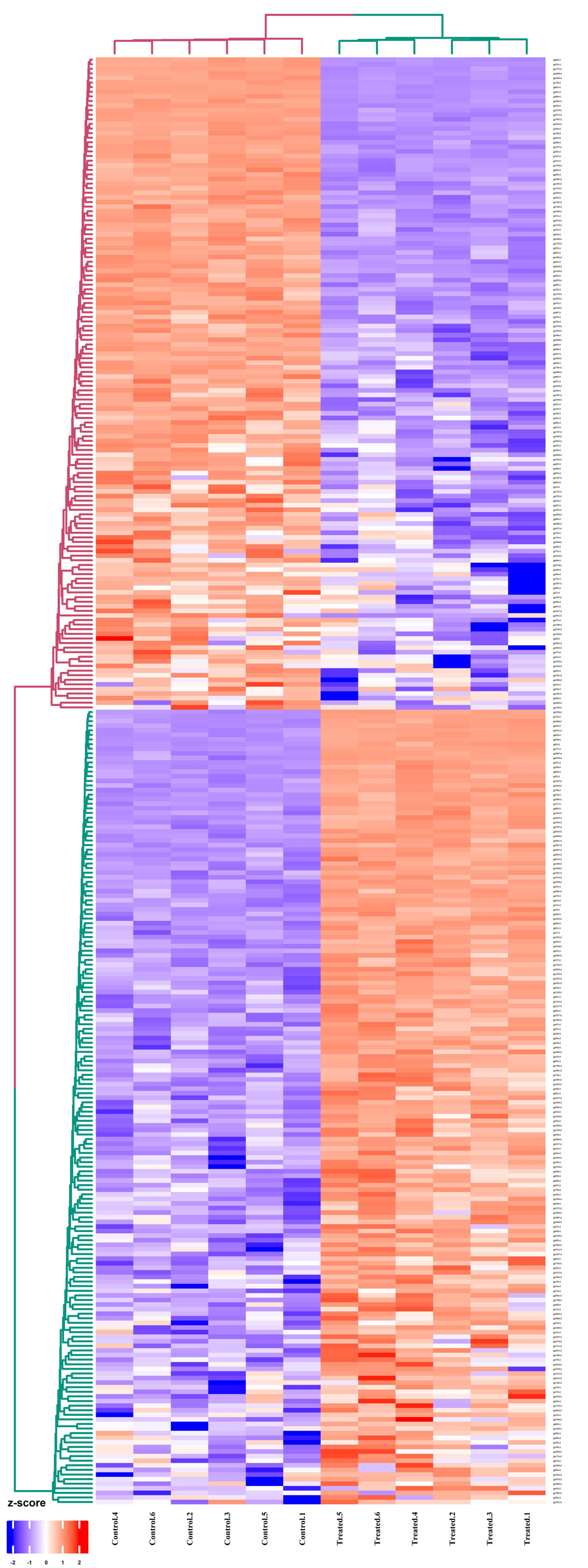

Supplement: Supplementary Figure S1 — Heatmap displaying the expression of significantly differentially expressed proteins in Phytophthora cinnamomi isolate GKB4 (untreated control vs. Phi-treated samples). [file Image_1.jpeg]
