# Supplementary material for: Phosphite inhibits Phytophthora cinnamomi by downregulating oxidoreductases and disrupting energy metabolism
Source: Front Microbiol. 2025 Aug 25;16:1632726. doi: 10.3389/fmicb.2025.1632726 (PMC12414984; doi:10.3389/fmicb.2025.1632726)
Supplement: Supplementary file 2 [file Table_1.docx]

**Supplementary Table S1.** The differential protein abundance list. A Student’s two-sample *t*-test was performed in Perseus v2.0.11 (Tyanova et al., 2016) with phosphite-treated as first group and untreated control as the second group, S0=0.1, 250 randomizations, permutation-based *q*-value cut-off of <0.05 and the differential abundance list was generated by filtering for *t*-test significant values. log_2_FC: log_2_ fold-change.

| **Sl. No.** | **Protein Groups** | **log_2_FC** | ***q*-value** |  |
| --- | --- | --- | --- | --- |
| **Downregulated List** | | | | |
|  | jg1951.t1_Ubiquinone-biosynthesis-protein-COQ7 | -5.02397 | 0 |  |
|  | jg5515.t1_Alternative-oxidase | -4.7438 | 0 |  |
|  | jg7751.t1_pyruvate-phosphate-dikinase; jg7752.t1_pyruvate-phosphate-dikinase; jg7753.t1_pyruvate-phosphate-dikinase | -3.99625 | 0.001169 |  |
|  | jg5513.t1_Alternative-oxidase | -3.03865 | 0.000141 |  |
|  | jg5498.t1_Membrane-associated-eicosanoid/glutathione-metabolism-like-domain | -2.97442 | 0 |  |
|  | jg13772.t1_Cysteine-desulfurase | -2.73097 | 0 |  |
|  | jg8624.t1_D-isomer-specific-2-hydroxyacid-dehydrogenase | -2.45735 | 0 |  |
|  | jg1282.t1_Transmembrane-prolyl-4-hydroxylase | -2.41298 | 0 |  |
|  | jg16884.t1_Ferric-reductase; jg16884.t2_Ferric-reductase | -2.38947 | 0 |  |
|  | jg14379.t1_homoserine-O-acetyltransferase | -2.35242 | 0 |  |
|  | jg14375.t1_Glucan 1,3-beta-glucosidase | -2.30929 | 0 |  |
|  | jg3968.t1_isocitrate-dehydrogenase-[NADP]-mitochondrial | -2.27488 | 0 |  |
|  | jg12749.t1_coproporphyrinogen-III-oxidase | -2.20801 | 0 |  |
|  | jg11357.t1_ Ubiquinone-biosynthesis-protein-COQ7 | -2.09316 | 0 |  |
|  | jg6992.t1_L-amino-acid-oxidase | -2.05288 | 0 |  |
|  | jg18399.t1_Fructose-16-bisphosphatase | -2.04304 | 0.002047 |  |
|  | jg7167.t1_indigoidine-synthase-A-family-protein-putative | -1.94709 | 0 |  |
|  | jg14691.t1_fatty-acid-desaturase-family-Zn-binding-site | -1.91371 | 0 |  |
|  | jg5497.t1_ Membrane-associated-proteins in eicosanoid/glutathione-metabolism-like-domain (MAPEG) | -1.90216 | 0.00106 |  |
|  | jg9882.t1_Apoptosis-inducing-factor-B; jg9883.t1_Apoptosis-inducing-factor-B | -1.77929 | 2.61E-05 |  |
|  | jg11767.t1_Clavaminate-synthase-like-protein | -1.75445 | 0.000216 |  |
|  | jg1621.t1_Mitochondrial-Carrier-(MC)-Family | -1.7219 | 0 |  |
|  | jg14394.t1_PhosphoLipase-D | -1.70062 | 0.007739 |  |
|  | jg2100.t1_ Membrane-associated-proteins in eicosanoid/glutathione-metabolism-like-domain (MAPEG); jg2101.t1_ Membrane-associated-proteins in eicosanoid/glutathione-metabolism-like-domain (MAPEG); jg5495.t1_Membrane-associated-proteins in eicosanoid/glutathione-metabolism-like-domain (MAPEG); jg5496.t1_ Membrane-associated-proteins in eicosanoid/glutathione-metabolism-like-domain (MAPEG) | -1.66915 | 3.29E-05 |  |
|  | jg3336.t1_MICAL-like-protein | -1.63004 | 0.004568 |  |
|  | jg6505.t1_glyceraldehyde-3-phosphate-dehydrogenase-type-I | -1.58625 | 0 |  |
|  | jg13240.t1_Histone-lysine-N-methyltransferase-EZH2; jg13241.t1_Histone-lysine-N-methyltransferase-EZH2; jg13252.t1_Histone-lysine-N-methyltransferase-EZH2; jg13253.t1_Histone-lysine-N-methyltransferase-EZH2 | -1.57819 | 0 |  |
|  | jg5450.t1_aquaporin-putative; jg5451.t1_aquaporin-putative | -1.57474 | 0.001123 |  |
|  | jg5514.t1_Alanine--tRNA-ligase | -1.57064 | 0 |  |
|  | jg18638.t1_Catalase | -1.49831 | 0 |  |
|  | jg7760.t1_betaine-aldehyde-dehydrogenase-2-mitochondrial | -1.48361 | 0 |  |
|  | jg18401.t1_Fructose-16-bisphosphatase | -1.4542 | 0 |  |
|  | jg8633.t1_D-isomer-specific-2-hydroxyacid-dehydrogenase-putative | -1.44875 | 0 |  |
|  | jg8092.t1_Mannitol-dehydrogenase | -1.43548 | 0 |  |
|  | jg16774.t1_thioredoxin-putative; jg16775.t1_thioredoxin-putative | -1.34937 | 0 |  |
|  | jg7621.t1_Alanine-aminotransferase-2 | -1.30605 | 0 |  |
|  | jg18400.t1_Fructose-16-bisphosphatase | -1.28203 | 0 |  |
|  | jg5502.t1_Glutathione-S-transferase | -1.24478 | 0 |  |
|  | jg8349.t1_zinc-dependent-alcohol-dehydrogenase; jg8351.t1_zinc-dependent-alcohol-dehydrogenase; jg8353.t1_zinc-dependent-alcohol-dehydrogenase | -1.2158 | 0 |  |
|  | jg14507.t1_hypothetical-protein-PHYSODRAFT_328276 | -1.19176 | 0.009483 |  |
|  | jg10698.t1_Ammonium-Transporter-(Amt)-Family | -1.19146 | 0 |  |
|  | jg14353.t1_Deoxyribose-phosphate-aldolase | -1.1911 | 0 |  |
|  | jg4414.t1_Tyrosine--tRNA-ligase; jg4415.t1_Tyrosine--tRNA-ligase | -1.18571 | 0.004045 |  |
|  | jg17987.t1_Ca2-:Cation-Antiporter-(CaCA)-Family | -1.16317 | 0 |  |
|  | jg1521.t1_fumarate-reductase-flavoprotein-subunit-putative | -1.16031 | 0 |  |
|  | jg10684.t1_Ammonium-Transporter-(Amt)-Family | -1.14096 | 0.000117 |  |
|  | jg14699.t1_Double-strand-break-repair-protein-rad21 | -1.13699 | 0 |  |
|  | jg16690.t1_Urease-alpha-subunit | -1.13445 | 0 |  |
|  | jg8499.t1_Mitochondrial-processing-peptidase-subunit-beta | -1.13345 | 0 |  |
|  | jg18579.t1_Phosphate-acetyltransferase | -1.13157 | 0 |  |
|  | jg17995.t1_Kelch-repeat-containing-proteins | -1.1264 | 3.08E-05 |  |
|  | jg13847.t1_Isoflavone-reductase-P3; jg1553.t1_Isoflavone-reductase-P3; jg1554.t1_Isoflavone-reductase-P3 | -1.10209 | 0.011664 |  |
|  | jg16748.t1_Cytochrome-c-oxidase-assembly-protein-COX15 | -1.05154 | 0.015131 |  |
|  | jg5983.t1_2-aminoethanethiol-dioxygenase | -1.03777 | 0.001534 |  |
|  | jg1454.t1_dna-topoisomerase-i | -1.03418 | 0 |  |
|  | jg4732.t1_Malate-dehydrogenase | -0.99378 | 0 |  |
|  | jg10012.t1_protein-disulfide-isomerase-domain | -0.99356 | 6.31E-05 |  |
|  | jg19530.t1_Nucleotide-binding-protein | -0.98247 | 0.000119 |  |
|  | jg11610.t1_START-like-domain; jg11610.t2_START-like-domain | -0.97837 | 0.002839 |  |
|  | jg11842.t1_triose-phosphate-isomerase | -0.97421 | 0.001215 |  |
|  | jg4432.t1_Mitochondrial-Carrier-(MC)-Family | -0.96515 | 0 |  |
|  | jg8350.t1_zinc-dependent-alcohol-dehydrogenase; jg8352.t1_zinc-dependent-alcohol-dehydrogenase | -0.96113 | 0 |  |
|  | jg239.t1_AGC-protein-kinase | -0.95762 | 0.001138 |  |
|  | jg18173.t1_AP-3-complex-subunit-delta-putative | -0.95709 | 0.000505 |  |
|  | jg3807.t1_UTP-glucose-1-phosphate-uridylyltransferase | -0.94763 | 0 |  |
|  | jg6933.t1_sialin-like-protein | -0.94239 | 0.001556 |  |
|  | jg4772.t1_START-like-domain | -0.93983 | 0.018751 |  |
|  | jg15320.t1_Callose-synthase | -0.92394 | 0 |  |
|  | jg7731.t1_Enolase | -0.92347 | 0.004487 |  |
|  | jg4931.t1_Delta(5)-fatty-acid-desaturase | -0.90787 | 3.13E-05 |  |
|  | jg12004.t1_fructose-bisphosphate-aldolase-class-II; jg16527.t1_fructose-bisphosphate-aldolase-class-II | -0.89936 | 0.016928 |  |
|  | jg9995.t1_Alcohol-dehydrogenase | -0.89868 | 0.007441 |  |
|  | jg8769.t1_amine-oxidase-like-protein | -0.86498 | 0 |  |
|  | jg15841.t1_Golgi-apparatus-membrane-protein-TVP15 | -0.86317 | 0.017174 |  |
|  | jg3583.t1_6-phosphofructo-2-kinase/fructose-26-biphosphatase-putative | -0.85985 | 0.009247 |  |
|  | jg17671.t1_phenol-acid-carboxylase-putative | -0.85901 | 0.000865 |  |
|  | jg11899.t1_family-31-glycoside-hydrolase | -0.83639 | 0 |  |
|  | jg19902.t1_NADP-dependent-malic-enzyme | -0.82835 | 0 |  |
|  | jg3629.t1_urate-oxidase; jg3630.t1_urate-oxidase | -0.81889 | 0 |  |
|  | jg17499.t1_Major-Facilitator-Superfamily-(MFS) | -0.81404 | 0 |  |
|  | jg15701.t1_NAD-dependent-glycerol-3-phosphate-dehydrogenase | -0.81236 | 0.000381 |  |
|  | jg12759.t2_E3-ubiquitin-ligase-complex-SCF-subunit-scon-3 | -0.80397 | 3.27E-05 |  |
|  | jg18468.t1_lipase-putative | -0.78772 | 0.000116 |  |
|  | jg2008.t1_Glycerol-3-phosphate-O-acyltransferase | -0.78248 | 0.000232 |  |
|  | jg5622.t1_High-affinity-copper-uptake-protein-1 | -0.77372 | 0.015939 |  |
|  | jg17251.t1_Crinkler-(CRN)-family-protein; jg17251.t2_Crinkler-(CRN)-family-protein | -0.76658 | 0.005219 |  |
|  | jg13667.t1_Serine/threonine-protein-kinase | -0.76085 | 0.00014 |  |
|  | jg2570.t1_12-oxophytodienoate-reductase; jg2572.t1_12-oxophytodienoate-reductase | -0.75488 | 0 |  |
|  | jg19754.t1_Membrane-associated-eicosanoid/glutathione-metabolism-(MAPEG)-protein | -0.75363 | 0.001066 |  |
|  | jg8348.t1_zinc-dependent-alcohol-dehydrogenase | -0.75271 | 0.048988 |  |
|  | jg4687.t1_Cytosine-deaminase | -0.74434 | 0.000121 |  |
|  | jg13436.t1_Armadillo-type-fold | -0.74262 | 0.047094 |  |
|  | jg10020.t1_rap-ran-gtpase-activating-protein | -0.73722 | 0.039631 |  |
|  | jg4948.t1_conserved-hypothetical-protein | -0.73323 | 2.60E-05 |  |
|  | jg4180.t1_Uncharacterized-conserved-protein | -0.72328 | 0.040759 |  |
|  | jg15512.t1_ethanolamine-kinase-A | -0.71785 | 0.003927 |  |
|  | jg8640.t1_er-lumen-protein-retaining-receptor | -0.71592 | 0.00601 |  |
|  | jg206.t1_cell-5A-endo-14-betaglucanase | -0.70975 | 0.022619 |  |
|  | jg3862.t1_Estradiol-17-beta-dehydrogenase | -0.70401 | 3.31E-05 |  |
|  | jg12766.t1_Glutathione-peroxidase | -0.70215 | 0 |  |
|  | jg2671.t1_zinc-finger-CDGSH-domain-containing-protein-1 | -0.69852 | 8.36E-05 |  |
|  | jg9996.t1_alcohol-dehydrogenase-putative | -0.69846 | 0.028094 |  |
|  | jg17461.t1_S-adenosyl-L-methionine-dependent-methyltransferase | -0.69478 | 0 |  |
|  | jg17136.t1_V-type-H(+)-translocating-pyrophosphatase | -0.69264 | 0 |  |
|  | jg5510.t1_ABC-transporter-like-protein | -0.69154 | 0 |  |
|  | jg15044.t1_phosphoglycerate-kinase-1 | -0.6784 | 0 |  |
|  | jg2697.t1_Carbohydrate-binding-protein | -0.67723 | 0.002075 |  |
|  | jg2853.t1_Endosomal-protein-P24B | -0.67544 | 0.039752 |  |
|  | jg8570.t1_Ubiquitin-specific-protease | -0.67177 | 0.000375 |  |
|  | jg1959.t1_Glucan-1 | -0.66824 | 0.000153 |  |
|  | jg12785.t1_alcohol-dehydrogenase-putative | -0.66171 | 0.036262 |  |
|  | jg11276.t1_myosin-like-protein | -0.66092 | 0 |  |
|  | jg7626.t1_pyrophosphate-dependent-phosphofructose-kinase | -0.66022 | 0 |  |
|  | jg14817.t1_2-oxo-acid-dehydrogenase-acyltransferase-catalytic-domain-protein | -0.65473 | 0.011175 |  |
|  | jg11086.t2_Mannose-1-phosphate-guanyltransferase-alpha | -0.65367 | 0.043441 |  |
|  | jg5279.t1_cellulose-synthase-4 | -0.65307 | 0.000118 |  |
|  | jg8626.t1_D-lactate-dehydrogenase | -0.65069 | 8.05E-05 |  |
|  | jg9194.t1_Endomembrane-protein-70 | -0.64871 | 0.046514 |  |
|  | jg15330.t1_12-oxophytodienoate-reductase-putative | -0.64669 | 2.85E-05 |  |
|  | jg13355.t1_transcription-initiation-factor-related-family-protein | -0.64367 | 0.039969 |  |
|  | jg19196.t1_Myosin-protein | -0.63467 | 3.01E-05 |  |
|  | jg9668.t1_Bacterial-PH-like-domain | -0.63127 | 0 |  |
|  | jg18770.t1_acyltransferase-ws-dgat-mgat-family-protein | -0.63065 | 0 |  |
|  | jg7534.t1_Quinone-oxidoreductase-protein | -0.62889 | 0 |  |
|  | jg113.t1_Lysophospholipid-acyltransferase | -0.62773 | 0.004759 |  |
|  | jg4773.t1_putative-isomerase | -0.62377 | 0.006193 |  |
|  | jg7466.t2_malate-synthase-A | -0.62037 | 0 |  |
|  | jg8623.t1_D-isomer-specific-2-hydroxyacid-dehydrogenase | -0.6202 | 0 |  |
|  | jg14167.t1_Ubiquitin-conjugation-factor-E4 | -0.61803 | 0.002838 |  |
|  | jg11919.t1_3-oxo-5-alpha-steroid-4-dehydrogenase-putative; jg496.t1_3-oxo-5-alpha-steroid-4-dehydrogenase-putative | -0.61481 | 3.32E-05 |  |
|  | jg1479.t1_Crinkler-(CRN)-family-protein | -0.61018 | 0.017391 |  |
|  | jg5126.t1_hypothetical-protein-PC110_g10328 | -0.60989 | 0.000117 |  |
|  | jg19508.t1_NmrA-like-family-protein | -0.60736 | 0.000863 |  |
|  | jg19729.t1_phytanoyl-CoA-dioxygenase-domain-containing-protein-putative | -0.60372 | 0.001292 |  |
|  | jg19529.t1_Nucleotide-binding-protein | -0.60155 | 0 |  |
|  | jg2009.t1_Acetyl-CoA-carboxylase | -0.59621 | 0 |  |
|  | jg17931.t2_cytoplasmic-dynein-1-heavy-chain-1 | -0.59457 | 0 |  |
|  | jg11528.t1_sepiapterin-reductase | -0.59222 | 0.003621 |  |
|  | jg3783.t1_Uncharacterized-conserved-protein | -0.59055 | 0 |  |
|  | jg3253.t1_AGC/PKG-protein-kinase; jg9225.t1_AGC/PKG-protein-kinase | -0.58989 | 0.000115 |  |
|  | jg14706.t1_saccharopine-dehydrogenase | -0.58097 | 0.007055 |  |
|  | jg19195.t1_hypothetical-protein-PHYSODRAFT_474772 | -0.58087 | 0 |  |
|  | jg5878.t1_Putative-aconitate-hydratase | -0.57481 | 0.000119 |  |
|  | jg2953.t1_NAD-dependent-epimerase/dehydratase | -0.56667 | 0.000231 |  |
|  | jg11275.t1_pyruvate-carboxylase | -0.56531 | 0 |  |
|  | jg8631.t1_D-isomer-specific-2-hydroxyacid-dehydrogenase; jg8632.t1_D-isomer-specific-2-hydroxyacid-dehydrogenase | -0.56389 | 0.000638 |  |
|  | jg15062.t1_Pyruvate-kinase | -0.56283 | 0 |  |
|  | jg16410.t1_TKL-protein-kinase; jg16410.t2_TKL-protein-kinase | -0.55832 | 0 |  |
|  | jg4436.t1_Bifunctional-aspartokinase/homoserine-dehydrogenase; jg4436.t2_Bifunctional-aspartokinase/homoserine-dehydrogenase | -0.55802 | 0.002371 |  |
|  | jg15407.t1_Brefeldin-A-inhibited-guanine-nucleotide-exchange-protein; jg15407.t4_Brefeldin-A-inhibited-guanine-nucleotide-exchange-protein | -0.55752 | 0.013032 |  |
|  | jg14708.t1_Vacuolar-protein-sorting-associated-protein-13B | -0.55739 | 3.14E-05 |  |
|  | jg12783.t1_alcohol-dehydrogenase-putative; jg12784.t1_alcohol-dehydrogenase-putative; jg12788.t1_alcohol-dehydrogenase-putative | -0.55433 | 0.001262 |  |
|  | jg19705.t1_Short-chain-dehydrogenase | -0.55383 | 0.000366 |  |
|  | jg16344.t1_aldehyde-dehydrogenase-mitochondrial-precursor | -0.55334 | 3.28E-05 |  |
|  | jg7349.t1_regulator-of-chromosome-condensation-(RCC1)-like-protein | -0.55219 | 0.033146 |  |
|  | jg17838.t1_glyceraldehyde-3-phosphate-dehydrogenase | -0.54816 | 0 |  |
|  | jg18628.t1_ABC-transporter-A-family-member-1 | -0.54762 | 0.002082 |  |
|  | jg8373.t1_Arrestin-domain-containing-protein-A | -0.54623 | 0.002184 |  |
|  | jg9980.t1_NEK-protein-kinase | -0.54144 | 0.049775 |  |
|  | jg12295.t1_TKL-protein-kinase | -0.53523 | 0.004572 |  |
|  | jg12750.t1_Coproporphyrinogen-III-oxidase | -0.534 | 0 |  |
|  | jg8648.t1_Proteasome-subunit-beta-type-5 | -0.53274 | 0.000114 |  |
|  | jg18979.t1_membrane-protein; jg18979.t2_membrane-protein | -0.52946 | 0.00303 |  |
|  | jg581.t1_aspartate-tRNA(Asn)-ligase | -0.52615 | 0 |  |
|  | jg17698.t1_PH-domain-like-protein | -0.52087 | 0.000249 |  |
|  | jg1754.t1_tyrosine-biosynthesis-bifunctional-enzyme | -0.51335 | 0 |  |
|  | jg19731.t2_putative-glutamate-carboxypeptidase | -0.5098 | 0.012326 |  |
|  | jg19900.t1_Mitochondrial-Carrier-(MC)-Family | -0.49593 | 2.73E-05 |  |
|  | jg5097.t1_cytochrome-c-oxidase-subunit-IV | -0.49575 | 0 |  |
|  | jg19645.t1_cytochrome-c | -0.49175 | 0.000373 |  |
|  | jg3655.t1_CCR4-NOT-transcription-complex-subunit-1 | -0.48569 | 0.030707 |  |
|  | jg430.t1_protein-phosphatase-1-regulatory-subunit-putative | -0.48449 | 0.020506 |  |
|  | jg1995.t1_ATP-citrate-synthase | -0.4827 | 0 |  |
|  | jg5252.t1_cellulose-synthase-3 | -0.48166 | 0 |  |
|  | jg5139.t1_glutathione-peroxidase | -0.48157 | 7.91E-05 |  |
|  | jg11256.t1_hypothetical-protein-PPTG_13252 | -0.4815 | 0.000974 |  |
|  | jg3998.t1_P-type-ATPase-(P-ATPase)-Superfamily | -0.48102 | 0.000538 |  |
|  | jg16729.t1_hypothetical-protein-PHYSODRAFT_295929 | -0.48078 | 0.006221 |  |
|  | jg15798.t1_NAD(P)H:quinone-oxidoreductase-type-IV; jg15805.t1_NAD(P)H:quinone-oxidoreductase-type-IV | -0.47867 | 0 |  |
|  | jg5058.t1_nicotinate-nucleotide-diphosphorylase-(carboxylating) | -0.47805 | 0 |  |
|  | jg10811.t1_Aldo/keto-reductase-family | -0.47434 | 2.65E-05 |  |
|  | jg10045.t1_histone-H2A.X; jg10046.t1_histone-H2A | -0.47416 | 0.005326 |  |
|  | jg12661.t1_2OG-Fe(II)-oxygenase-superfamily-protein | -0.4731 | 0.019169 |  |
|  | jg7637.t1_Serine/threonine-protein-phosphatase-5 | -0.47237 | 0.024108 |  |
|  | jg4483.t1_basic-elicitin; jg4484.t1_basic-elicitin | -0.47112 | 0.000508 |  |
|  | jg5542.t1_Dolichol-phosphate-mannosyltransferase | -0.46818 | 0.000692 |  |
|  | jg6967.t1_Small-nuclear-ribonucleoprotein-associated-protein | -0.46603 | 0.001555 |  |
|  | jg7456.t1_glycoside-hydrolase-putative | -0.46446 | 0.020026 |  |
|  | jg16376.t1_dipeptidyl-peptidase-putative | -0.46277 | 0 |  |
|  | jg19829.t1_short-chain-dehydrogenase-putative | -0.46189 | 0.008166 |  |
|  | jg12761.t1_E3-ubiquitin-ligase-complex-SCF-subunit-scon-3 | -0.46083 | 0 |  |
|  | jg957.t1_alcohol-dehydrogenase-putative | -0.45776 | 0 |  |
|  | jg9247.t1_predicted-protein | -0.4559 | 0 |  |
|  | jg16753.t2_plasma-membrane-proton-efflux-P-type-ATPase | -0.45441 | 0.000145 |  |
|  | jg14696.t1_asparagine-synthase-(glutamine-hydrolyzing) | -0.44989 | 0 |  |
|  | jg13517.t1_Glutathione-S-transferase-omega-protein | -0.44311 | 0.000689 |  |
|  | jg8255.t1_Ser-Thr-rich-glycosyl-phosphatidyl-inositol-anchored-membrane-family | -0.44236 | 0.003363 |  |
|  | jg12067.t1_hypothetical-protein-PHYSODRAFT_522260 | -0.44213 | 0.000816 |  |
|  | jg6090.t1_WD40-repeat-stress-protein/actin-interacting-protein | -0.44068 | 0 |  |
|  | jg17089.t1_Choline/Carnitine-O-acyltransferase | -0.43735 | 0 |  |
|  | jg13571.t1_Glutathione-S-transferase | -0.43396 | 6.17E-05 |  |
|  | jg8807.t1_Dihydrolipoyllysine-residue-acetyltransferase-component-of-pyruvate-dehydrogenase-complex | -0.43306 | 0 |  |
|  | jg7065.t1_dihydroflavonol-4-reductase-putative; jg7065.t2_dihydroflavonol-4-reductase-putative; jg7065.t3_dihydroflavonol-4-reductase-putative; jg7065.t4_dihydroflavonol-4-reductase-putative | -0.43266 | 0.002044 |  |
|  | jg10509.t1_outer-envelope-pore-protein-16-chloroplastic-mitochondrial-like | -0.43132 | 0.000862 |  |
|  | jg6017.t1_Alcohol-dehydrogenase-2 | -0.42793 | 0.022922 |  |
|  | jg13142.t1_phosphoenolpyruvate-carboxykinase-(ATP); jg9100.t1_phosphoenolpyruvate-carboxykinase-(ATP) | -0.42402 | 0 |  |
|  | jg3402.t1_calcyphosin-like-protein | -0.42123 | 0.016763 |  |
|  | jg9325.t1_vacuolar-protein-sorting-39 | -0.41563 | 0.004248 |  |
|  | jg11121.t1_conserved-hypothetical-protein | -0.41306 | 0 |  |
|  | jg11181.t1_RNA-binding-protein; jg16448.t1_RING-H2-finger-protein-ATL73-like | -0.4127 | 0.000463 |  |
|  | jg18238.t1_dihydrodipicolinate-reductase | -0.41222 | 0 |  |
|  | jg7507.t1_Uncharacterized-conserved-protein-contains-GRAM-domain; jg7507.t2_Uncharacterized-conserved-protein-contains-GRAM-domain | -0.41217 | 0.019166 |  |
|  | jg19537.t1_hypothetical-protein-PC110_g10389 | -0.41012 | 2.81E-05 |  |
|  | jg8539.t1_myosin-like-protein | -0.40745 | 0 |  |
|  | jg11561.t1_Alanine--glyoxylate-aminotransferase-1 | -0.40483 | 0 |  |
|  | jg4890.t1_coronin-like-protein-putative | -0.40465 | 0 |  |
|  | jg13962.t1_TKL-protein-kinase; jg18297.t1_TKL-protein-kinase | -0.40283 | 8.00E-05 |  |
|  | jg3870.t1_COP9-signalosome-complex-subunit-3 | -0.3986 | 0.042739 |  |
|  | jg19289.t1_ATP-dependent-chaperone-ClpB | -0.39325 | 0 |  |
|  | jg7232.t1_4-nitrophenylphosphatase-putative | -0.38966 | 0.003159 |  |
|  | jg19966.t1_Glutathione-synthetase | -0.38836 | 0.012997 |  |
|  | jg2176.t1_acetate-kinase | -0.3879 | 0 |  |
|  | jg15692.t1_ADP-ribosylation-factor-family | -0.38423 | 0.003301 |  |
|  | jg9488.t1_C2-domain-containing-protein-putative | -0.38266 | 0 |  |
|  | jg15138.t1_23-bisphosphoglycerate-dependent-phosphoglycerate-mutase | -0.37985 | 0 |  |
|  | jg13371.t1_Translational-activator-GCN1; jg13371.t2_Translational-activator-GCN1; jg13371.t3_Translational-activator-GCN1; jg13371.t4_Translational-activator-GCN1 | -0.3795 | 6.35E-05 |  |
|  | jg24.t1_Aldehyde-reductase; jg25.t1_Aldehyde-reductase; jg5884.t1_Aldehyde-reductase; jg5885.t1_Aldehyde-reductase | -0.37917 | 0 |  |
|  | jg11119.t1_hypothetical-protein-L917_05865 | -0.37625 | 0.003783 |  |
|  | jg10972.t1_Anthranilate-synthase-component-2 | -0.37603 | 0.000146 |  |
|  | jg7052.t1_predicted-protein | -0.37525 | 0.000141 |  |
|  | jg1159.t1_Short-chain-dehydrogenase | -0.37273 | 0.049612 |  |
|  | jg3856.t1_Putative-transcriptional-regulator-DJ-1 | -0.37055 | 0 |  |
|  | jg347.t1_glyceraldehyde-3-phosphate-dehydrogenase | -0.37031 | 0.000139 |  |
|  | jg9489.t1_C2-domain-containing-protein-putative | -0.36998 | 0.001056 |  |
|  | jg5351.t1_magnesium-transporter-protein-1-like | -0.36912 | 0.000676 |  |
|  | jg17997.t1_Dynein-heavy-chain | -0.36676 | 0.004596 |  |
|  | jg14531.t1_Histone-arginine-demethylase | -0.36088 | 0.004078 |  |
|  | jg8138.t1_PhosphoLipase-D-Pi-PLD-like-1 | -0.36008 | 2.83E-05 |  |
|  | jg5566.t1_cellulose-synthase-2 | -0.35847 | 0.001202 |  |
|  | jg17435.t1_protein-transporter-Sec61-subunit-alpha-isoform-2 | -0.35444 | 0.002051 |  |
|  | jg11006.t1_Long-chain-fatty-acid--AMP-ligase-FadD29 | -0.35372 | 0.026852 |  |
|  | jg11244.t1_hypothetical-protein-PHYSODRAFT_258942 | -0.35362 | 0.00014 |  |
|  | jg15901.t1_Puromycin-sensitive-aminopeptidase | -0.35316 | 0.006757 |  |
|  | jg1452.t1_GTP-binding-protein-YPTC4 | -0.35105 | 8.54E-05 |  |
|  | jg6962.t1_hypothetical-protein-H257_01710 | -0.34851 | 0.037474 |  |
|  | jg14385.t1_ATP-binding-Cassette-(ABC)-Superfamily | -0.34689 | 0.01426 |  |
|  | jg3381.t1_Mannitol-dehydrogenase; jg3396.t1_NADP-dependent-alcohol-dehydrogenase-C-2 | -0.34395 | 0.003932 |  |
|  | jg3074.t1_mitochondrial-carrier-protein; jg3074.t2_mitochondrial-carrier-protein | -0.34195 | 0.00086 |  |
|  | jg17066.t1_WD40-repeat-containing-protein | -0.34073 | 0.042432 |  |
|  | jg2394.t1_Target-SNARE-coiled-coil-domain | -0.33581 | 0.002476 |  |
|  | jg2788.t1_Calcium-permeable-stress-gated-cation-channel-1 | -0.33563 | 0.010193 |  |
|  | jg7266.t1_60S-ribosomal-protein-L35a-4 | -0.3325 | 2.97E-05 |  |
|  | jg9746.t1_Glutaredoxin | -0.33192 | 0.000539 |  |
|  | jg6178.t1_phosphatidylinositol-kinase | -0.32782 | 0.000755 |  |
|  | jg9602.t1_papain-like-cysteine-protease-C1 | -0.32715 | 0 |  |
|  | jg6018.t1_AGC/PDK1-protein-kinase | -0.32401 | 0.000299 |  |
|  | jg10751.t1_Aspartyl-aminopeptidase | -0.32124 | 0 |  |
|  | jg7230.t1_Actin-related-protein-2/3-complex-subunit | -0.32051 | 0.002267 |  |
|  | jg16967.t1_TKL-protein-kinase | -0.31773 | 0.04698 |  |
|  | jg7746.t1_K+-channel-protein | -0.31561 | 8.51E-05 |  |
|  | jg2733.t1_O-methyltransferase-putative | -0.31542 | 0.025166 |  |
|  | jg7249.t1_Thioredoxin-like-fold | -0.31421 | 3.25E-05 |  |
|  | jg15204.t1_Lipase-1 | -0.31119 | 0.013217 |  |
|  | jg4896.t1_actin-like-protein | -0.3109 | 0.005724 |  |
|  | jg15523.t1_40S-ribosomal-protein-S26; jg16999.t1_40S-ribosomal-protein-S26 | -0.31059 | 2.75E-05 |  |
|  | jg19012.t1_40S-ribosomal-protein-S23 | -0.30836 | 0.001057 |  |
|  | jg18642.t2_threonine-tRNA-ligase | -0.30705 | 0.001117 |  |
|  | jg5565.t1_cellulose-synthase-1 | -0.30627 | 0.000525 |  |
|  | jg15293.t1_DNA-binding-protein | -0.30445 | 0.005818 |  |
|  | jg16278.t1_biotin-[acetyl-CoA-carboxylase]-ligase | -0.29941 | 0.001752 |  |
|  | jg17288.t1_AMP-deaminase | -0.29929 | 0.013419 |  |
|  | jg19278.t1_hypothetical-protein-PHYSODRAFT_440831-partial | -0.29691 | 0.023469 |  |
|  | jg10146.t1_transient-receptor-potential-ca2-channel-(trp-cc)-family-protein | -0.29569 | 0.033451 |  |
|  | jg4904.t1_membrane-alanine-aminopeptidase | -0.29472 | 0 |  |
|  | jg15360.t1_Cystathionine-beta-synthase | -0.29358 | 0.033339 |  |
|  | jg6669.t1_putative-xylanase | -0.29156 | 0.014589 |  |
|  | jg3032.t1_peptidyl-prolyl-cis-trans-isomerase | -0.29019 | 8.48E-05 |  |
|  | jg16065.t1_glycylpeptide-N-tetradecanoyltransferase-2 | -0.28931 | 0.009644 |  |
|  | jg2192.t1_beta-galactosidase | -0.28879 | 0.009815 |  |
|  | jg19163.t1_Antibiotic-biosynthesis-monooxygenase; jg19164.t1_Antibiotic-biosynthesis-monooxygenase | -0.28793 | 6.27E-05 |  |
|  | jg15710.t1_urease-accessory-protein-UreG | -0.28544 | 8.46E-05 |  |
|  | jg1811.t1_eukaryotic-translation-initiation-factor-5 | -0.28503 | 0.004183 |  |
|  | jg19776.t1_asparagine-tRNA-ligase | -0.28485 | 0 |  |
|  | jg4386.t1_Aconitate-hydratase | -0.28481 | 3.20E-05 |  |
|  | jg5030.t1_voltage-gated-ion-channel-superfamily | -0.28431 | 0.008436 |  |
|  | jg18581.t1_Phosphate-acetyltransferase | -0.28388 | 0 |  |
|  | jg4447.t1_Pleckstrin-homology-like-domain | -0.28311 | 0.008369 |  |
|  | jg12624.t1_Nucleoredoxin-protein-1 | -0.28257 | 0.023445 |  |
|  | jg15101.t1_homoserine-kinase | -0.28121 | 0 |  |
|  | jg11648.t1_AGC/RSK/RSK-UNCLASSIFIED-protein-kinase | -0.28083 | 0.001121 |  |
|  | jg244.t1_hypothetical-protein-PHYSODRAFT_557059; jg245.t1_hypothetical-protein-PHYSODRAFT_557059 | -0.27996 | 0.0367 |  |
|  | jg8574.t2_HECT-E3-ubiquitin-ligase | -0.27983 | 0.002269 |  |
|  | jg8768.t1_acyl-CoA-binding-protein-homolog | -0.27967 | 0.00012 |  |
|  | jg19168.t1_DNA-repair-protein | -0.27946 | 0.034895 |  |
|  | jg10757.t1_dynein-light-chain-like-protein | -0.27883 | 2.68E-05 |  |
|  | jg1875.t1_CRM1-C-terminal-Exportin-1-like-protein | -0.27789 | 3.10E-05 |  |
|  | jg12589.t1_hypothetical-protein-PPTG_17957 | -0.27618 | 0.004761 |  |
|  | jg2327.t1_Argonaute1-(AGO1) | -0.27525 | 0 |  |
|  | jg4627.t1_AGC-protein-kinase | -0.27515 | 0.01194 |  |
|  | jg14252.t1_actin-related-protein-2/3-complex-subunit-putative; jg5765.t1_actin-related-protein-2/3-complex-subunit-putative | -0.27182 | 0.000371 |  |
|  | jg11740.t1_CAMK/CAMK1-protein-kinase | -0.27103 | 0.000477 |  |
|  | jg7739.t1_serine/threonine-protein-phosphatase-2A-regulatory-subunit-B'-putative | -0.27082 | 0.017012 |  |
|  | jg7366.t1_putative-glycosyl-transferase-family-48-protein | -0.26908 | 0.009718 |  |
|  | jg399.t1_Annexin-protein | -0.26792 | 0.002166 |  |
|  | jg434.t1_nicotinate-phosphoribosyltransferase | -0.26779 | 0.000146 |  |
|  | jg6350.t1_Predicted-Ca2-dependent-phospholipid-binding-protein | -0.26588 | 0.006621 |  |
|  | jg15707.t1_choline/Carnitine-O-acyltransferase-putative | -0.2648 | 0.001125 |  |
|  | jg5679.t1_Transmembrane-protein | -0.26092 | 0.001128 |  |
|  | jg1801.t1_chorismate-mutase | -0.25948 | 0.000861 |  |
|  | jg1952.t1_ATP-binding-Cassette-(ABC)-Superfamily; jg1953.t1_ATP-binding-Cassette-(ABC)-Superfamily | -0.2571 | 0.000142 |  |
|  | jg14022.t1_FT-interacting-protein-1-like | -0.2561 | 0.001994 |  |
|  | jg15061.t1_trehalose-phosphatase-variant | -0.25562 | 0.005438 |  |
|  | jg11480.t1_4-hydroxyphenylpyruvate-dioxygenase | -0.25545 | 0.014862 |  |
|  | jg9376.t1_glycine-tRNA-ligase | -0.25527 | 2.89E-05 |  |
|  | jg14217.t1_Cullin-associated-NEDD8-dissociated-protein-putative; jg14217.t2_Cullin-associated-NEDD8-dissociated-protein-1 | -0.25427 | 0.026058 |  |
|  | jg14148.t1_aspartate-semialdehyde-dehydrogenase | -0.25279 | 0.021056 |  |
|  | jg3809.t1_dolichyl-diphosphooligosaccharide--protein-glycosyltransferase-subunit-2-like | -0.25179 | 0.001089 |  |
|  | jg6347.t1_Actin-depolymerizing-factor | -0.25082 | 7.78E-05 |  |
|  | jg5119.t1_phosphoglucomutase-putative | -0.25027 | 0.042067 |  |
|  | jg13549.t1_glycolate-oxidase-subunit-GlcD | -0.24889 | 0 |  |
|  | jg6437.t1_Rab5-family-GTPase-putative | -0.24706 | 0.000138 |  |
|  | jg6476.t1_60S-ribosomal-protein-L24 | -0.24698 | 2.57E-05 |  |
|  | jg7331.t1_60S-ribosomal-protein-L6 | -0.2469 | 0.000219 |  |
|  | jg10989.t1_syntaxin-7-like-protein; jg10989.t2_syntaxin-7-like-protein | -0.24409 | 8.16E-05 |  |
|  | jg10687.t1_peptidyl-prolyl-cis-trans-isomerase-FKBP1A-like | -0.24328 | 0 |  |
|  | jg17918.t1_DnaJ-subfamily-B-protein | -0.24221 | 0.02689 |  |
|  | jg4893.t1_Serine-palmitoyltransferase | -0.24115 | 0.041629 |  |
|  | jg14364.t1_40S-ribosomal-protein-S27-like | -0.24044 | 0.000233 |  |
|  | jg4233.t1_trehalose-phosphatase | -0.23857 | 0.016651 |  |
|  | jg2797.t1_calpain-like-protein; jg2797.t2_calpain-like-protein | -0.23778 | 0.002361 |  |
|  | jg677.t1_Ubiquitin-system-component-Cue | -0.23731 | 2.78E-05 |  |
|  | jg1193.t1_Isocitrate-lyase | -0.23612 | 2.88E-05 |  |
|  | jg14349.t1_Uncharacterized-conserved-protein | -0.23536 | 0 |  |
|  | jg8803.t1_Mitochondrial-Carrier-(MC)-Family | -0.2332 | 6.21E-05 |  |
|  | jg16893.t2_Secretory-protein-OPEL | -0.2323 | 0.001532 |  |
|  | jg3902.t1_rna-recognition-motif-domain-containing-protein | -0.23124 | 0.005235 |  |
|  | jg10496.t1_Myosin-like-protein; jg9146.t1_Myosin-like-protein | -0.23098 | 0.001582 |  |
|  | jg8950.t1_40S-ribosomal-protein-S16 | -0.23084 | 0.000378 |  |
|  | jg3457.t1_Demethylmenaquinone-methyltransferase | -0.22839 | 0.029074 |  |
|  | jg11771.t1_NADH-cytochrome-b5-reductase | -0.22821 | 0.00264 |  |
|  | jg343.t1_60S-ribosomal-protein-L23 | -0.22761 | 8.33E-05 |  |
|  | jg9910.t1_Linear-gramicidin-synthase-subunit-D | -0.22635 | 0.000758 |  |
|  | jg7522.t1_exportin-2-like-protein | -0.22633 | 0.001529 |  |
|  | jg16009.t1_methionine-tRNA-ligase | -0.22481 | 0.002637 |  |
|  | jg13378.t1_argininosuccinate-synthase | -0.22446 | 0 |  |
|  | jg13407.t1_arginine-tRNA-ligase | -0.22336 | 0.005344 |  |
|  | jg2332.t1_Tripeptidyl-peptidase-2 | -0.22206 | 0.000577 |  |
|  | jg16684.t1_40S-ribosomal-protein-S29 | -0.21792 | 0.022879 |  |
|  | jg1063.t1_PpiC-type-peptidyl-prolyl-cis-trans-isomerase-putative; jg2607.t1_PpiC-type-peptidyl-prolyl-cis-trans-isomerase-putative | -0.21691 | 0.001383 |  |
|  | jg2990.t1_Tubby-C-terminal-like-domain; jg697.t1_Tubby-C-terminal-like-domain | -0.21601 | 0 |  |
|  | jg66.t1_ubiquinone-menaquinone-biosynthesis-methyltransferase | -0.21468 | 0.029575 |  |
|  | jg18485.t1_F-actin-capping-protein-subunit-beta | -0.21418 | 0.000693 |  |
|  | jg16685.t1_Dolichyl-diphosphooligosaccharide-protein-glycosyltransferase | -0.21348 | 0.001308 |  |
|  | jg8473.t1_peroxisomal-acyl-coenzyme-A-oxidase-putative | -0.21235 | 0.043008 |  |
|  | jg10691.t1_Homoserine-dehydrogenase | -0.20959 | 0.000142 |  |
|  | jg7502.t1_callose-synthase-putative | -0.20708 | 0.001324 |  |
|  | jg1564.t1_alcohol-dehydrogenase-putative | -0.20522 | 0.00128 |  |
|  | jg2872.t1_Vacuolar-protein-sorting-associated-protein-8 | -0.20514 | 0.030708 |  |
|  | jg7283.t1_RAN-GTPase-activating-protein-2 | -0.20205 | 2.93E-05 |  |
|  | jg12352.t1_peptidyl-prolyl-isomerase-FKBP12 | -0.20088 | 0.002622 |  |
|  | jg7679.t1_Ras-related-protein-Rab-8A | -0.19874 | 0.02977 |  |
|  | jg15964.t1_Proteasome-subunit-beta | -0.19626 | 0.000687 |  |
|  | jg11527.t1_Neutral-ceramidase | -0.19617 | 0.002078 |  |
|  | jg6988.t1_Vacuolar-protein-sorting-associated-protein | -0.19353 | 0.008772 |  |
|  | jg6528.t1_Eukaryotic-translation-elongation-factor-1-epsilon-1 | -0.19274 | 0.024738 |  |
|  | jg14814.t1_archaeal-ribosomal-protein-S17P | -0.1924 | 0.000484 |  |
|  | jg7682.t1_phospholipase-D-Pi-PXPH-PLD | -0.19178 | 0.013892 |  |
|  | jg5982.t1_60S-ribosomal-protein-L30 | -0.19084 | 0.001264 |  |
|  | jg18380.t1_40S-ribosomal-protein-S8 | -0.19052 | 0.001816 |  |
|  | jg8108.t1_histone-demethylase | -0.1842 | 0.004677 |  |
|  | jg19099.t1_40S-ribosomal-protein | -0.18392 | 0.000145 |  |
|  | jg11076.t1_Eukaryotic-translation-initiation-factor-2-subunit-3 | -0.18351 | 0.000751 |  |
|  | jg9392.t1_Chorismate-synthase | -0.18244 | 0.002382 |  |
|  | jg4844.t2_Myosin-like-protein | -0.18183 | 0.01691 |  |
|  | jg12919.t1_Acetylornithine-deacetylase | -0.18102 | 0.000367 |  |
|  | jg11077.t1_Leucine-tRNA-ligase | -0.1774 | 0.003486 |  |
|  | jg17919.t1_hypothetical-protein-PHPALM_10176 | -0.17701 | 0.025505 |  |
|  | jg12652.t1_DEAD-box-ATP-dependent-RNA-helicase-8 | -0.17661 | 0.011894 |  |
|  | jg13924.t2_Vacuolar-protein-sorting-associated-protein-13a | -0.17651 | 0.000521 |  |
|  | jg2347.t1_3-isopropylmalate-dehydrogenase | -0.17635 | 0.00167 |  |
|  | jg18828.t1_fimbrin-like-protein | -0.17549 | 0.006953 |  |
|  | jg6539.t1_hypothetical-protein-PHYSODRAFT_500291 | -0.1738 | 0.006297 |  |
|  | jg19555.t1_glutamate-tRNA-ligase | -0.17301 | 2.62E-05 |  |
|  | jg15717.t1_3-phosphoshikimate-1-carboxyvinyltransferase | -0.1728 | 0.003474 |  |
|  | jg14781.t1_aldose-1-epimerase-putative | -0.17215 | 0.000379 |  |
|  | jg7786.t1_endo1-putative | -0.17168 | 2.70E-05 |  |
|  | jg8614.t1_serine/threonine-protein-phosphatase-PP2A | -0.16989 | 3.33E-05 |  |
|  | jg2767.t1_serine/threonine-protein-kinase | -0.1696 | 0.000507 |  |
|  | jg17061.t1_putative-ubiquitin-activating-enzyme; jg17061.t2_putative-ubiquitin-activating-enzyme | -0.16896 | 0.005347 |  |
|  | jg1708.t1_actin-depolymerizing-factor; jg6400.t1_actin-depolymerizing-factor | -0.16869 | 0.00227 |  |
|  | jg15742.t1_Rab18-family-GTPase-putative | -0.16815 | 0.003961 |  |
|  | jg17924.t1_glutamate-decarboxylase | -0.16744 | 8.07E-05 |  |
|  | jg17769.t1_Mannose-1-phosphate-guanyltransferase-beta | -0.16525 | 0.010484 |  |
|  | jg4799.t1_leukotriene-A-4-hydrolase/aminopeptidase | -0.16517 | 0.021531 |  |
|  | jg2885.t1_Serine-palmitoyltransferase | -0.16335 | 0.005231 |  |
|  | jg1998.t1_GTP-binding-nuclear-protein-ran-1 | -0.16164 | 0.002842 |  |
|  | jg9795.t1_RNA-binding-protein | -0.16147 | 0.033545 |  |
|  | jg1647.t1_Acylamino-acid-releasing-enzyme | -0.16091 | 0.039678 |  |
|  | jg12394.t1_C2-domain-containing-protein-putative | -0.16049 | 0.027877 |  |
|  | jg7245.t1_40S-ribosomal-protein-S18 | -0.16023 | 0.002374 |  |
|  | jg16361.t1_fatty-acid-synthase-subunit-alpha-putative | -0.16015 | 0.008083 |  |
|  | jg16912.t1_actin-like-protein | -0.15683 | 0.001062 |  |
|  | jg9370.t1_Transketolase | -0.15465 | 0 |  |
|  | jg4604.t1_tryptophan-biosynthesis-protein-TRP1 | -0.15395 | 2.66E-05 |  |
|  | jg7269.t1_60S-ribosomal-protein-L27 | -0.15222 | 0.000324 |  |
|  | jg12364.t1_Peptidyl-prolyl-cis-trans-isomerase-pin1; jg98.t1_Peptidyl-prolyl-cis-trans-isomerase-pin1 | -0.15189 | 0.009655 |  |
|  | jg11514.t1_Transcriptional-repressor-TUP1-like-protein | -0.14935 | 0.000378 |  |
|  | jg8776.t1_ribosomal-protein-RPL26 | -0.14846 | 0.011668 |  |
|  | jg9979.t1_ras-protein-let-60 | -0.14736 | 0.005584 |  |
|  | jg7171.t1_Rab7-family-GTPase | -0.14109 | 0.010071 |  |
|  | jg11100.t1_HIT-domain-containing-protein-putative | -0.1409 | 0.022596 |  |
|  | jg1696.t1_uracil-phosphoribosyltransferase; jg6411.t1_uracil-phosphoribosyltransferase | -0.13974 | 0.018857 |  |
|  | jg6449.t1_Coatomer-subunit-alpha-1 | -0.13937 | 0.000124 |  |
|  | jg17980.t1_anthranilate-synthase-component-I | -0.13935 | 0.000877 |  |
|  | jg19895.t1_heat-shock-protein-70 | -0.13849 | 2.69E-05 |  |
|  | jg2720.t1_ATLS1-like-light-inducible-protein | -0.13483 | 0.012362 |  |
|  | jg12993.t2_phosphoribosylaminoimidazolesuccinocarboxamide-synthase | -0.13425 | 0.018015 |  |
|  | jg730.t1_Guanylate-binding-protein | -0.13205 | 0.03582 |  |
|  | jg5180.t1_predicted-protein | -0.13153 | 0.003795 |  |
|  | jg10217.t1_histone-H4; jg17078.t1_histone-H4; jg7580.t1_histone-H4 | -0.12996 | 0.001825 |  |
|  | jg14270.t1_glucose-6-phosphate-isomerase; jg5749.t1_glucose-6-phosphate-isomerase | -0.12967 | 3.19E-05 |  |
|  | jg7800.t1_60S-ribosomal-protein-L38 | -0.12918 | 0.030319 |  |
|  | jg17399.t1_60S-ribosomal-protein | -0.12895 | 0.007111 |  |
|  | jg14492.t1_CAMK/CAMKL/AMPK-protein-kinase | -0.12888 | 0.031057 |  |
|  | jg5581.t1_ABC-transporter-E-family-member-2 | -0.12832 | 0.03967 |  |
|  | jg3618.t1_Phosphoglucomutase | -0.12726 | 8.21E-05 |  |
|  | jg12936.t2_AP-2-complex-subunit-beta | -0.12708 | 0.021062 |  |
|  | jg8040.t1_translationally-controlled-tumor-protein | -0.12697 | 0.037315 |  |
|  | jg1177.t1_Inorganic-pyrophosphatase | -0.12583 | 0.001009 |  |
|  | jg3947.t1_40S-ribosomal-protein-S14 | -0.12463 | 0.004812 |  |
|  | jg6031.t1_phenylalanine-tRNA-ligase-alpha-subunit | -0.12252 | 0.000323 |  |
|  | jg10413.t1_ADP-ribosylation/Crystallin-J1 | -0.12178 | 0.001529 |  |
|  | jg3781.t1_Nucleotidylyl-transferase-superfamily-protein | -0.12098 | 0.003467 |  |
|  | jg1924.t1_Inositol-polyphosphate-5-phosphatase-and-related-proteins | -0.1207 | 0.0012 |  |
|  | jg17941.t1_60S-ribosomal-protein | -0.11805 | 0.047906 |  |
|  | jg16757.t1_nuclear-transport-factor-2-family-protein | -0.11624 | 0.027668 |  |
|  | jg15065.t1_aspartic-protease; jg15065.t2_aspartic-protease | -0.11514 | 0.039756 |  |
|  | jg11793.t1_Phosphoethanolamine-N-methyltransferase | -0.1142 | 0.004056 |  |
|  | jg6991.t1_26S-proteasome-non-ATPase-regulatory-subunit-putative | -0.11378 | 0.001672 |  |
|  | jg19891.t1_histidine-tRNA-ligase | -0.11263 | 0.031283 |  |
|  | jg15662.t1_protein-disulfide-isomerase-domain | -0.11226 | 0.041762 |  |
|  | jg11431.t1_Phosphoacetylglucosamine-mutase; jg11431.t2_Phosphoacetylglucosamine-mutase | -0.11105 | 0.001942 |  |
|  | jg1600.t1_Metalloprotease-family-M17; jg966.t1_Metalloprotease-family-M17 | -0.10909 | 0.000478 |  |
|  | jg17114.t1_Transaldolase | -0.10517 | 0.000114 |  |
|  | jg13845.t1_glutathione-disulfide-reductase; jg1555.t1_glutathione-disulfide-reductase | -0.10442 | 7.89E-05 |  |
|  | jg10028.t1_calcium-activated-potassium-channel-subunit-alpha | -0.10374 | 0.026085 |  |
|  | jg15834.t1_NAD(P)H:quinone-oxidoreductase-type-IV | -0.10217 | 0.028118 |  |
|  | jg13033.t1_Orotidine-5'-phosphate-decarboxylase | -0.10208 | 0.011589 |  |
|  | jg6207.t1_60S-ribosomal-protein-L7 | -0.09838 | 0.013828 |  |
|  | jg11534.t1_phosphoribosylformylglycinamidine-synthase | -0.0978 | 0.0066 |  |
|  | jg11084.t1_60S-ribosomal-protein-L18-2 | -0.09624 | 0.048368 |  |
|  | jg7210.t1_60S-ribosomal-protein-L9 | -0.09367 | 0.009415 |  |
|  | jg3111.t1_TKL-protein-kinase | -0.09323 | 0.012462 |  |
|  | jg1184.t1_ubiquitin-activating-enzyme-E1-family-putative | -0.0928 | 0.016294 |  |
|  | jg1795.t1_Unconventional-myosin-X | -0.08586 | 0.013371 |  |
|  | jg16387.t1_translation-elongation-factor-aEF-2 | -0.0851 | 7.95E-05 |  |
|  | jg12809.t1_UDP-glucose-6-dehydrogenase | -0.08174 | 0.015987 |  |
|  | jg11667.t1_coatomer-subunit-beta | -0.08086 | 0.010807 |  |
|  | jg10865.t1_5-oxoprolinase | -0.08055 | 0.007347 |  |
|  | jg18348.t1_40S-ribosomal-protein-S2 | -0.07916 | 0.040783 |  |
|  | jg7313.t1_pyruvate-dehydrogenase-(acetyl-transferring)-E1-component-alpha-subunit | -0.07779 | 0.00405 |  |
|  | jg2711.t1_60S-ribosomal-protein-L18a | -0.07118 | 0.016639 |  |
|  | jg7043.t1_Phenylalanine-tRNA-ligase-beta-subunit | -0.06844 | 0.043238 |  |
|  | jg7015.t1_Ubiquitin-carboxyl-terminal-hydrolase | -0.06489 | 0.027389 |  |
|  | jg13246.t1_T-complex-protein-1-theta-subunit | -0.04424 | 0.03946 |  |
|  | jg7109.t1_26S-proteasome-non-ATPase-regulatory-subunit-2 | -0.04328 | 0.042054 |  |
| **Upregulated List** | | | | |
|  | jg15744.t1_ GTPase-(Era) | 4.32936 | 0 |  |
|  | jg14521.t1_Dual-specificity-protein-phosphatase-CDC14B | 3.81314 | 0 |  |
|  | jg2191.t1_alpha-13-mannosyltransferase-putative | 3.72082 | 0 |  |
|  | jg5118.t1_ADP-ribosylation-factor | 3.65733 | 2.72E-05 |  |
|  | jg9448.t1_ADP-ribosylation-factor-family | 3.00253 | 0 |  |
|  | jg2949.t1_Voltage-dependent-P/Q-type-calcium-channel-subunit-alpha-1A | 2.93095 | 0 |  |
|  | jg3331.t1_S-adenosylmethionine-synthase-2 | 2.88528 | 0.003385 |  |
|  | jg13567.t1_Catalase/peroxidase-HPI; jg13568.t1_Catalase/peroxidase-HPI | 2.76101 | 0 |  |
|  | jg13217.t1_Ornithine-decarboxylase | 2.58301 | 0 |  |
|  | jg11424.t1_mitochondrial-chaperone-BCS1-B; jg11425.t1_ mitochondrial-chaperone-BCS1-B; jg11426.t1_ mitochondrial-chaperone-BCS1-B; jg11426.t2_ mitochondrial-chaperone-BCS1-B; jg11427.t1_mitochondrial-chaperone-BCS1-B; jg5715.t1_mitochondrial-chaperone-BCS1-B; jg5716.t1_mitochondrial-chaperone-BCS1-B | 2.51881 | 0 |  |
|  | jg7740.t1_Elongation-factor-3 | 2.36371 | 0 |  |
|  | jg1171.t1_Major-vault-protein | 2.34329 | 0 |  |
|  | jg8536.t1_CAMK-protein-kinase; jg8536.t2_CAMK-protein-kinase | 2.23993 | 0 |  |
|  | jg10741.t1_glutamine-fructose-6-phosphate-transaminase-(isomerizing) | 2.22134 | 0 |  |
|  | jg10504.t1_transmembrane-protein-65-like | 2.11033 | 8.43E-05 |  |
|  | jg17566.t1_hypothetical-protein-L915_07795 | 2.10689 | 8.12E-05 |  |
|  | jg9531.t1_Bifunctional-epoxide-hydrolase-2 | 2.10199 | 0 |  |
|  | jg18709.t1_Short/branched-chain-specific-acyl-CoA-dehydrogenase | 2.07094 | 0 |  |
|  | jg7441.t1_serine-protease-family-S10-putative | 2.05749 | 0 |  |
|  | jg8788.t1_Pyridoxal-biosynthesis-lyase-pdxS | 1.95398 | 0 |  |
|  | jg5346.t1_histidine-acid-phosphatase-putative | 1.93679 | 0.000688 |  |
|  | jg127.t1_Type-II-(General)-Secretory-Pathway-(IISP)-Family | 1.93294 | 0 |  |
|  | jg18922.t1_nitrite-reductase-[NAD(P)H]-large-subunit | 1.89863 | 0 |  |
|  | jg12915.t1_Pterin-4-alpha-carbinolamine-dehydratase | 1.77325 | 0 |  |
|  | jg2190.t1_ GDP-Man:Man(3)GlcNAc(2)-PP-Dol alpha-1,2-mannosyltransferase | 1.72112 | 0 |  |
|  | jg13558.t1_conserved-hypothetical-protein | 1.60518 | 2.79E-05 |  |
|  | jg13696.t1_C-Myc-binding-protein-putative | 1.57292 | 0.000123 |  |
|  | jg12435.t1_FAD-binding-protein; jg724.t1_FAD-binding-protein | 1.56244 | 0 |  |
|  | jg4276.t1_quinolinate-synthetase-A-protein-putative | 1.45823 | 0 |  |
|  | jg6818.t1_glycerol-3-phosphate-dehydrogenase-(NAD(+)) | 1.44709 | 0 |  |
|  | jg14694.t1_cystathionine-beta-lyase | 1.43736 | 3.15E-05 |  |
|  | jg3669.t1_octopine-dehydrogenase | 1.40491 | 0 |  |
|  | jg6266.t1_Serine-protease-family-S10 | 1.39936 | 6.25E-05 |  |
|  | jg18278.t1_3-ketoacyl-CoA-thiolase-A | 1.38669 | 0 |  |
|  | jg16779.t1_Acetyl-coenzyme-A-synthetase | 1.3186 | 0.004189 |  |
|  | jg4795.t1_serine-protease-family | 1.29274 | 2.55E-05 |  |
|  | jg10807.t1_15-hydroxyprostaglandin-dehydrogenase | 1.26639 | 0 |  |
|  | jg19950.t1_peroxisomal-hydratase-dehydrogenase-epimerase | 1.25522 | 0 |  |
|  | jg10919.t1_3-methyl-2-oxobutanoate-hydroxymethyltransferase | 1.25407 | 0 |  |
|  | jg9450.t1_NAD(P)H:quinone-oxidoreductase-type-IV | 1.23956 | 0.000116 |  |
|  | jg13405.t1_Jacalin-like-lectin-domain-containing-protein | 1.21283 | 0.003376 |  |
|  | jg10916.t1_Mitochondrial-ribosomal-protein-L37-isoform-1 | 1.20166 | 0.009049 |  |
|  | jg1722.t1_TBCC-domain-containing-protein-1 | 1.20029 | 0.008156 |  |
|  | jg18846.t1_geranylgeranyl-pyrophosphate-synthetase | 1.19363 | 2.67E-05 |  |
|  | jg12357.t1_Peroxisomal-membrane-protein-PEX16 | 1.18754 | 0.000411 |  |
|  | jg7117.t1_superoxide-disumtase | 1.17623 | 0 |  |
|  | jg13586.t1_Mitochondrial-substrate-carrier-family-protein-B; jg17159.t1_Mitochondrial-substrate-carrier-family-protein-B | 1.16472 | 0.000591 |  |
|  | jg7370.t1_Prenylated-rab-acceptor-1 | 1.15262 | 0.004603 |  |
|  | jg13726.t1_Medium-chain-specific-acyl-CoA-dehydrogenase-mitochondrial-precursor | 1.14674 | 0 |  |
|  | jg5992.t1_Proteasome-activator-subunit-1 | 1.12459 | 0 |  |
|  | jg14192.t1_Glycoside-hydrolase; jg15890.t1_Glycoside-hydrolase | 1.10244 | 0 |  |
|  | jg19550.t1_DmX-protein-1; jg19553.t1_DmX-protein-1 | 1.0946 | 0.015006 |  |
|  | jg2413.t1_Transcription-factor-S; jg3210.t1_Transcription-factor-S | 1.09166 | 0.001127 |  |
|  | jg9410.t1_Acetyl-CoA-acetyltransferase | 1.08835 | 3.05E-05 |  |
|  | jg4344.t1_hypothetical-protein-PHYSODRAFT_517413 | 1.08761 | 2.87E-05 |  |
|  | jg4242.t1_eukaryotic-initiation-factor-4E-putative | 1.07914 | 0.004193 |  |
|  | jg3523.t1_iron-sulfur-cluster-scaffold-protein-nfu-like-protein | 1.06772 | 0.000121 |  |
|  | jg2372.t1_Sua5/YciO/YrdC/YwlC-family-protein | 1.06192 | 0.001132 |  |
|  | jg17170.t1_12-oxophytodienoate-reductase; jg17171.t1_12-oxophytodienoate-reductase; jg17174.t1_12-oxophytodienoate-reductase | 1.05289 | 0.008278 |  |
|  | jg19707.t1_12-oxophytodienoate-reductase-putative | 1.04798 | 0 |  |
|  | jg13196.t1_Glutathione-S-transferase | 1.0456 | 6.23E-05 |  |
|  | jg14188.t1_Rhamnolipids-biosynthesis-3-oxoacyl--reductase; jg15887.t1_Rhamnolipids-biosynthesis-3-oxoacyl--reductase | 1.04303 | 0.000576 |  |
|  | jg11348.t1_Long-chain-acyl-CoA-synthetase-7-peroxisomal | 1.04287 | 0 |  |
|  | jg19821.t1_Transcriptional-coactivator-multiprotein-bridging-factor-Mbf1-(predicted) | 1.04035 | 0.000122 |  |
|  | jg3658.t1_Dihydrolipoyllysine-residue-succinyltransferase | 1.03623 | 0.003004 |  |
|  | jg219.t1_acyl-CoA-dehydrogenase-putative | 1.03363 | 0 |  |
|  | jg5854.t1_Predicted-methyltransferase | 1.02793 | 0.003771 |  |
|  | jg9911.t1_acetyl-CoA-carboxylase-biotin-carboxylase-subunit | 1.02253 | 0 |  |
|  | jg3519.t1_Armadillo-repeat-protein-VAC8-required-for-vacuole-fusion-inheritance-and-cytosol-to-vacuole-protein-targeting | 1.02052 | 0.00614 |  |
|  | jg5600.t1_Maltose-O-acetyltransferase | 1.01233 | 0.000147 |  |
|  | jg19971.t1_hypothetical-protein-PHYSODRAFT_285825 | 1.00901 | 0.004308 |  |
|  | jg12413.t1_Ferrochelatase | 0.99463 | 0.000442 |  |
|  | jg10666.t1_proteasome-subunit-beta-type-6-putative | 0.994229 | 0 |  |
|  | jg5611.t1_Cystathionine-gamma-synthase | 0.983095 | 0 |  |
|  | jg17326.t1_Avr1b-1-avirulence-like-protein | 0.982972 | 0.000996 |  |
|  | jg3912.t1_vacuolar-sorting-receptor-putative | 0.969589 | 0 |  |
|  | jg12374.t1_ATP-dependent-protease-HslVU-ATPase-subunit; jg12485.t1_ATP-dependent-protease-HslVU-ATPase-subunit | 0.964135 | 8.19E-05 |  |
|  | jg10143.t1_ATP-binding-cassette-(ABC)-Superfamily; jg10143.t2_ ATP-binding-cassette-(ABC)-Superfamily | 0.960178 | 0 |  |
|  | jg13967.t1_methylcrotonoyl-CoA-carboxylase-beta-chain-mitochondrial | 0.957329 | 0 |  |
|  | jg16780.t1_acetate-CoA-ligase | 0.935769 | 0 |  |
|  | jg6269.t1_3-ketoacyl-CoA-thiolase-mitochondrial | 0.929195 | 0 |  |
|  | jg2914.t1_Actin-binding-protein-F | 0.925455 | 3.36E-05 |  |
|  | jg12611.t1_Mitochondrial-Protein-Translocase-(MPT)-Family-TIM44 | 0.90653 | 2.63E-05 |  |
|  | jg18585.t1_Catalase | 0.889221 | 0 |  |
|  | jg12264.t1_Vacuolar-iron-transporter-1 | 0.876849 | 0.008614 |  |
|  | jg7708.t1_Kynurenine-oxoglutarate-transaminase | 0.874974 | 8.28E-05 |  |
|  | jg7405.t1_cell-division-protein-ftsZ | 0.872992 | 0.034198 |  |
|  | jg7127.t1_N-acetyl-gamma-glutamyl-phosphate-reductase | 0.872103 | 0 |  |
|  | jg17113.t1_acyl-CoA-dehydrogenase | 0.856479 | 0.005325 |  |
|  | jg11021.t1_12-oxophytodienoate-reductase-putative | 0.856405 | 0.045394 |  |
|  | jg1155.t1_Serine-protease-family-S33 | 0.852879 | 0.00121 |  |
|  | jg18343.t1_Mitochondrial-Carrier-(MC)-Family | 0.848955 | 0.001478 |  |
|  | jg2198.t1_Chaperone-dnaK | 0.847962 | 0 |  |
|  | jg17708.t1_Pre-mRNA-splicing-factor-Crooked-neck-like-protein | 0.847092 | 0.000233 |  |
|  | jg1279.t1_Transmembrane-prolyl-4-hydroxylase | 0.844408 | 0.003875 |  |
|  | jg19008.t1_Glutathione-transferase-theta-class | 0.844117 | 0.001064 |  |
|  | jg16803.t1_Hydroxymethylglutaryl-CoA-lyase | 0.843032 | 0.000526 |  |
|  | jg13539.t1_3-hydroxyacyl-CoA-dehydrogenase-type-2 | 0.83884 | 0.000146 |  |
|  | jg4080.t1_ATP-dependent-chaperone-ClpB | 0.820759 | 0.031008 |  |
|  | jg15268.t1_Cysteine-desulfurase-IscS | 0.816449 | 0 |  |
|  | jg13384.t1_Maleylacetoacetate-isomerase | 0.810386 | 0 |  |
|  | jg12137.t1_Peroxisomal-multifunctional-enzyme-type-2 | 0.792717 | 0 |  |
|  | jg13298.t1_Phenylalanine-4-hydroxylase-henna-like-protein | 0.788593 | 0.020026 |  |
|  | jg1292.t1_ Mitochondrial-import-inner-membrane-translocase-subunit-Tim10 | 0.777348 | 0.000139 |  |
|  | jg8736.t1_Luminal-binding-protein | 0.776666 | 0 |  |
|  | jg18001.t1_LETM1-and-EF-hand-domain-containing-protein-1 | 0.775877 | 0 |  |
|  | jg8243.t2_Glycerol-3-phosphate-dehydrogenase | 0.771686 | 0 |  |
|  | jg14633.t1_Glycerol-kinase | 0.769314 | 0 |  |
|  | jg12684.t1_3-hydroxyisobutyrate-dehydrogenase | 0.759667 | 3.35E-05 |  |
|  | jg11970.t1_hypothetical-protein-PHYSODRAFT_553371; jg2265.t1_hypothetical-protein-PHYSODRAFT_553371 | 0.754274 | 0.002333 |  |
|  | jg12616.t1_Thioredoxin-dependent-peroxide-reductase | 0.744285 | 0 |  |
|  | jg1582.t1_LYR-motif-containing-protein-4; jg985.t1_LYR-motif-containing-protein-4 | 0.742408 | 2.99E-05 |  |
|  | jg19128.t1_inosine-5-monophosphate-dehydrogenase | 0.741702 | 0 |  |
|  | jg16352.t1_ribosome-recycling-factor | 0.740623 | 0.000368 |  |
|  | jg19937.t1_alpha-actinin-1-putative | 0.738188 | 0 |  |
|  | jg3028.t1_dihydrolipoyllysine-residue-succinyltransferase-component-of-2-oxoglutarate-dehydrogenase | 0.735531 | 2.74E-05 |  |
|  | jg11765.t1_peptide-methionine-(S)-S-oxide-reductase | 0.729486 | 8.09E-05 |  |
|  | jg9804.t1_arginase | 0.726615 | 0 |  |
|  | jg19414.t1_xylose-isomerase; jg19415.t1_xylose-isomerase | 0.720186 | 0.00156 |  |
|  | jg8024.t1_Nucleolar-protein-NOP5 | 0.718203 | 0 |  |
|  | jg6303.t1_Sideroflexin-1-like-protein | 0.714088 | 0.014222 |  |
|  | jg6187.t1_Ubiquitin-system-component-Cue | 0.697916 | 0.000122 |  |
|  | jg17483.t1_Mitochondrial-39-S-ribosomal-protein-L47 | 0.697719 | 0.010268 |  |
|  | jg19188.t1_RNA-helicase | 0.69547 | 0.00022 |  |
|  | jg17079.t1_histone-H3; jg7579.t1_histone-H3 | 0.692295 | 0.034795 |  |
|  | jg8766.t1_protein-phosphatase-2C-putative | 0.691603 | 0.000154 |  |
|  | jg1624.t1_Mitochondrial-import-inner-membrane-translocase-subunit-tim16 | 0.691379 | 0.000115 |  |
|  | jg13991.t1_family-30-glycoside-hydrolase | 0.690062 | 0.000688 |  |
|  | jg18750.t1_Translation-initiation-factor-IF-2 | 0.684707 | 0.006274 |  |
|  | jg8021.t1_D-mannonate-epimerase | 0.68311 | 2.82E-05 |  |
|  | jg218.t1_Short-chain-specific-acyl-CoA-dehydrogenase-mitochondrial-precursor | 0.677957 | 0 |  |
|  | jg18243.t1_Mercaptopyruvate-sulfurtransferase/thiosulfate-sulfurtransferase | 0.675245 | 0 |  |
|  | jg19985.t1_thioredoxin-like-protein | 0.674443 | 0.014751 |  |
|  | jg9678.t1_Glutathionyl-hydroquinone-reductase-YqjG | 0.674379 | 0 |  |
|  | jg5555.t1_acyl-CoA-dehydrogenase-family-member-9-mitochondrial-precursor | 0.670624 | 6.19E-05 |  |
|  | jg17883.t1_nucleolin-putative; jg19223.t1_nucleolin-putative | 0.66836 | 0.009941 |  |
|  | jg10210.t1_5-carboxymethyl-2-hydroxymuconate-isomerase | 0.662418 | 0 |  |
|  | jg15799.t1_NAD(P)H:quinone-oxidoreductase-type-IV; jg15804.t1_NAD(P)H:quinone-oxidoreductase-type-IV | 0.658027 | 0.01081 |  |
|  | jg16611.t1_oxoglutarate-dehydrogenase-(succinyl-transferring)-E1-component; jg8709.t1_oxoglutarate-dehydrogenase-(succinyl-transferring)-E1-component | 0.653057 | 0 |  |
|  | jg3992.t1_Vesicle-associated-membrane-protein | 0.652643 | 0.000124 |  |
|  | jg3169.t1_serine-protease-family-S10-putative | 0.651126 | 0.000306 |  |
|  | jg9926.t1_Mitochondrial-ribosomal-protein-RSM27 | 0.650161 | 0.031122 |  |
|  | jg592.t1_delta-l-pyrroline-5-carboxylate-synthetase | 0.642053 | 0 |  |
|  | jg18279.t1_Xaa-Pro-dipeptidase | 0.64072 | 0.038938 |  |
|  | jg12077.t1_Myosin-I-heavy-chain | 0.638722 | 0 |  |
|  | jg7769.t1_2-isopropylmalate-synthase | 0.635764 | 0 |  |
|  | jg8740.t1_Mitochondrial-ribosomal-protein-L22 | 0.635238 | 0.035203 |  |
|  | jg9918.t1_eukaryotic-initiation-factor-4E-putative | 0.628942 | 0.000116 |  |
|  | jg13531.t1_Nucleolar-protein-Nop56 | 0.626365 | 0 |  |
|  | jg6416.t1_Urate-catabolism-protein | 0.624191 | 0.018753 |  |
|  | jg5297.t1_oxoglutarate-dehydrogenase-(succinyl-transferring)-E1-component | 0.621906 | 0 |  |
|  | jg7822.t1_mitochondrial-import-inner-membrane-translocase-subunit-tim9 | 0.620914 | 0.039958 |  |
|  | jg10918.t1_DnaJ-subfamily-C-member-15 | 0.617062 | 0 |  |
|  | jg13471.t1_calcineurin-like-phosphoesterase-putative | 0.617002 | 0.006367 |  |
|  | jg1719.t1_Fbxo7/PI31-domain | 0.613908 | 0.000983 |  |
|  | jg13839.t1_translation-elongation-factor-Ts; jg5369.t1_translation-elongation-factor-Ts | 0.61381 | 3.09E-05 |  |
|  | jg9002.t1_Mitochondrial-ribosomal-protein-L17 | 0.613414 | 0.005519 |  |
|  | jg18631.t1_sulfate-adenylyltransferase | 0.610947 | 0.001673 |  |
|  | jg19161.t1_antibiotic-biosynthesis-monooxygenase; jg19166.t1_antibiotic-biosynthesis-monooxygenase | 0.610302 | 0 |  |
|  | jg15820.t1_hypothetical-protein-PHYSODRAFT_354620 | 0.609303 | 0.000382 |  |
|  | jg15396.t1_GPI-anchored-protein | 0.607445 | 0.00012 |  |
|  | jg11734.t1_Iron-sulfur-cluster-assembly-protein | 0.603279 | 0.026891 |  |
|  | jg13867.t1_Abnormal-spindle-microcephaly-associated-protein | 0.603096 | 0.000693 |  |
|  | jg14366.t2_methylmalonate-semialdehyde-dehydrogenase-(acylating) | 0.598644 | 0 |  |
|  | jg1323.t2_aldehyde-dehydrogenase | 0.597665 | 0 |  |
|  | jg4971.t1_RxLR-like-protein | 0.597465 | 3.23E-05 |  |
|  | jg4925.t1_hypothetical-protein-PHYSODRAFT_557115 | 0.59493 | 0.037109 |  |
|  | jg10256.t1_putative-GPI-anchor-protein | 0.593701 | 0.009244 |  |
|  | jg10418.t1_Dihydrolipoamide-s-acetyltransferase | 0.591581 | 0.006107 |  |
|  | jg11639.t1_dihydrolipoamide-succinyltransferase-putative | 0.588622 | 2.92E-05 |  |
|  | jg10750.t1_propionyl-CoA-carboxylase-beta-chain-mitochondrial | 0.587155 | 6.33E-05 |  |
|  | jg7716.t1_2-amino-3-ketobutyrate-coenzyme-A-ligase | 0.582548 | 8.41E-05 |  |
|  | jg8400.t1_Choline/Carnitine-O-acyltransferase | 0.582269 | 0.000219 |  |
|  | jg4960.t1_peptidyl-prolyl-cis-trans-isomerase-3 | 0.579731 | 0.048998 |  |
|  | jg1192.t1_Peptidyl-prolyl-cis-trans-isomerase | 0.578866 | 0.00281 |  |
|  | jg7805.t1_50S-ribosomal-protein-L4 | 0.578366 | 3.07E-05 |  |
|  | jg3904.t1_phosphoserine-phosphatase-SerB | 0.577905 | 0.005158 |  |
|  | jg1771.t1_ribosomal-protein-L9 | 0.576494 | 7.98E-05 |  |
|  | jg2673.t1_calcineurin-like-phosphoesterase-putative | 0.575148 | 0.000139 |  |
|  | jg18346.t1_Dehydrogenase/reductase-SDR-family-member-13 | 0.574733 | 3.21E-05 |  |
|  | jg10540.t1_nek-protein-kinase; jg18130.t1_nek-protein-kinase | 0.574115 | 0.000426 |  |
|  | jg13445.t2_galactonolactone-dehydrogenase | 0.573136 | 0.021016 |  |
|  | jg9825.t1_Proactivator-polypeptide | 0.571634 | 2.71E-05 |  |
|  | jg5213.t1_putative-endo-13-beta-glucanase | 0.566418 | 0 |  |
|  | jg11985.t1_Acyl-CoA-dehydrogenase | 0.564424 | 2.56E-05 |  |
|  | jg11729.t1_hypothetical-protein-PHYSODRAFT_287011 | 0.560077 | 0.010963 |  |
|  | jg19740.t1_SDR-family-oxidoreductase | 0.559967 | 0.010922 |  |
|  | jg11645.t1_Dimeric-alpha-beta-barrel | 0.559862 | 0.008623 |  |
|  | jg18384.t1_citrate-(Si)-synthase-eukaryotic | 0.559505 | 0 |  |
|  | jg13268.t1_Mitochondrial/chloroplast-ribosomal-protein-36a | 0.558567 | 0.015652 |  |
|  | jg7802.t4_Polyribonucleotide-nucleotidyltransferase | 0.558537 | 0.015589 |  |
|  | jg12774.t1_39s-ribosomal-protein-mitochondrial-like | 0.556516 | 0.022731 |  |
|  | jg9627.t1_Acetylornithine-aminotransferase-mitochondrial-precursor | 0.556341 | 0 |  |
|  | jg2096.t1_Ribosomal-protein-S24/S35-mitochondrial-conserved-domain; jg5491.t1_Ribosomal-protein-S24/S35-mitochondrial-conserved-domain | 0.555582 | 0 |  |
|  | jg8867.t1_SBDS-family-rRNA-metabolism-protein | 0.554118 | 0.001217 |  |
|  | jg4851.t1_succinate-semialdehyde-dehydrogenase-mitochondrial-precursor | 0.554023 | 0 |  |
|  | jg7827.t1_serine/arginine-rich-SC35-like-splicing-factor-SCL30 | 0.552879 | 0.000123 |  |
|  | jg13334.t1_3-hydroxyisobutyrate-dehydrogenase; jg16333.t1_3-hydroxyisobutyrate-dehydrogenase | 0.551743 | 0 |  |
|  | jg13381.t1_Methylenetetrahydrofolate-reductase | 0.545335 | 2.84E-05 |  |
|  | jg1144.t1_isocitrate-dehydrogenase-NADP-dependent | 0.544745 | 0 |  |
|  | jg12182.t1_FKBP-type-peptidyl-prolyl-cis-trans-isomerase | 0.543747 | 0 |  |
|  | jg15822.t1_23-bisphosphoglycerate-dependent-phosphoglycerate-mutase | 0.543637 | 0.000115 |  |
|  | jg3114.t1_putative-exo-13-beta-glucanase | 0.542172 | 0.00153 |  |
|  | jg19534.t1_Phosphatidate-cytidylyltransferase | 0.540294 | 0.000118 |  |
|  | jg8221.t3_Paired-amphipathic-helix-protein-Sin3; jg8221.t4_Paired-amphipathic-helix-protein-Sin3 | 0.539986 | 0.001856 |  |
|  | jg19160.t1_antibiotic-biosynthesis-monooxygenase; jg19160.t2_antibiotic-biosynthesis-monooxygenase; jg19167.t1_antibiotic-biosynthesis-monooxygenase | 0.539811 | 0.000369 |  |
|  | jg19335.t1_Zinc-finger-CCCH-type | 0.539044 | 0.003449 |  |
|  | jg1517.t1_GrpE | 0.538757 | 3.11E-05 |  |
|  | jg14843.t1_estradiol-17beta-dehydrogenase | 0.537411 | 0.000376 |  |
|  | jg16016.t1_Peroxisomal-(S)-2-hydroxy-acid-oxidase | 0.536796 | 0 |  |
|  | jg19290.t1_Alcohol-dehydrogenase | 0.533915 | 0.049341 |  |
|  | jg18587.t1_hypothetical-protein-PHPALM_37367 | 0.529328 | 8.14E-05 |  |
|  | jg17444.t1_La-related-protein-4 | 0.529291 | 0.000932 |  |
|  | jg8475.t1_acyl-CoA-dehydrogenase/oxidase | 0.522179 | 0.000686 |  |
|  | jg14199.t1_Transmembrane-emp24-domain-containing-protein-1 | 0.518471 | 0.001205 |  |
|  | jg17450.t1_rna-binding | 0.515455 | 0.009173 |  |
|  | jg8826.t1_Cytochrome-c-type-heme-lyase | 0.514009 | 2.68E-05 |  |
|  | jg12275.t1_succinyl-CoA-ligase-beta-chain-mitochondrial-precursor | 0.512436 | 0 |  |
|  | jg11003.t1_Bromodomain-containing-protein-9 | 0.511564 | 0.041879 |  |
|  | jg16277.t1_ribosomal-protein-L1 | 0.510533 | 2.64E-05 |  |
|  | jg15821.t1_F21B23.6-PROTEIN-RELATED | 0.509413 | 2.76E-05 |  |
|  | jg11477.t1_myb-like-DNA-binding-protein-putative | 0.508831 | 0.018052 |  |
|  | jg15630.t1_Ribosomal-protein-S6 | 0.505326 | 0.001136 |  |
|  | jg14093.t1_D-aspartate-oxidase | 0.502766 | 0.000985 |  |
|  | jg6158.t1_rRNA-2'-O-methyltransferase-fibrillarin | 0.502228 | 0 |  |
|  | jg16575.t1_Vacuolar-processing-enzyme | 0.4951 | 0.002175 |  |
|  | jg8088.t1_nuclear-autoantigenic-sperm | 0.494465 | 0.004566 |  |
|  | jg16739.t1_Carbonic-anhydrase | 0.493482 | 0.000145 |  |
|  | jg16385.t1_bet1-homolog | 0.49326 | 0.021448 |  |
|  | jg6923.t1_putative-mitochondrial-protein | 0.492915 | 0.02218 |  |
|  | jg15620.t1_Phosphomannomutase | 0.492091 | 0 |  |
|  | jg7361.t1_Friend-of-PRMT1-duplication | 0.491726 | 0.022211 |  |
|  | jg2269.t1_FK506-binding-protein-2 | 0.490312 | 2.61E-05 |  |
|  | jg1498.t1_Histone-binding-protein-RBBP4 | 0.489944 | 0.000715 |  |
|  | jg19694.t1_short-chain-dehydrogenase-putative | 0.487716 | 0.011441 |  |
|  | jg18643.t1_methylenetetrahydrofolate-reductase | 0.484603 | 0.000115 |  |
|  | jg5511.t1_Alternative-oxidase | 0.478629 | 3.03E-05 |  |
|  | jg11114.t1_NAD-dependent-malic-enzyme | 0.475847 | 0 |  |
|  | jg3671.t1_ribonucleoprotein-associated-protein | 0.475343 | 0.00014 |  |
|  | jg11180.t1_Guanylate-kinase; jg16449.t1_Guanylate-kinase | 0.471481 | 0.0058 |  |
|  | jg14611.t1_Enoyl-CoA-hydratase | 0.471452 | 0 |  |
|  | jg9551.t1_chaperonin-GroL | 0.471219 | 0 |  |
|  | jg8659.t1_ubiquitin-family-protein-putative | 0.471081 | 0.00113 |  |
|  | jg13514.t1_Lupus-La-protein | 0.470567 | 0.00041 |  |
|  | jg15963.t1_Calnexin | 0.468433 | 0.002517 |  |
|  | jg2636.t1_CorA-Metal-Ion-transporter-(MIT)-Family | 0.46755 | 0.000864 |  |
|  | jg1659.t1_adenylosuccinate-synthetase | 0.466745 | 0 |  |
|  | jg15160.t1_Glutamate-synthase | 0.466607 | 0 |  |
|  | jg14096.t1_oxidoreductase-htatip2-like-protein | 0.464809 | 0 |  |
|  | jg13946.t1_Enolase | 0.464001 | 0 |  |
|  | jg19762.t1_Ribosomal-protein-S15 | 0.462088 | 0.014848 |  |
|  | jg7452.t1_ribosomal-protein-L13 | 0.461833 | 0 |  |
|  | jg16550.t1_Stomatin-like-protein | 0.458525 | 3.02E-05 |  |
|  | jg17533.t1_Adenylate-kinase | 0.458444 | 0.000153 |  |
|  | jg3003.t1_ Mitochondrial-import-inner-membrane-translocase-subunit-Tim8 | 0.453992 | 0 |  |
|  | jg15681.t1_ribosomal-protein-L19 | 0.451093 | 0.005668 |  |
|  | jg2033.t1_NADP-specific-glutamate-dehydrogenase | 0.449054 | 0 |  |
|  | jg10900.t1_hypothetical-protein-PHPALM_11024 | 0.445886 | 0.00131 |  |
|  | jg6199.t1_Exopolysaccharide-phosphotransferase | 0.445087 | 0.000911 |  |
|  | jg10973.t1_Ribose-phosphate-pyrophosphokinase | 0.442767 | 3.24E-05 |  |
|  | jg12185.t1_Ribonucleoside-diphosphate-reductase-large-subunit | 0.439833 | 0.004519 |  |
|  | jg18663.t1_Argininosuccinate-lyase-2 | 0.439348 | 8.56E-05 |  |
|  | jg2202.t1_Hsp70-like-protein | 0.439329 | 0.000368 |  |
|  | jg5338.t1_TKL-protein-kinase | 0.438466 | 0.027347 |  |
|  | jg17023.t1_nucleotide-binding-protein-1 | 0.434991 | 0.041465 |  |
|  | jg12257.t1_acetyl-CoA-carboxylase-biotin-carboxylase-subunit | 0.429898 | 0 |  |
|  | jg5656.t1_hypothetical-protein-PC110_g10442 | 0.429132 | 0.003366 |  |
|  | jg2559.t1_glutaredoxin | 0.425789 | 0.000147 |  |
|  | jg3601.t1_Grx4-family-monothiol-glutaredoxin | 0.424813 | 7.87E-05 |  |
|  | jg13570.t1_glutathione-S-transferase-putative | 0.424708 | 0.001126 |  |
|  | jg10658.t1_Acyl-protein-thioesterase-1 | 0.424459 | 0.029869 |  |
|  | jg5212.t1_serine/arginine-rich-splicing-factor-2-like | 0.422288 | 0.000859 |  |
|  | jg7442.t1_acetolactate-synthase-small-subunit | 0.422191 | 0 |  |
|  | jg50.t1_H/ACA-ribonucleoprotein-complex-subunit-4 | 0.421305 | 0.002626 |  |
|  | jg667.t1_protein-disulfide-isomerase-domain | 0.420662 | 8.24E-05 |  |
|  | jg5181.t1_hypothetical-protein-F442_05017 | 0.418826 | 0.002054 |  |
|  | jg5595.t2_sulfite-reductase-(NADPH)-hemoprotein-beta-component | 0.418694 | 0 |  |
|  | jg8538.t1_Scaffold/matrix-specific-factor-hnRNP-U/SAF-A-contains-SPRY-domain | 0.418464 | 0.000119 |  |
|  | jg6745.t1_hypothetical-protein-PHYSODRAFT_526119 | 0.418051 | 0.001971 |  |
|  | jg19570.t1_myb-like-DNA-binding-protein-putative; jg19570.t2_myb-like-DNA-binding-protein-putative; jg19570.t3_myb-like-DNA-binding-protein-putative | 0.417874 | 8.38E-05 |  |
|  | jg7420.t1_lectin-putative | 0.417004 | 0.004 |  |
|  | jg19180.t1_ATP-dependent-protease-subunit-HslV | 0.41684 | 0.000117 |  |
|  | jg10545.t1_ATP-dependent-Clp-endopeptidase-proteolytic-subunit-ClpP; jg18126.t1_ATP-dependent-Clp-endopeptidase-proteolytic-subunit-ClpP | 0.416549 | 0.001668 |  |
|  | jg13194.t1_Glutathione-S-transferase | 0.416144 | 0.04632 |  |
|  | jg19665.t1_protein-transport-protein-SEC13 | 0.415621 | 0.009486 |  |
|  | jg12396.t1_50s-ribosomal-protein-l24; jg2506.t1_50s-ribosomal-protein-l24 | 0.413864 | 0.047755 |  |
|  | jg9044.t1_acyl-CoA-desaturase | 0.411283 | 0.035238 |  |
|  | jg2659.t1_carbohydrate-esterase | 0.411254 | 0.001825 |  |
|  | jg13870.t1_Endoplasmic-reticulum-Golgi-intermediate-compartment-protein | 0.410739 | 0.003579 |  |
|  | jg13536.t1_nitrilase/cyanide-hydratase-and-apolipo-protein-N-acyltransferase-like-protein | 0.410681 | 0 |  |
|  | jg11103.t1_Decapping-enzyme-complex-component-DCP1 | 0.409291 | 0.003009 |  |
|  | jg18370.t1_N2N2-dimethylguanosine-tRNA-methyltransferase | 0.409213 | 0.008441 |  |
|  | jg17289.t1_ornithine-aminotransferase-mitochondrial | 0.406035 | 0 |  |
|  | jg10932.t1_Fumarylacetoacetase | 0.403898 | 0 |  |
|  | jg10531.t1_Ribosomal-protein-L15; jg18137.t1_Ribosomal-protein-L15 | 0.403591 | 0.017221 |  |
|  | jg5807.t1_protein-disulfide-isomerase-domain | 0.403496 | 0 |  |
|  | jg15608.t1_arginine-serine-rich-splicing-factor-rsp40-like | 0.403001 | 0.010914 |  |
|  | jg17570.t2_Neprilysin-CD10-peptidase | 0.402621 | 0.000235 |  |
|  | jg4461.t1_eukaryotic-peptide-chain-release-factor-subunit-1 | 0.402424 | 0 |  |
|  | jg18412.t1_succinyl-CoA-ligase-[GDP-forming]-subunit-alpha-mitochondrial | 0.40181 | 0 |  |
|  | jg3930.t1_Nitric-oxide-synthase | 0.400602 | 0.00069 |  |
|  | jg19887.t1_ATP:cob(I)alamin-adenosyltransferase | 0.394773 | 0.044726 |  |
|  | jg2373.t1_Predicted-regulator-of-the-ubiquitin-pathway-(contains-UAS-and-UBX-domains) | 0.3934 | 0.03365 |  |
|  | jg10753.t1_3-hydroxyisobutyryl-CoA-hydrolase | 0.393118 | 0 |  |
|  | jg19422.t1_synaptobrevin-like-protein; jg2478.t1_synaptobrevin-like-protein | 0.393078 | 6.13E-05 |  |
|  | jg17401.t1_ribosomal-protein-L7/L12 | 0.392622 | 0.00038 |  |
|  | jg11260.t1_Serine-hydroxymethyltransferase-mitochondrial-precursor | 0.392408 | 0 |  |
|  | jg12971.t1_ATP-synthase-subunit-d | 0.390956 | 2.95E-05 |  |
|  | jg13547.t1_phosphoribosyldiphosphate-synthetase | 0.390511 | 0.007462 |  |
|  | jg15317.t1_Ribosomal-protein-S16 | 0.387459 | 0.004466 |  |
|  | jg18103.t1_ATP-dependent-protease-La; jg5893.t1_ATP-dependent-protease-La | 0.387327 | 0.001475 |  |
|  | jg19013.t1_Hydroxyacylglutathione-hydrolase | 0.387053 | 0 |  |
|  | jg11365.t1_ATP-synthase-subunit-beta-mitochondrial | 0.386699 | 0 |  |
|  | jg8498.t1_Carbamoyl-phosphate-synthase-large-subunit | 0.385924 | 0.000248 |  |
|  | jg881.t1_nitronate-monooxygenase | 0.384522 | 0.002653 |  |
|  | jg938.t1_hypothetical-cleavage-induced-protein | 0.383824 | 0 |  |
|  | jg17134.t1_UDP-glucose-4-epimerase-putative | 0.382065 | 0.002645 |  |
|  | jg15854.t1_peptidoglycan-binding-protein; jg3338.t1_peptidoglycan-binding-protein | 0.380993 | 0.006623 |  |
|  | jg17393.t1_Aconitate-hydratase-mitochondrial | 0.380692 | 0 |  |
|  | jg14460.t1_pyrroline-5-carboxylate-reductase; jg16224.t1_pyrroline-5-carboxylate-reductase | 0.379078 | 0.002379 |  |
|  | jg3509.t1_translation-elongation-factor-G; jg3509.t2_translation-elongation-factor-G | 0.377857 | 0.003025 |  |
|  | jg18997.t1_Glutathione-transferase-theta-class | 0.375194 | 0.000961 |  |
|  | jg3156.t1_hypothetical-protein-PHYSODRAFT_250450 | 0.37498 | 0.005312 |  |
|  | jg8014.t1_ Mitochondrial-import-inner-membrane-translocase-subunit-Tim13-B | 0.374798 | 0.004755 |  |
|  | jg12560.t1_Molecular-chaperone-(DnaJ-superfamily); jg1541.t1_Molecular-chaperone-(DnaJ-superfamily) | 0.374747 | 0.010187 |  |
|  | jg1737.t1_thioredoxin-1 | 0.37315 | 0 |  |
|  | jg18873.t1_Mitochondrial-Carrier-(MC)-Family | 0.372158 | 0.010284 |  |
|  | jg14702.t1_glutaredoxin | 0.370225 | 0.020452 |  |
|  | jg4526.t1_10-kda-chaperonin | 0.369074 | 0 |  |
|  | jg13506.t1_zz-type-zinc-finger-containing-protein | 0.369041 | 0.005784 |  |
|  | jg10969.t1_isochorismatase-putative | 0.368704 | 7.82E-05 |  |
|  | jg3670.t1_Mitochondrial-import-receptor-subunit-TOM70 | 0.366975 | 0.003696 |  |
|  | jg12155.t1_ectoine-synthase | 0.366782 | 0.009426 |  |
|  | jg7126.t1_carbamoyl-phosphate-synthase-large-subunit | 0.364896 | 0 |  |
|  | jg11247.t1_Heat-shock-protein-90-2 | 0.363858 | 3.00E-05 |  |
|  | jg2011.t1_Replication-factor-C-subunit-1 | 0.363376 | 0.028968 |  |
|  | jg14907.t1_Formate-dehydrogenase; jg14909.t1_Formate-dehydrogenase | 0.362722 | 0.000123 |  |
|  | jg1095.t1_PNS1-protein | 0.359458 | 0.003892 |  |
|  | jg647.t1_ubiquitin-fusion-degradation-protein-putative | 0.358423 | 0.00766 |  |
|  | jg13377.t1_S-(hydroxymethyl)glutathione-dehydrogenase/class-III-alcohol-dehydrogenase | 0.357678 | 0 |  |
|  | jg7780.t1_Transcription-initiation-factor-TFIID-subunit-BDF1-and-related-bromodomain-proteins | 0.357675 | 0.004597 |  |
|  | jg16963.t1_transitional-endoplasmic-reticulum-ATPase | 0.355739 | 0 |  |
|  | jg9028.t1_prefoldin-alpha-subunit | 0.355695 | 0.005776 |  |
|  | jg19269.t1_triosephosphate-isomerase/glyceraldehyde-3-phosphate-dehydrogenase | 0.354054 | 0 |  |
|  | jg14147.t1_32-trans-enoyl-CoA-isomerase-mitochondrial-precursor | 0.353941 | 0 |  |
|  | jg11635.t1_dihydroorotase-homodimeric-type | 0.352196 | 0.004084 |  |
|  | jg11048.t1_cell-division-protease-ftsH | 0.349881 | 0.002258 |  |
|  | jg10168.t1_prolyl-aminopeptidase | 0.349527 | 0.000465 |  |
|  | jg9010.t1_Mitochondrial-Carrier-(MC)-Family; jg9010.t2_Mitochondrial-Carrier-(MC)-Family | 0.34917 | 7.76E-05 |  |
|  | jg16626.t1_uroporphyrinogen-decarboxylase; jg8694.t1_uroporphyrinogen-decarboxylase | 0.348898 | 0.010271 |  |
|  | jg10026.t1_small-nuclear-ribonucleoprotein-splicing-factor | 0.346982 | 0.000234 |  |
|  | jg556.t1_ATPase-family-AAA-domain-containing-protein-3A | 0.345042 | 2.86E-05 |  |
|  | jg15815.t1_endoribonuclease-L-PSP-putative | 0.344957 | 0.000464 |  |
|  | jg17817.t1_Transcription-elongation-factor-S-II-central-domain | 0.344228 | 0.013899 |  |
|  | jg13442.t1_aldehyde-dehydrogenase-family-7-member-A1; jg13442.t2_aldehyde-dehydrogenase-family-7-member-A1; jg13442.t3_aldehyde-dehydrogenase-family-7-member-A1; jg13442.t4_aldehyde-dehydrogenase-family-7-member-A1 | 0.344183 | 0 |  |
|  | jg1762.t1_trifunctional-enzyme-subunit-beta-mitochondrial-precursor | 0.340981 | 2.94E-05 |  |
|  | jg5443.t1_GrxB-family-glutaredoxin | 0.340471 | 0.006715 |  |
|  | jg8643.t1_beta-glucosidase-putative | 0.339883 | 0.008345 |  |
|  | jg9816.t1_cytosol-aminopeptidase | 0.338731 | 0.000527 |  |
|  | jg14127.t1_RxLR-like-protein | 0.338626 | 0.001939 |  |
|  | jg19220.t1_MLN-STAR-and-related-lipid-binding-proteins | 0.338108 | 0.013333 |  |
|  | jg13990.t1_putative-GH30-glucocerebrosidase/glucosylceramidase-precursor | 0.338031 | 0.001605 |  |
|  | jg17354.t1_GrxB-family-glutaredoxin | 0.337512 | 0.005328 |  |
|  | jg17940.t1_enoyl-CoA-hydratase/isomerase-family-putative | 0.336154 | 0.000147 |  |
|  | jg3576.t1_Nucleotide-binding-protein | 0.333301 | 8.26E-05 |  |
|  | jg8464.t1_nuclear-pore-glycoprotein-putative | 0.332603 | 0.022629 |  |
|  | jg7411.t1_Dihydropteridine-reductase | 0.330482 | 0.014324 |  |
|  | jg12992.t1_60S-ribosomal-protein | 0.33016 | 6.15E-05 |  |
|  | jg5073.t1_Autophagocytosis-associated-protein | 0.329248 | 0.004952 |  |
|  | jg19364.t1_NADH-ubiquinone-oxidoreductase-subunit-putative | 0.329203 | 0.029018 |  |
|  | jg3935.t1_CMGC/CK2-protein-kinase | 0.329031 | 0.005655 |  |
|  | jg4338.t1_Sec62/63-complex-subunit-Sec66 | 0.328503 | 0.003371 |  |
|  | jg11452.t1_HAD-like-domain | 0.326022 | 3.38E-05 |  |
|  | jg5713.t1_ABCA1-lipid-exporter | 0.324929 | 0.005161 |  |
|  | jg16161.t1_nadh-dehydrogenase | 0.324242 | 0.001061 |  |
|  | jg17560.t1_serrate-rna-effector-molecule; jg17560.t2_serrate-rna-effector-molecule | 0.320847 | 0.018897 |  |
|  | jg9606.t2_ferredoxin-dependent-glutamate-synthase-putative | 0.319305 | 0.000858 |  |
|  | jg18454.t1_Glutathione-S-transferase-A | 0.318329 | 0.028987 |  |
|  | jg7672.t1_cellulose-binding-elicitor-lectin | 0.318065 | 0.001557 |  |
|  | jg19943.t1_Mitochondrial-import-receptor-subunit-TOM40-1 | 0.317479 | 0.001447 |  |
|  | jg3290.t1_ATP-synthase-F1-delta-subunit | 0.315494 | 3.04E-05 |  |
|  | jg14431.t1_predicted-protein | 0.312581 | 0.003949 |  |
|  | jg539.t1_Protease-2 | 0.311725 | 0.001063 |  |
|  | jg14444.t1_3-oxoacid-CoA-transferase-1 | 0.311701 | 0.000891 |  |
|  | jg11264.t1_amp-binding-protein | 0.309235 | 7.80E-05 |  |
|  | jg5579.t1_gamma-carbonic-dehydratase | 0.30833 | 0.000218 |  |
|  | jg18192.t1_choline-phosphate-cytidylyltransferase-B | 0.307771 | 0.019151 |  |
|  | jg11966.t1_succinate-dehydrogenase-[ubiquinone]-iron-sulfur-subunit-mitochondrial; jg16393.t1_succinate-dehydrogenase-[ubiquinone]-iron-sulfur-subunit-mitochondrial | 0.307674 | 0.000298 |  |
|  | jg15052.t1_Pyruvate-kinase | 0.307347 | 0.001058 |  |
|  | jg19060.t1_Mannitol-2-dehydrogenase | 0.305792 | 0.001361 |  |
|  | jg8946.t1_putative-mitochondrial-protein | 0.305048 | 0.026871 |  |
|  | jg16059.t1_protein-disulfide-isomerase-domain | 0.304718 | 0.001134 |  |
|  | jg14061.t1_Hydroxyacyl-coenzyme-A-dehydrogenase-mitochondrial-precursor | 0.304388 | 0.001821 |  |
|  | jg10559.t1_ribosomal-protein | 0.303095 | 0.004194 |  |
|  | jg17273.t1_Eukaryotic-translation-initiation-factor-3-subunit-F | 0.300315 | 0 |  |
|  | jg2865.t1_hypothetical-protein-PC110_g16 | 0.29935 | 0.007838 |  |
|  | jg19945.t1_whirly-isoform-1 | 0.297112 | 0.011002 |  |
|  | jg7840.t1_Eukaryotic-translation-initiation-factor-6 | 0.295858 | 2.80E-05 |  |
|  | jg2660.t1_Membrane-protein | 0.294013 | 0.012629 |  |
|  | jg5588.t1_Sulfite-reductase | 0.292306 | 0.001552 |  |
|  | jg8012.t1_Prefoldin-subunit-2 | 0.292142 | 0.006339 |  |
|  | jg2677.t1_prohibitin-putative | 0.290309 | 2.90E-05 |  |
|  | jg15257.t1_methylmalonate-semialdehyde-dehydrogenase-(acylating) | 0.290306 | 0.033321 |  |
|  | jg6032.t1_Mitochondrial-Carrier-(MC)-Family | 0.288311 | 0.041147 |  |
|  | jg9390.t1_Grx4-family-monothiol-glutaredoxin | 0.284355 | 0.015195 |  |
|  | jg7487.t1_COP9-signalosome-complex-subunit-putative | 0.284232 | 0.001949 |  |
|  | jg5599.t1_Maltose-O-acetyltransferase | 0.283329 | 0.000247 |  |
|  | jg8234.t1_Short/branched-chain-specific-acyl-CoA-dehydrogenase | 0.283258 | 0.015754 |  |
|  | jg16141.t1_PDZ-domain | 0.282592 | 0.049566 |  |
|  | jg13221.t1_hypothetical-protein-PHYSODRAFT_263989 | 0.28194 | 0.004171 |  |
|  | jg19510.t1_NmrA-like-family-protein | 0.281248 | 0.00158 |  |
|  | jg4840.t1_PERQ-amino-acid-rich-with-GYF-domain-containing-protein | 0.280519 | 0.028505 |  |
|  | jg16411.t1_protein-disulfide-isomerase-domain | 0.278706 | 3.17E-05 |  |
|  | jg13340.t1_GTP-cyclohydrolase-II; jg13340.t2_GTP-cyclohydrolase-II | 0.278434 | 7.84E-05 |  |
|  | jg3462.t1_Nucleoside-diphosphate-kinase-B | 0.278059 | 0 |  |
|  | jg19638.t1_hypothetical-protein-PHYSODRAFT_354903 | 0.276116 | 0.011259 |  |
|  | jg19125.t1_Acyl-CoA-synthetase-short-chain-family-member-3 | 0.273928 | 0.000138 |  |
|  | jg16612.t1_Memo-like-protein; jg8708.t1_Memo-like-protein | 0.272807 | 0.00664 |  |
|  | jg19019.t1_Poly(ADP-ribose)-polymerase-pme-5 | 0.271727 | 0.005784 |  |
|  | jg9009.t1_ATP-synthase-F1-gamma-subunit | 0.27154 | 8.31E-05 |  |
|  | jg18970.t1_Glutathione-transferase-theta-class; jg18974.t1_Glutathione-transferase-theta-class | 0.269778 | 0.000866 |  |
|  | jg14150.t1_DEAD/DEAH-box-RNA-helicase | 0.269748 | 0 |  |
|  | jg8491.t1_DNA-directed-RNA-polymerase-I-II-and-III-subunit-RPABC1 | 0.269617 | 0.005661 |  |
|  | jg5621.t1_tho-complex-subunit-4-like | 0.26954 | 0.043211 |  |
|  | jg12087.t1_Oxysterol-binding-protein-9; jg12111.t1_Oxysterol-binding-protein-9 | 0.269225 | 0.048993 |  |
|  | jg7667.t1_H---or-Na--translocating-F-type-V-type-and-A-type-ATPase-(F-ATPase)-Superfamily | 0.267532 | 0.00037 |  |
|  | jg7583.t1_importin-like-protein | 0.265617 | 0.002181 |  |
|  | jg16969.t1_NADH-UBIQUINONE-OXIDOREDUCTASE-PDSW-SUBUNIT | 0.265121 | 0.001401 |  |
|  | jg7722.t1_protein-disulfide-isomerase-domain | 0.261961 | 0.049717 |  |
|  | jg11760.t1_transport-protein-sec1-protein | 0.26081 | 0.002378 |  |
|  | jg7023.t1_formyl-coenzyme-A-transferase-putative | 0.260002 | 0.019244 |  |
|  | jg687.t1_RNA-recognition-motif-domain | 0.25965 | 0.004923 |  |
|  | jg13171.t1_Mitochondrial-Protein-Translocase-(MPT)-Family | 0.258746 | 0.001349 |  |
|  | jg10259.t1_aspartate-carbamoyltransferase | 0.25791 | 0.007232 |  |
|  | jg7985.t1_NADH:ubiquinone-oxidoreductase-NDUFB9/B22-subunit | 0.257019 | 0.000373 |  |
|  | jg923.t1_Eukaryotic-translation-initiation-factor-3-subunit-J | 0.256934 | 0.001819 |  |
|  | jg18624.t1_Glycine-cleavage-system-T-protein | 0.256861 | 0 |  |
|  | jg7174.t1_Proteasome-subunit-alpha-type-3 | 0.255498 | 0.026242 |  |
|  | jg5626.t1_mitochondrial-processing-peptidase-subunit-alpha-like | 0.255291 | 0.005031 |  |
|  | jg13795.t1_Thimet-oligopeptidase | 0.254849 | 0 |  |
|  | jg19061.t1_Mannitol-2-dehydrogenase | 0.251579 | 0.001062 |  |
|  | jg1278.t1_atp-synthase-subunit-mitochondrial | 0.251432 | 2.96E-05 |  |
|  | jg4002.t1_DnaJ-subfamily-C-member-2-protein | 0.25132 | 0.000972 |  |
|  | jg6792.t1_Glycosyl-transferase | 0.250134 | 0.005238 |  |
|  | jg15680.t1_Signal-recognition-particle | 0.249972 | 0.027812 |  |
|  | jg8504.t1_mitochondrion-protein | 0.249964 | 0 |  |
|  | jg19808.t1_GMP-reductase | 0.248571 | 0.000689 |  |
|  | jg11111.t1_Protein-phosphatase-1-regulatory-subunit-and-related-proteins | 0.248332 | 0.00906 |  |
|  | jg13695.t1_Heat-shock-protein-sti1 | 0.247931 | 0.002766 |  |
|  | jg12528.t1_3-deoxy-7-phosphoheptulonate-synthase | 0.247511 | 0.001578 |  |
|  | jg19696.t1_12-oxophytodienoate-reductase-putative | 0.246942 | 0.000281 |  |
|  | jg12461.t1_dihydroorotate-dehydrogenase-(fumarate) | 0.246736 | 0.004768 |  |
|  | jg10616.t1_lectin-putative; jg11501.t1_lectin-putative | 0.243764 | 0.001306 |  |
|  | jg2655.t1_60S-acidic-ribosomal-protein-P0 | 0.243436 | 0.006642 |  |
|  | jg3920.t1_40S-ribosomal-protein-S12 | 0.242924 | 0.004917 |  |
|  | jg18349.t1_Cell-division-protease-ftsH | 0.242731 | 0.002406 |  |
|  | jg90.t1_Pre-mRNA-processing-factor-19-2 | 0.239183 | 0.034239 |  |
|  | jg7516.t1_60s-acidic-ribosomal-protein | 0.239073 | 0.003081 |  |
|  | jg572.t1_H---or-Na--translocating-F-type-V-type-and-A-type-ATPase-(F-ATPase)-Superfamily | 0.239016 | 0.00182 |  |
|  | jg13365.t1_hypothetical-protein-PHYSODRAFT_541498 | 0.238708 | 0.006984 |  |
|  | jg7258.t1_glutathione-S-transferase-putative | 0.237637 | 0.000217 |  |
|  | jg10922.t1_Prefoldin-subunit-6 | 0.237516 | 0.001822 |  |
|  | jg14590.t1_dnaj-homolog-subfamily-b-member-4-like | 0.237019 | 0.038325 |  |
|  | jg13929.t1_Glutamine-synthetase | 0.234967 | 0.00491 |  |
|  | jg16652.t1_AhpC/TSA-family-Redoxin-putative; jg8674.t1_AhpC/TSA-family-Redoxin-putative | 0.234081 | 3.16E-05 |  |
|  | jg7030.t1_neutral-zinc-metallopeptidase | 0.233596 | 0.003609 |  |
|  | jg16018.t1_nuclear-mitotic-apparatus-protein-putative | 0.233049 | 0.008117 |  |
|  | jg18252.t1_Peroxisomal-24-dienoyl-CoA-reductase | 0.233004 | 0.013386 |  |
|  | jg11184.t2_PWWP-domain; jg16445.t1_PWWP-domain | 0.23291 | 0.017085 |  |
|  | jg2980.t1_nucleosome-assembly-protein-1; jg689.t1_nucleosome-assembly-protein-1 | 0.23261 | 0.010781 |  |
|  | jg18166.t1_Ubiquinone-biosynthesis-monooxygenase-COQ6 | 0.231577 | 0.000466 |  |
|  | jg11865.t1_NADH-dehydrogenase-1-alpha-subcomplex-subunit-13 | 0.229897 | 0.00521 |  |
|  | jg16511.t1_glucan-13-beta-glucosidase-putative; jg9079.t1_glucan-13-beta-glucosidase-putative | 0.22698 | 0 |  |
|  | jg17495.t1_NADH:ubiquinone-oxidoreductase-NDUFA6/B14-subunit | 0.225292 | 2.91E-05 |  |
|  | jg2820.t1_Glycine-dehydrogenase | 0.224048 | 0.000365 |  |
|  | jg11179.t1_guanylate-kinase | 0.223068 | 0.044918 |  |
|  | jg7693.t1_cysteine-tRNA-ligase | 0.221703 | 0.014258 |  |
|  | jg14455.t1_Kynurenine-oxoglutarate-transaminase; jg16218.t1_Kynurenine-oxoglutarate-transaminase | 0.219159 | 0.001136 |  |
|  | jg19211.t1_mitochondrial-pyruvate-dehydrogenase-kinase | 0.215219 | 0.000248 |  |
|  | jg16014.t1_ATP-synthase-subunit-delta' | 0.21272 | 0.000856 |  |
|  | jg15524.t1_Cytochrome-b-c1-complex-subunit-6; jg17000.t1_Cytochrome-b-c1-complex-subunit-6 | 0.210837 | 0.014336 |  |
|  | jg5031.t1_Replication-factor-C-subunit-5 | 0.210717 | 0.009422 |  |
|  | jg18432.t1_Enhancer-of-mRNA-decapping-protein | 0.208833 | 0.001669 |  |
|  | jg5077.t1_ornithine-carbamoyltransferase | 0.208826 | 0.000528 |  |
|  | jg16762.t1_hypothetical-protein-PPTG_08061 | 0.208129 | 0.000506 |  |
|  | jg7801.t1_Pre-mRNA-splicing-factor-SF2-like-protein | 0.206765 | 0.000867 |  |
|  | jg5055.t1_3-mercaptopyruvate-sulfurtransferase | 0.206507 | 0.000234 |  |
|  | jg19270.t1_glyceraldehyde-3-phosphate-dehydrogenase | 0.20558 | 0.000118 |  |
|  | jg4832.t1_Protoporphyrinogen-oxidase | 0.205239 | 0.047921 |  |
|  | jg16513.t1_clathrin-light-chain; jg9077.t1_clathrin-light-chain | 0.204966 | 0.001138 |  |
|  | jg17208.t1_cellulase-1-endo-14-beta-glucanase | 0.204547 | 0.005711 |  |
|  | jg17816.t1_Ribosomal-protein-S2 | 0.20364 | 0.00148 |  |
|  | jg19949.t1_RxLR-like-protein | 0.201426 | 0.003777 |  |
|  | jg4248.t1_NADH-dehydrogenase-[ubiquinone]-flavoprotein-1-mitochondrial | 0.197109 | 0.000409 |  |
|  | jg19066.t1_Glycoside-hydrolase | 0.196932 | 2.58E-05 |  |
|  | jg7252.t1_D-3-phosphoglycerate-dehydrogenase | 0.192098 | 0.005165 |  |
|  | jg566.t1_hypothetical-protein-PHYSODRAFT_354157 | 0.191099 | 0.005342 |  |
|  | jg11067.t1_5-methyltetrahydropteroyltriglutamate-homocysteine-S-methyltransferase | 0.190795 | 0.000829 |  |
|  | jg2818.t1_Armadillo-type-fold | 0.189641 | 0.031764 |  |
|  | jg18217.t1_NADH-dehydrogenase-[ubiquinone]-iron-sulfur-protein-8-mitochondrial | 0.18953 | 0.025844 |  |
|  | jg11155.t1_NADH-dehydrogenase; jg16476.t1_NADH-dehydrogenase | 0.189043 | 0.00054 |  |
|  | jg15646.t1_acetyl-CoA-acetyltransferase | 0.187718 | 0.003314 |  |
|  | jg8971.t1_Molecular-co-chaperone-STI1 | 0.187295 | 0.000217 |  |
|  | jg11116.t1_26S-proteasome-non-ATPase-regulatory-subunit-7 | 0.186902 | 0.004177 |  |
|  | jg1956.t2_elongation-factor-Tu | 0.186855 | 0.000374 |  |
|  | jg16805.t1_malate-dehydrogenase-NAD-dependent | 0.186473 | 0.00296 |  |
|  | jg3636.t1_26S-protease-regulatory-subunit-6B | 0.184997 | 0.003994 |  |
|  | jg19672.t1_Cysteine-synthase-A | 0.184315 | 0.001007 |  |
|  | jg7573.t1_hsp70-like-protein | 0.183916 | 0.000737 |  |
|  | jg12393.t1_C2-domain-containing-protein-putative; jg2503.t1_C2-domain-containing-protein-putative | 0.183185 | 0.002051 |  |
|  | jg4169.t1_Coatomer-subunit-delta | 0.180607 | 0.007848 |  |
|  | jg7492.t1_aldehyde-dehydrogenase-putative | 0.179284 | 0.005346 |  |
|  | jg341.t1_Dynactin-5 | 0.178621 | 0.001533 |  |
|  | jg6338.t1_isoleucine-tRNA-ligase | 0.178127 | 8.02E-05 |  |
|  | jg10642.t1_hypothetical-protein-PHYSODRAFT_359351-partial | 0.177534 | 0.047932 |  |
|  | jg14928.t1_serine-protease-family-S08A-putative | 0.175236 | 0.004078 |  |
|  | jg8580.t1_serine-protease-family-S10-putative | 0.174523 | 0.001379 |  |
|  | jg7374.t1_sorting-and-assembly-machinery-component-50 | 0.174338 | 0.027289 |  |
|  | jg3867.t1_Phosphoserine-aminotransferase; jg3867.t2_Phosphoserine-aminotransferase | 0.174263 | 0.000142 |  |
|  | jg4761.t1_Branched-chain-amino-acid-aminotransferase | 0.173058 | 0.000691 |  |
|  | jg12790.t1_succinate-dehydrogenase-[ubiquinone]-flavoprotein-subunit-1-mitochondrial | 0.170357 | 2.56E-05 |  |
|  | jg11489.t1_ras-related-protein-rab-21-like | 0.169073 | 0.03073 |  |
|  | jg6114.t1_Gamma-glutamyl-hydrolase | 0.167663 | 0.023414 |  |
|  | jg4686.t1_Acyl-CoA-synthetase-family-member-3 | 0.167035 | 0.00206 |  |
|  | jg1743.t1_Eukaryotic-translation-initiation-factor-3-subunit-G | 0.165272 | 0.000264 |  |
|  | jg18203.t1_Prolyl-endopeptidase; jg18203.t2_Prolyl-endopeptidase | 0.162504 | 0.010907 |  |
|  | jg7622.t1_protein-transporter-Sec31A-putative | 0.162205 | 0.003783 |  |
|  | jg12340.t1_DNA-topoisomerase-2-DNA-gyrase-B | 0.161022 | 0.045316 |  |
|  | jg2654.t1_Ethanolamine-phosphate-cytidylyltransferase; jg5739.t1_Ethanolamine-phosphate-cytidylyltransferase | 0.160612 | 0.012184 |  |
|  | jg14808.t1_26S-proteasome-non-ATPase-regulatory-subunit | 0.159009 | 0.000247 |  |
|  | jg9524.t1_chaperone-DnaK | 0.158313 | 0.000372 |  |
|  | jg16384.t1_protein-phosphatase-2C-putative | 0.156825 | 0.003044 |  |
|  | jg19909.t1_DNA-directed-RNA-polymerase-II-subunit-RPB1 | 0.156689 | 0.032826 |  |
|  | jg3256.t1_Carbohydrate-binding-protein; jg9223.t1_Carbohydrate-binding-protein | 0.155949 | 0.000282 |  |
|  | jg4329.t1_pyruvate-dehydrogenase-complex-dihydrolipoamide-acetyltransferase | 0.1555 | 0.001119 |  |
|  | jg10161.t1_peptidase-putative | 0.155335 | 0.013813 |  |
|  | jg14489.t1_DEAD/DEAH-box-RNA-helicase | 0.15415 | 0.00263 |  |
|  | jg2893.t1_6-phosphogluconate-dehydrogenase-(decarboxylating) | 0.153311 | 0 |  |
|  | jg8825.t1_40S-ribosomal-protein-S6 | 0.152529 | 0.000479 |  |
|  | jg17482.t1_papain-like-cysteine-protease-C1 | 0.151483 | 2.59E-05 |  |
|  | jg19680.t1_40S-ribosomal-protein-S17 | 0.149602 | 0.008078 |  |
|  | jg11143.t1_Creatine-kinase-B-type | 0.148528 | 0.043597 |  |
|  | jg18686.t1_copper/zinc-superoxide-dismutase | 0.145953 | 0.005787 |  |
|  | jg18516.t1_Putative-methyltransferase; jg18516.t2_putative-methyltransferase-putative | 0.145763 | 0.048765 |  |
|  | jg15279.t1_Trans-2-enoyl-CoA-reductase | 0.144877 | 0.00012 |  |
|  | jg4404.t1_eukaryotic-translation-initiation-factor-3 | 0.14448 | 0.021144 |  |
|  | jg5980.t1_5-methyltetrahydropteroyltriglutamate-homocysteine-S-methyltransferase | 0.140516 | 0.001213 |  |
|  | jg10988.t1_Selenide-water-diKinase | 0.138174 | 6.29E-05 |  |
|  | jg3483.t1_Long-chain-fatty-acid-CoA-ligase | 0.13721 | 7.93E-05 |  |
|  | jg15951.t1_choline-dehydrogenase | 0.135906 | 0.016359 |  |
|  | jg11280.t1_Dethiobiotin-synthase | 0.135556 | 0.016909 |  |
|  | jg13040.t1_transcription-factor-BTF3-like-protein | 0.135268 | 0.005147 |  |
|  | jg15611.t1_fatty-acid-oxidation-complex-alpha-subunit-mitochondrial | 0.135234 | 0.003375 |  |
|  | jg10907.t1_Hsp90-like-protein | 0.133383 | 0.016747 |  |
|  | jg15937.t1_Ketol-acid-reductoisomerase | 0.13094 | 2.77E-05 |  |
|  | jg1411.t1_hsp70-like-protein | 0.12762 | 0.006351 |  |
|  | jg3879.t1_Glucose-6-phosphate-dehydrogenase | 0.126927 | 0.000863 |  |
|  | jg11054.t1_Ran-specific-GTPase-activating-protein | 0.12686 | 0.015185 |  |
|  | jg18758.t1_Phosphoglycerate-dehydrogenase | 0.125694 | 0.000756 |  |
|  | jg5712.t1_AP-1-complex-subunit-beta | 0.124587 | 0.034676 |  |
|  | jg12147.t1_WD40/YVTN-repeat-like-containing-domain | 0.123742 | 0.006658 |  |
|  | jg6929.t1_tryptophan-synthase-alpha-subunit | 0.123603 | 0.026657 |  |
|  | jg12199.t1_GlucoKinase | 0.122333 | 0.008044 |  |
|  | jg773.t1_14-3-3-like-protein | 0.122175 | 0.00113 |  |
|  | jg8018.t1_Ndufa5-NADH-ubiquinone-oxidoreductase-subunit | 0.121943 | 0.023477 |  |
|  | jg6132.t1_protein-MGARP-isoform-X1 | 0.121298 | 0.01725 |  |
|  | jg1816.t1_S-phase-kinase-associated-protein-1A | 0.118871 | 0.012339 |  |
|  | jg13111.t1_Phosphoribosylaminoimidazole-carboxylase-ATPase-subunit | 0.117006 | 0.033245 |  |
|  | jg18377.t1_26S-protease-regulatory-subunit-7 | 0.116218 | 0.000677 |  |
|  | jg9837.t1_Mitochondrial-processing-peptidase-subunit-alpha | 0.112703 | 0.000121 |  |
|  | jg2890.t1_Lysosomal-alpha-mannosidase | 0.111719 | 0.027496 |  |
|  | jg12968.t1_ribosomal-ubiquitin-protein-RPL40; jg13332.t1_polyubiquitin-C; jg16335.t1_Polyubiquitin; jg3766.t1_Polyubiquitin; jg9042.t1_ubiquitin-40s-ribosomal-protein-s27a | 0.110076 | 0.014735 |  |
|  | jg7488.t1_serine-tRNA-ligase | 0.11005 | 0.000231 |  |
|  | jg8002.t1_protein-transport-protein-sec61-subunit-beta-like | 0.108756 | 0.004095 |  |
|  | jg11730.t1_argininosuccinate-lyase | 0.104953 | 0.002721 |  |
|  | jg7598.t1_ATP-dependent-RNA-helicase-uap56 | 0.104646 | 0.00201 |  |
|  | jg778.t1_NADH-cytochrome-b5-reductase-putative | 0.103075 | 0.012647 |  |
|  | jg7005.t1_40S-ribosomal-protein-S10 | 0.101976 | 0.007241 |  |
|  | jg6938.t1_26S-protease-regulatory-subunit-10B | 0.10166 | 0.002765 |  |
|  | jg14724.t1_60S-ribosomal-protein-L31 | 0.101564 | 0.010952 |  |
|  | jg13050.t1_Nucleoside-diphosphate-kinase-B | 0.098112 | 0.00534 |  |
|  | jg11434.t1_40S-ribosomal-protein-S3a | 0.097393 | 0.004417 |  |
|  | jg3598.t1_40S-ribosomal-protein | 0.09688 | 0.003356 |  |
|  | jg8025.t1_40S-ribosomal-protein-S19-3 | 0.09687 | 0.001828 |  |
|  | jg10667.t1_proteasome-subunit-beta-type-6-putative | 0.09474 | 0.000141 |  |
|  | jg7089.t1_Prohibitin-2; jg8294.t1_Prohibitin-2 | 0.093801 | 0.006359 |  |
|  | jg19410.t1_26S-proteasome-non-ATPase-regulatory-subunit-putative | 0.093691 | 0.035243 |  |
|  | jg7090.t1_ribosomal-protein-S4; jg8293.t1_ribosomal-protein-S4 | 0.093136 | 0.004242 |  |
|  | jg16271.t1_26s-protease-regulatory-subunit-4; jg615.t1_26s-protease-regulatory-subunit-4 | 0.091692 | 0.015585 |  |
|  | jg10902.t1_50S-ribosomal-protein-L36e | 0.091047 | 0.028134 |  |
|  | jg6081.t1_40S-ribosomal-protein-S20 | 0.088865 | 0.015714 |  |
|  | jg14776.t1_40S-ribosomal-protein-S25 | 0.08523 | 0.002272 |  |
|  | jg6165.t1_ribosomal-protein-L29 | 0.084876 | 0.009246 |  |
|  | jg11780.t1_proteasome-subunit-alpha-type-2-A | 0.082612 | 0.012452 |  |
|  | jg7285.t1_Alpha-soluble-NSF-attachment-protein | 0.078327 | 0.013824 |  |
|  | jg10048.t1_alanine-tRNA-ligase | 0.076775 | 0.003835 |  |
|  | jg1343.t1_26S-proteasome-non-ATPase-regulatory-subunit-13 | 0.076328 | 0.010973 |  |
|  | jg13927.t1_endo-13(4)-beta-glucanase-putative | 0.076172 | 0.029719 |  |
|  | jg19509.t1_NmrA-like-family-protein-putative | 0.074772 | 0.026141 |  |
|  | jg6909.t1_Mitochondrial-processing-peptidase-subunit-beta | 0.070694 | 0.001531 |  |
|  | jg10659.t1_Voltage-gated-potassium-channel-subunit-beta | 0.06718 | 0.008287 |  |
|  | jg17952.t1_T-complex-protein-1-alpha-subunit | 0.067153 | 0.033001 |  |
|  | jg1804.t1_polyadenylate-binding-protein-1-B | 0.066769 | 0.002521 |  |
|  | jg4970.t1_Inositol-3-phosphate-synthase | 0.059645 | 0.003801 |  |
|  | jg13368.t1_Dihydrodipicolinate-synthase | 0.051985 | 0.033092 |  |
|  | jg11375.t1_Fumarate-hydratase-mitochondrial | 0.048747 | 0.006049 |  |
|  | jg10347.t1_T-complex-protein-1-beta-subunit | 0.046341 | 0.048162 |  |
|  | jg10060.t1_rab-GDP-dissociation-inhibitor-alpha | 0.045366 | 0.034572 |  |
|  | jg10873.t1_26S-proteasome-non-ATPase-regulatory-subunit-12 | 0.038292 | 0.031392 |  |

TYANOVA, S., TEMU, T., SINITCYN, P., CARLSON, A., HEIN, M. Y., GEIGER, T., MANN, M. & COX, J. 2016. The Perseus computational platform for comprehensive analysis of (prote)omics data. *Nat Methods,* 13**,** 731-40.
