# Supplementary material for: Phosphite inhibits Phytophthora cinnamomi by downregulating oxidoreductases and disrupting energy metabolism
Source: Front Microbiol. 2025 Aug 25;16:1632726. doi: 10.3389/fmicb.2025.1632726 (PMC12414984; doi:10.3389/fmicb.2025.1632726)
Supplement: Supplementary file 3 [file Table_2.docx]

**Supplementary Table S2.** Functional enrichment of significantly downregulated proteins (log_2_FC < –0.58). Gene Ontology (GO), KEGG (Kyoto Encyclopaedia of Genes and Genomes) pathway, annotated keyword (UniProt), and reactome pathway enrichment analyses were performed against the background list of all expressed proteins using the ‘Analysis’ tab in the STRING-DB v12.0 (Szklarczyk et al., 2023), with default settings and an FDR threshold of < 0.05.

| **Category** | **Term ID** | **Term description** | **Observed gene count** | **Background gene count** | **Strength** | **Signal** | **FDR** |
| --- | --- | --- | --- | --- | --- | --- | --- |
| GO Function | GO:0016491 | Oxidoreductase activity | 49 | 281 | 0.37 | 0.65 | 1.60E-05 |
| GO Function | GO:0016614 | Oxidoreductase activity, acting on CH-OH group of donors | 17 | 64 | 0.56 | 0.48 | 0.0057 |
| GO Function | GO:0016616 | Oxidoreductase activity, acting on the CH-OH group of donors, NAD or NADP as acceptor | 16 | 56 | 0.59 | 0.49 | 0.0057 |
| KEGG Pathways | map00010 | Glycolysis / Gluconeogenesis | 20 | 75 | 0.56 | 0.64 | 0.00038 |
| KEGG Pathways | map00051 | Fructose and mannose metabolism | 10 | 18 | 0.88 | 0.77 | 0.00045 |
| KEGG Pathways | map01100 | Metabolic pathways | 85 | 776 | 0.17 | 0.42 | 0.00058 |
| KEGG Pathways | map00620 | Pyruvate metabolism | 12 | 50 | 0.51 | 0.37 | 0.022 |
| KEGG Pathways | map01110 | Biosynthesis of secondary metabolites | 38 | 307 | 0.22 | 0.29 | 0.0303 |
| KEGG Pathways | map00030 | Pentose phosphate pathway | 7 | 21 | 0.66 | 0.35 | 0.0364 |
| KEGG Pathways | map01200 | Carbon metabolism | 20 | 127 | 0.33 | 0.3 | 0.0364 |
| UniProt Annotated Keywords | KW-0812 | Transmembrane | 61 | 364 | 0.36 | 0.75 | 1.33E-07 |
| UniProt Annotated Keywords | KW-1133 | Transmembrane helix | 60 | 359 | 0.36 | 0.74 | 1.33E-07 |
| UniProt Annotated Keywords | KW-0472 | Membrane | 62 | 413 | 0.31 | 0.65 | 1.69E-06 |
