# Supplementary material for: Phosphite inhibits Phytophthora cinnamomi by downregulating oxidoreductases and disrupting energy metabolism
Source: Front Microbiol. 2025 Aug 25;16:1632726. doi: 10.3389/fmicb.2025.1632726 (PMC12414984; doi:10.3389/fmicb.2025.1632726)
Supplement: Supplementary file 4 [file Table_3.docx]

**Supplementary Table S3.** Functional enrichment of significantly upregulated proteins (log_2_FC > 0.58). Gene Ontology (GO), KEGG (Kyoto Encyclopaedia of Genes and Genomes) pathway, annotated keyword (UniProt), and reactome pathway enrichment analyses were performed against the background list of all expressed proteins using the ‘Analysis’ tab in the STRING-DB v12.0 (Szklarczyk et al., 2023), with default settings and an FDR threshold of < 0.05.

| **Category** | **Term ID** | **Term description** | **Observed gene count** | **Background gene count** | **Strength** | **Signal** | **FDR** |
| --- | --- | --- | --- | --- | --- | --- | --- |
| GO Process | GO:0044282 | Small molecule catabolic process | 19 | 61 | 0.55 | 0.4 | 0.0153 |
| GO Process | GO:0046395 | Carboxylic acid catabolic process | 17 | 53 | 0.56 | 0.4 | 0.0166 |
| GO Component | GO:0005739 | Mitochondrion | 43 | 218 | 0.35 | 0.52 | 0.00064 |
| KEGG Pathways | map01100 | Metabolic pathways | 96 | 776 | 0.15 | 0.33 | 0.01 |
| KEGG Pathways | map00280 | Valine, leucine and isoleucine degradation | 14 | 47 | 0.53 | 0.41 | 0.0141 |
| KEGG Pathways | map01212 | Fatty acid metabolism | 11 | 35 | 0.55 | 0.35 | 0.0305 |
| KEGG Pathways | map01110 | Biosynthesis of secondary metabolites | 44 | 307 | 0.21 | 0.28 | 0.0363 |
| Reactome Pathways | MAP-1268020 | Mitochondrial protein import | 11 | 20 | 0.79 | 0.55 | 0.0047 |
| Reactome Pathways | MAP-1430728 | Metabolism | 67 | 494 | 0.19 | 0.27 | 0.0391 |
| Reactome Pathways | MAP-9609507 | Protein localization | 17 | 47 | 0.61 | 0.52 | 0.0041 |
